# Novel *seco*-dammarane triterpenes with antimicrobial activity against plant pathogens from *Ailanthus altissima* petioles

**DOI:** 10.3389/fpls.2026.1888371

**Published:** 2026-08-11

**Authors:** Anna Cselőtey, Márton Baglyas, Panna Aschenbrenner, Zoltán Bozsó, Ildikó Schwarczinger, József Bakonyi, András Darcsi, Vesna Glavnik, Irena Vovk, Ágnes M. Móricz

**Affiliations:** 1 Plant Protection Institute, HUN-REN Centre for Agricultural Research, Budapest, Hungary; 2 Doctoral School, Semmelweis University, Budapest, Hungary; 3 Pharmaceutical Chemistry and Technology Department, National Center for Public Health and Pharmacy, Budapest, Hungary; 4 Laboratory for Food Chemistry, National Institute of Chemistry, Ljubljana, Slovenia

**Keywords:** antibacterial activity, antifungal activity, bioassay-guided isolation, phytopathogens, *seco*-dammarane triterpenes, TLC–effect-directed analysis, TLC–MS, tree of heaven

## Abstract

There is a growing demand for effective, environmentally friendly pesticides due to increasing environmental awareness and stricter regulations on chemical use. Natural products, including secondary metabolites of the invasive tree of heaven (*Ailanthus altissima*), could represent promising sources for biopesticide development. A bioassay-guided isolation workflow involving thin-layer chromatography (TLC)–*Bacillus subtilis* bioassay, TLC–high-resolution mass spectrometry (HRMS), and preparative flash column chromatography yielded three antimicrobial *seco*-dammarane triterpenes from the methanolic extract of *A. altissima* petioles, namely the known shoreic acid (**1**), and the previously undescribed foveolin C (**2**), and (20*S*,24*R*)-epoxy-25-hydroxy-A-homo-4-oxadammaran-3-one (**3**). Their structures were elucidated by nuclear magnetic resonance (NMR) spectroscopy, supported by high-resolution tandem mass spectrometry (HRMS/MS) and polarimetry. The presence of the hydroxylated, polyunsaturated fatty acids 13-hydroxy-9,11-octadecadienoic acid (13-HODE, **4**) and 9-hydroxy-10,12-octadecadienoic acid (9-HODE, **5**), previously isolated from *A. altissima* bark, was also confirmed in the petioles by high-performance liquid chromatography (HPLC)–HRMS/MS. To the best of our knowledge, compounds **2** and **3** are reported here as new natural products, while compounds **1**, **4**, and **5** were detected in *A. altissima* petioles for the first time. In microdilution assays, compounds **1**–**5** showed antimicrobial activity against *B. subtilis*, the plant pathogenic bacteria *Curtobacterium flaccumfaciens* pv. *flaccumfaciens*, *Clavibacter michiganensis*, and *Rhodococcus fascians*, as well as the plant pathogenic fungus *Fusarium graminearum*. Compound **1** exhibited the strongest antibacterial activity, especially against *C. michiganensis* and *R. fascians*, with minimal inhibitory concentration (MIC) values two-fold lower than those of the positive control neomycin. Compounds **2**–**5** were the most effective against *R. fascians*, with MIC values the same as or two-fold higher than those of neomycin. These results indicate that *A. altissima* petioles represent a promising source of antimicrobial phytochemicals. After proper structure optimization, they could serve as lead compounds for the development of novel antimicrobial agents against certain phytopathogens.

## 1 Introduction

*Ailanthus altissima* (Mill.) Swingle, commonly known as the tree of heaven, belongs to the family Simaroubaceae, which consists of approximately 120 species (Pirani et al., 2022). It is a dioecious tree originating from China and Vietnam that has become present on all continents except Antarctica (Kowarik and Säumel, 2007). In Europe, it was first introduced as an ornamental plant in the 18th century, and nowadays it is considered one of the most invasive plant species in many countries (Sladonja et al., 2015). It is mostly found in cities and disturbed areas, but also occurs in natural habitats, along transportation corridors, and the borders of agricultural fields (Kowarik and Säumel, 2007). The aggressive spread of the tree, due to its fast growth rate, high seed production, and lateral root sprouts has a negative impact on the local biodiversity (Demeter et al., 2021; Soler and Izquierdo, 2024). Allelochemicals produced by its roots, such as ailanthone, can also inhibit the growth of surrounding vegetation, thus endangering the native flora (Qureshi and Anwar, 2024). Its pest, the spotted lanternfly (*Lycorma delicatula*), which has already appeared in North America and other regions, could also threaten various plant species, including grapevine (*Vitis vinifera*) (Barringer and Ciafré, 2020). Different strategies, including mechanical, chemical, and biological control, have been applied in the management of *A. altissima*, with varying degrees of success (Soler and Izquierdo, 2024).

The dried bark of the tree has been widely used in traditional Asian medicine to treat various illnesses, including gastrointestinal infections, uterine bleeding, leukorrhea, and fever (Li et al., 2021). Until now, more than 221 compounds have been isolated and characterized from the bark such as alkaloids (Ohmoto et al., 1976), quassinoids (Kubota et al., 1996), triterpenoids (Hong et al., 2013), coumarins (Seon et al., 2005), flavonoids (Muhammad Abdur Rahman et al., 2023), lignans (Zhu et al., 2021), and volatile oils (Andonova et al., 2021). Several among these constituents have been reported to exhibit diverse biological activities. The most characteristic and widely studied components, the quassinoids, were found to exhibit antitumor (Yang et al., 2014; Bailly, 2020), anti-tuberculosis (Rahman et al., 1997), and herbicidal (Heisey, 1996) effects. Alkaloids, especially canthin-6-one derivatives, displayed antibacterial, antifungal, antiviral, and antimalarial activities (Dai et al., 2016; Farouil et al., 2022). The leaf extracts have been shown to be rich in flavonoids such as quercetin, kaempferol, apigenin, and luteolin with antioxidant, antibacterial, antifungal, and cytotoxic properties (Poljuha et al., 2017; Mohamed et al., 2021). In recent studies, steroids from the leaves were also found to be effective against bacteria and hepatocellular carcinoma cell lines (Poljuha et al., 2017; Gao et al., 2022).

Pesticides play an important role in modern agriculture. The increasing world population has led to a growing demand for food production, which would be difficult to meet without these chemicals for controlling weeds, insects, and fungi. However, the overuse of pesticides contributes to environmental pollution, negatively affects human health, and causes harm for non-target animals and plants (Tudi et al., 2021; Zúñiga-Venegas et al., 2022; Asefa et al., 2024). In addition, the number of pollinators has decreased due to certain herbicides and fungicides, posing a serious threat to ecosystem stability and crop production (Goulson et al., 2015; Basu et al., 2024). Therefore, it is necessary to find safer, more environmentally sustainable alternatives, for which natural products could be potential candidates. *A. altissima* could be a promising source of natural pesticides, due to its competitive and resilient nature (De Feo et al., 2003; Pedersini et al., 2011).

In our previous studies (Cselőtey et al., 2024; Vovk et al., 2026), bioprofiles, including antibacterial profiles of tree of heaven extracts from different plant parts were evaluated, and six antibacterial compounds were isolated and identified from the stem and trunk bark of *A. altissima*: (9*Z*,11*E*)-13-hydroxy-9,11-octadecadienoic acid (13-HODE), (10*E*,12*Z*)-9-hydroxy-10,12-octadecadienoic acid (9-HODE), hexadecanedioic acid, 16-hydroxyhexadecanoic acid, 16-feruloyloxypalmitic acid, and canthin-6-one. Continuing this research, the present work aimed for the isolation, identification, and characterization of antibacterial compounds present in the methanolic extracts of *A. altissima* petioles and determining their antibiotic profile against plant pathogens. The analytical workflow included thin-layer chromatography (TLC) coupled with *Bacillus subtilis* bioassays, chemical derivatization, and spectroscopic (UV, Vis, FLD) and spectrometric (heated electrospray ionization (HESI)-tandem high-resolution mass spectrometry (HRMS/MS)) techniques for screening and characterization of the compounds, normal- and reversed-phase flash column chromatography for bioassay-guided isolation. Flow injection analysis (FIA)–HR-HESI-MS/MS, polarimetry, and UV, attenuated total reflectance Fourier-transform infrared (ATR–FTIR), and nuclear magnetic resonance (NMR) spectroscopy were applied for structural identification. The antibacterial and antifungal activities of the isolates were evaluated by *in vitro* microplate assays against *B. subtilis*, and the plant pathogens *Clavibacter michiganensis*, *Curtobacterium flaccumfaciens* pv. *flaccumfaciens*, *Rhodococcus fascians*, and *Fusarium graminearum*.

## 2 Materials and methods

### 2.1 Materials

The 20 cm × 10 cm aluminum- and glass-backed TLC silica gel 60 F_254_ layers were purchased from Merck (Darmstadt, Germany). Acetone, *n*-hexane, ethyl acetate, chloroform (stabilized with 5–50 ppm amylene), isopropyl alcohol, toluene, and methanol were obtained from Molar Chemicals (Halásztelek, Hungary) or Reanal (Budapest, Hungary). Isopropyl acetate, neomycin, benomyl, *p*-anisaldehyde, chloroform-*d* (≥99.8 atom% D, containing 0.5 wt.% silver foil as stabilizer, and 0.03% (*V*/*V*) tetramethylsilane), and methanol-*d*_4_ (≥99.8 atom% D) were purchased from Sigma-Aldrich (Budapest, Hungary). Acetic acid was from Lach-Ner (Neratovice, Czech Republic), and ultrapure water was prepared by a Millipore Direct-Q 3 UV Water Purification System (Merck). Preparative silica gel (no. 60752, high-purity grade, 60 Å pore size, 230–400 mesh particle size) was acquired from Sigma-Aldrich (St. Louis, MO, USA). LC-MS grade methanol was supplied by VWR (Debrecen, Hungary). 3-(4,5-Dimethylthiazol-2-yl)-2,5-diphenyltetrazolium bromide (MTT) was acquired from Carl Roth (Karlsruhe, Germany).

*Bacillus subtilis* (F1276) was provided by József Farkas (Central Food Research Institute, Budapest, Hungary). *Clavibacter michiganensis* (NCAIM B.01813) and *Curtobacterium flaccumfaciens* pv. *flaccumfaciens* (NCAIM B.01609) were obtained from the National Collection of Agricultural and Industrial Microorganisms (NCAIM, Budapest, Hungary). *Fusarium graminearum* Schwabe (NCAIM F.00730), *Rhodococcus fascians* (NCAIM B.01608), *Dickeya chrysanthemi* (previously *Erwinia chrysanthemi*, NCAIM B.01392), *Pectobacterium carotovorum* ssp. *carotovorum* (previously *Erwinia carotovora* NCAIM B.01109), *Pectobacterium atrosepticum* (previously *Erwinia carotovora* ssp. *atroseptica*, NCAIM B.01611), and *Xanthomonas citri* pv. *malvacearum* (NCAIM B.01712) were purchased from NCAIM. Other tested Gram-negative bacterial strains were *Pseudomonas syringae* pv. *tomato* DC3000 (Cuppels, 1986), *Pseudomonas syringae* pv. *maculicola* Lux CDABE (Fan et al., 2008), and *Agrobacterium tumefaciens* [strain C58C1 (Deblaere et al., 1985)].

### 2.2 Plant material and sample preparation

The petioles of *A. altissima* were collected at three Hungarian locations, namely Harta (46°41′44″ N, 19°03′13″ E; 90 m a.s.l.), Leányfalu (47°42′11″ N, 19°04′25″ E; 254 m a.s.l.), and Balatongyörök (46°45′35″ N, 17°20′16″ E; 114 m a.s.l.), between May 2022 and October 2023 (**Supplementary Table 1**). The voucher specimens are available at the herbarium of the Plant Protection Institute, HUN-REN Centre for Agricultural Research, Budapest, Hungary, with deposition numbers PPI-MA-Aa-H18.3 and PPI-MA-Aa-H19.3, respectively. The samples were cleaned, dried at room temperature for a week, and then ground by a coffee grinder (Sencor SCG 2050RD, Říčany, Czech Republic). The powdered samples (1.5 g of each) were extracted with methanol (7.5 mL) at room temperature by using ultrasound-assisted extraction (15 min, in an ultrasonic bath [Sonorex Super RK 106, Bandelin, Berlin, Germany)] and analyzed by HPTLC after filtration (0.22 µm polyvinylidene fluoride (PVDF) syringe filter, Lab-Ex, Budapest, Hungary). For isolation, 150 g of each of the H18 and H19 samples was extracted separately three times with methanol (700 mL) at room temperature. The extracts were filtered (Whatman No. 2 filter paper, Sigma-Aldrich, Budapest, Hungary) and concentrated *in vacuo* at 40 °C by a rotary evaporator (Rotavapor R-134, Büchi, Flawil, Switzerland). Samples were stored at 4 °C before and after analysis.

### 2.3 TLC and TLC–*B. subtilis* bioassays

The plant extracts and fractions (10 µL) were applied onto the TLC plates by an Automatic TLC Sampler 3 (ATS3, CAMAG, Muttenz, Switzerland) or manually, using a 10 µL microsyringe (Hamilton, Bonaduz, Switzerland), as 5–7 mm bands with 8 mm distance from the lower plate edge. Separations were performed in a saturated (for 10 min without filter paper) twin trough chamber (20 cm × 10 cm, CAMAG) with toluene–isopropyl acetate–methanol (5:4:1, *V*/*V*). Plates were developed up to 90 mm from the lower edge in approximately 20 min. After development and drying in a stream of cold air for 5 min, images of the plates were documented with a digital camera (Cybershot DSC-HX60, Sony, Neu-Isenberg, Germany) under a UV lamp (CAMAG) at 254 nm and 366 nm. Post-chromatographic derivatization was performed by immersing the plates in *p*-anisaldehyde reagent [500 μL of *p*-anisaldehyde, 10 mL of acetic acid, 100 mL of methanol, and 5 mL of concentrated sulfuric acid (96%)], followed by heating for 5 min at 110 °C (Advanced Hot Plate, VWR, Debrecen, Hungary) and documentation at white light illumination (Vis, transmittance mode, 96891 Salobrena 2 LED lamp, Eglo Lux, Dunakeszi, Hungary).

The TLC–*B. subtilis* bioassays were performed as previously described (Móricz et al., 2015). *B. subtilis* was cultured by inoculating 10 mL of Luria–Bertani (LB) broth (10 g/L tryptone (Reanal), 5 g/L yeast extract (Scharlab, Barcelona, Spain), and 10 g/L sodium chloride (Reanal) with cells from an LB agar plate (5 g/L yeast extract, 10 g/L tryptone, 10 g/L sodium chloride, 15 g/L agar (Merck)). The culture was incubated at 37 °C for 6 h in an orbital shaker (ES-20, Orbital Shaker–Incubator, SIA Biosan, Riga, Latvia). The resulting suspension was then diluted with fresh LB medium to an optical density at 600 nm (OD_600_) of 0.4 and further incubated in an orbital shaker at 37 °C until an OD_600_ of 1.2 was reached. The developed and dried TLC layers were dipped into the prepared bacterial cell suspension (OD_600_ = 1.2) for 8 s and incubated at 37 °C in a vapor chamber (100% humidity) for 2 h. The visualization of the bioautograms was carried out by dipping them into an aqueous MTT solution (1 mg/mL), followed by an additional 30 min of incubation. The living and metabolically active bacterial cells can reduce the yellow MTT to the purple MTT-formazan; therefore, the white zones against a purple background indicate the presence of antibacterial compounds.

### 2.4 Fractionation and isolation of antibacterial compounds

Fractionation and isolation of antibacterial compounds from the concentrated crude extracts of the petioles were performed by flash column chromatography (CombiFlash NextGen 300, Teledyne Isco, Lincoln, NE, USA) using the following columns: silica gel columns (RediSep Bronze, 40–60 μm, 40 g; RediSep Bronze, 40–60 μm, 12 g; RediSep Bronze, 40–60 μm, 4 g, Teledyne Isco) and FlashPure EcoFlex C18 column (50 μm, 12 g; Büchi, Uster, Switzerland).

The concentrated crude extracts of samples H18 and H19 were separately dried onto preparative silica gel (15 g), placed into a dry-load column, and subjected to flash column chromatography. The fractionation was carried out on a silica gel column (RediSep Bronze, 40–60 μm, 40 g) using a gradient system of *n*-hexane and acetone (0.0–1.0 min, 0%; 1.0–16.0 min, 0–20%; 16.0–26.0 min, 20%; 26.0–36.0 min, 20–100%; 36.0–38.0 min, 100% acetone) at a flow rate of 30 mL/min. This yielded fractions 32–35 (*t*_R_ = 16.2–18.3 min, 118 mg), 36–39 (*t*_R_ = 18.3–21.4 min, 160 mg) and 40–54 (*t*_R_ = 21.4–29.8 min, 66 mg) from sample H18 and fractions 30–33 (*t*_R_ = 16.3–18.5 min, 149 mg), 34–38 (*t*_R_ = 18.5–21.4 min, 125 mg) and 39–52 (*t*_R_ = 21.4–29.5 min, 74 mg) from sample H19. The fractions H18/32–35 were merged with H19/30–33, and H18/40–54 were pooled with H19/39–52. The combined fractions were separately dried, re-suspended in chloroform, and further fractionated on a silica gel column (RediSep Bronze, 40–60 μm, 12 g) using a gradient system of *n*-hexane containing 3% of acetic acid and ethyl acetate (0.0–0.5 min, 0%; 0.5–10.5 min, 0–10%; 10.5–20.5 min, 10%; 20.5–40.5 min, 10–50%; 40.5–44.5 min, 100% ethyl acetate) at a flow rate of 15 mL/min, providing fractions 16–20 (*t*_R_ = 12.3–16.4 min, 81.8 mg) and fractions 59–66 (*t*_R_ = 29.2–33.4 min, 7.4 mg). Fractions 16–20 were dried, re-suspended in chloroform, and further fractionated on a C_18_ column (FlashPure EcoFlex C18, 50 μm, 12 g) using a gradient system of water containing 0.1% formic acid and isopropyl alcohol (0.0–0.5 min, 0%; 0.5–10.5 min, 0–50%; 10.5–15.5 min, 50%; 15.5–19.5 min, 100% isopropyl alcohol) at a flow rate of 10 mL/min to give fractions 14–23 (*t*_R_ = 9.6–14.7 min, 23.9 mg). The fractions 14–23 were dried, re-suspended in chloroform and further purified on a silica gel column (RediSep Bronze, 40–60 μm, 4 g) using a gradient system of chloroform and ethyl acetate (0.0–1.0 min, 0%; 1.0–6.0 min, 0–10%; 6.0–11.0 min, 10%; 11.0–16.0 min, 10–100%; 16.0–18.0 min, 100% ethyl acetate) at a flow rate of 10 mL/min to obtain compound **1** (fractions 19–21, *t*_R_ = 12.7–15.2 min, 14.4 mg).

The fractions 59–66 from the second fractionation were combined, re-suspended in chloroform and further purified on a silica gel column (RediSep Bronze, 40–60 μm, 4 g) using a gradient system of chloroform and isopropyl alcohol (0.0–1.0 min, 0%; 1.0–6.0 min, 0–10%; 6.0–11.0 min, 10%; 11.0–16.0 min, 10–100%; 16.0–18.0 min, 100% isopropyl alcohol) at a flow rate of 10 mL/min to furnish compound **2** (fractions 15–18, *t*_R_ = 9.1–12.3 min, 2.5 mg) and compound **3** (fractions 11–12, *t*_R_ = 6.7–7.5 min, 1.6 mg), respectively. The isolation of compounds **4** (5.6 mg) and **5** (4.3 mg) from the methanol extract of *A. altissima* stem bark has been described previously (Cselőtey et al., 2024).

The purity of all isolated compounds (**1**–**5**) was assessed by ^1^H NMR spectroscopy based on the relative integrals of the ^1^H resonances corresponding to the target compound and to any detectable impurities.

### 2.5 HRMS/MS analysis

Flow injection analysis (FIA)–HRMS/MS and HPLC–HRMS/MS analyses were performed using the combination of a UHPLC system (Vanquish Flex VF-P10, Dionex Softron, Germering, Germany) and a hybrid quadrupole-Orbitrap mass spectrometer equipped with an HESI-II probe (Orbitrap Exploris 120, Thermo Fisher Scientific, Bremen, Germany). For TLC–HRMS, a TLC-MS Interface 2 with an oval elution head (4 mm × 2 mm, CAMAG) was installed between the UHPLC system and the Orbitrap mass spectrometer. For FIA–HRMS and TLC–HRMS, methanol was used as a mobile phase at a flow rate of 0.2 mL/min. HRMS spectra were recorded in both negative and positive ionization modes as full scan in the range of *m/z* 100–1000 with a resolution of 120,000, using spray voltages +3.4 kV and −3.0 kV, respectively. The automatic gain control (AGC) target was set to standard, and the maximum injection time (IT) was 100 ms. Ion transfer tube temperature was maintained at 320 °C, while the vaporizer temperature was 250 °C, and nitrogen was used as both sheath and auxiliary gas (10 and 5 arbitrary units, respectively), produced by a Peak Scientific gas generator (Genius XE 35, Glasgow, United Kingdom). Tandem mass (HRMS/MS) spectra were acquired in parallel reaction monitoring (PRM) mode with a mass isolation of the target molecule, in HCD fragmentation mode using normalized collision energies ranging from 10% to 70%, a quadrupole isolation window of *m*/*z* 0.4 for precursor ion selection, an MS^2^ resolution of 120,000 without lock mass correction, and a maximum IT of 100 ms.

HPLC separation of hydroxylated, polyunsaturated fatty acids (9*Z*,11*E*)-13-hydroxy-9,11-octadecadienoic acid (13-HODE, **4**) and (10*E*,12*Z*)-9-hydroxy-10,12-octadecadienoic acid (9-HODE, **5**), previously isolated from *A. altissima* stem bark, was carried out at 35 °C on a Reprospher 100 C18-DE column (150 mm × 3 mm ID, 5 μm particle size, Dr. Maisch, Ammerbuch, Germany) using a mobile phase flow rate of 0.6 mL/min. The gradient of water containing 0.1% formic acid (A) and methanol containing 0.1% formic acid (B) was as follows: 0–12 min, 77% B; 12–15 min, 77–100% B; 15–18 min, 100% B and 18.1–20 min, 77% B. The analytes were detected in negative ESI ionization mode using a spray voltage of −2.0 kV. The ion transfer tube temperature was maintained at 320 °C, the vaporizer temperature was 250 °C, and nitrogen was used as both the sheath and auxiliary gas (10 and 5 arbitrary units, respectively). Tandem mass (HRMS/MS) spectra were acquired in PRM mode with a mass isolation of the target molecule, in HCD fragmentation mode using normalized collision energies of 25%, a quadrupole isolation window of *m/z* 0.4 for precursor ion selection, an MS^2^ resolution of 120,000, and a maximum IT of 100 ms. Instrument control, operation, and data processing were performed with Xcalibur 4.7.69 software (Thermo Fisher Scientific).

### 2.6 UV spectroscopy

UV spectra were recorded at room temperature using a Lambda 35 spectrophotometer (PerkinElmer, Waltham, MA, USA). Measurements were performed in the wavelength range of 190–400 nm with a scan speed of 60 nm/min and a slit width of 1 nm. Spectra were processed and analyzed by the UV WinLab software (PerkinElmer, version 5.2.0.0646).

### 2.7 ATR-FTIR spectroscopy

ATR-FTIR spectra were obtained using a PerkinElmer Spectrum 400 FT-IR/FT-NIR instrument equipped with a diamond/ZnSe ATR crystal and an MIR TGS detector. Spectra were collected in the range of 4000–650 cm^−1^ with a spectral resolution of 4 cm^−1^ and 32 scans per sample. Data were processed and analyzed by PerkinElmer Spectrum Software (version 6.3.1).

### 2.8 Polarimetry

Optical rotations were measured in chloroform at 25 °C using a PerkinElmer 341 LC polarimeter with a thermostated cell (optical path length: 1 dm), employing the sodium D-line (589.3 nm).

### 2.9 NMR spectroscopy

The isolated compounds were dissolved in chloroform-*d* (CDCl_3_) and transferred to standard 5-mm NMR tubes for analysis. All NMR spectra were acquired on a Bruker AVANCE NEO 600 spectrometer (^1^H: 600.18 MHz, ^13^C: 150.93 MHz; 14.1 T) equipped with a 5-mm quadruple-resonance, *z*-gradient, cryogenically cooled probe (CP 2.1 QCI 600S3 H&F-P/C/N-D-05 Z XT) (Bruker Corporation, Billerica, MA, USA) at 298 K. Instrument operation, control, and data acquisition were performed by Bruker TopSpin 4.0.8 software (Bruker Corporation). ^1^H and ^13^C NMR chemical shifts (*δ*) are reported on the delta scale as parts per million (ppm) referenced to the NMR solvent used (CHCl_3_ residual peak at *δ*_H_ = 7.26 ppm and CDCl_3_ at *δ*_C_ = 77.16 ppm). Spin-spin coupling constants (*J*) are given in Hz. The signal multiplicities are denoted as follows: s – singlet, t – triplet, m – multiplet, dd – doublet of doublets, dq – doublet of quartets, ddd – doublet of doublet of doublets. The multiplicity of overlapped resonances could not be determined and was therefore not reported. The complete ^1^H and ^13^C resonance assignments were achieved using standard one-dimensional ^1^H (*zg30*) and ^13^C DEPTQ (*deptqgpsp*) experiments, along with two-dimensional homonuclear ^1^H–^1^H COSY (*cosygpmfppqf*), ^1^H–^1^H TOCSY (*dipsi2gpphzs*, mixing time: 80 ms), ^1^H–^1^H ROESY (*roesyphpp.2*, mixing time: 200 ms), and heteronuclear ^1^H–^13^C multiplicity-edited HSQC (*hsqcedetgpsp.3*, ^1^*J*_C–H_ = 145 Hz) and ^1^H–^13^C HMBC (*hmbcetgpl3nd*, ^n^*J*_C–H_ = 8 Hz) experiments. In case of compounds **2** and **3**, 2D band-selective ^1^H–^13^C HSQC (*shsqcetgpsisp2.2*, ^1^*J*_C–H_ = 145 Hz) and 2D band-selective HMBC (*shmbcctetgpl2nd*, ^n^*J*_C–H_ = 8 Hz) spectra were also recorded to enhance the spectral resolution in *F*_1_ dimension. All measurements were carried out using the pulse sequences listed in parentheses above and included in the standard spectrometer software library.

### 2.10 Microplate assays

Determination of the minimum inhibitory concentration (MIC) values of the isolated compounds (**1**–**5**) against the Gram-positive *B. subtilis*, *C. flaccumfaciens* pv. *flaccumfaciens*, *C. michiganensis*, *R. fascians*, and the Gram-negative *D. chrysanthemi*, *P. carotovorum*, *P. atrosepticum*, *X. citri* pv. *malvacearum*, *P. syringae* pv. *tomato*, *P. syringae* pv. *maculicola*, and *A. tumefaciens* was performed using non-treated, flat-bottom 96-well microplates (VWR, Debrecen, Hungary) as previously described in (Baglyas et al., 2025). MIC was defined as the lowest concentration of the tested compound required that inhibited bacterial growth compared with the negative control. MIC values were considered as the lowest repeatable concentrations at which bacterial growth was reduced to below 3% of the negative control, based on OD measurements. In addition, the antifungal activity of the isolated compounds (**1**–**5**) against *F. graminearum* was evaluated, and half-maximal inhibitory concentration (IC_50_) values were determined using non-treated, U-bottom 96-well microplates (Nest Scientific, Woodbridge, NJ, USA; cat. no. 701111). Technical replicates (two or three per concentration) were averaged to generate a single data point per biological replicate. Individual IC_50_ values for each independent experiment were determined by linear interpolation from the regression line fitted to the linear range of the dose–response curve. The final reported IC_50_ values are expressed as the mean ± standard deviation of at least two independent experiments. *B. subtilis* was grown in LB broth at 37 °C by shaking at 120 rpm. *C. flaccumfaciens* pv. *flaccumfaciens* and *C. michiganensis* were cultured in Nutrient Broth (NB; 11 g/L peptones, 5 g/L sodium chloride; Biolab, Budapest, Hungary) at 28 °C by shaking at 120 rpm for 12 h, while *R. fascians* was grown on Waksman agar plate (5 g/L meat extract, 5 g/L peptone (Scharlab), 5 g/L sodium chloride (Reanal), 10 g/L *D*-glucose (Reanal), 15 g/L agar, pH adjusted to 7.2 with a 40% aqueous sodium hydroxide (Reanal) solution) at 30 °C for 24 h. Gram-negative bacterial strains were grown on LB agar plate at 28 °C for 12h. *F. graminearum* was cultured in LB broth at 21 °C for 72 h by shaking at 120 rpm.

Neomycin (0.1 or 1 mg/mL in water) and benomyl (25 mg/mL in ethanol) served as positive controls for the antibacterial and antifungal assays, respectively, while ethanol was used as a negative control. Literature data confirm that neomycin is effective against several plant pathogenic bacteria under both *in vitro* and *in vivo* conditions (Cui et al., 2009; Tao et al., 2011). A two-fold ethanolic dilution series of 10 µL of the isolates dissolved in ethanol (8 mg/mL for compound **1** and 2 mg/mL for compounds **2**–**5**) and positive controls (5 µL per well) was prepared. Following dilution, ethanol was evaporated from the wells in a laminar box (BA-900, Radel & Hahn, Debrecen, Hungary). For bacterial assays, 150 µL of bacterial suspension (10^5^ CFU/mL) was added to each well. For *F. graminearum* tests, 70 µL of LB broth was mixed with 50 µL of a mycelium suspension (OD_600_ = 0.2 in LB broth) in the wells. The absorbance at 600 nm was measured using a microplate reader (Clariostar^®^ Plus, BMG LABTECH, Ortenberg, Germany) immediately and following incubation with shaking at 900 rpm (PHMP Twin microplate shaker-incubator, Grant Inc., Beaver Falls, PA, USA). The incubation time and temperature were for *B. subtilis* 24 h at 37 °C, for *C. flaccumfaciens* pv. *flaccumfaciens* and *C. michiganensis* 48 h at 28 °C, for *R. fascians* 48 h at 30 °C, and for Gram-negative bacterial strains 24 h at 28 °C. Antifungal assays were incubated for 72 h at 21 °C without shaking. All experiments were performed in duplicate or triplicate (two or three technical replicates) and were independently repeated on at least two separate occasions (at least two biological replicates).

## 3 Results and discussion

### 3.1 Detection and isolation of antibacterial compounds

**Figure 1:**
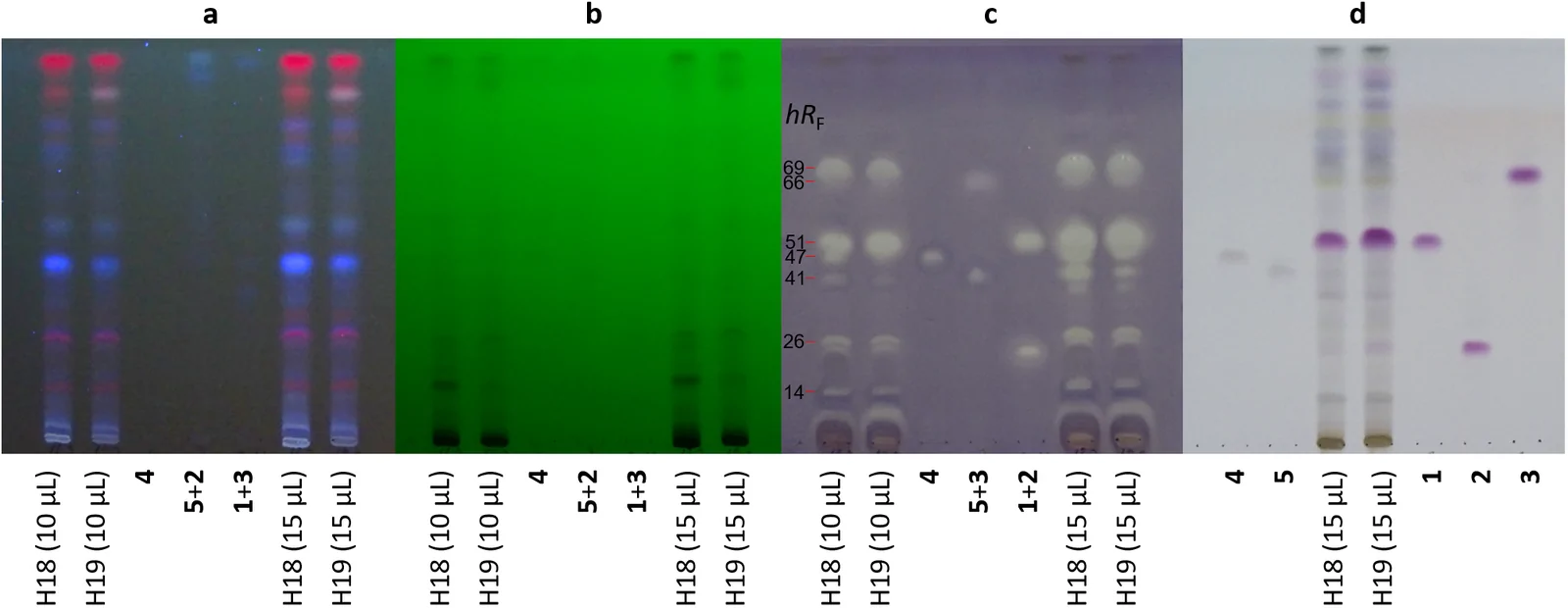
TLC chromatograms **(a, b, d)** and bioautogram **(c)** of the methanolic *Ailanthus altissima* petioles crude extracts (H18 and H19), and their antibacterial isolated components (**1**–**5**) developed with toluene–isopropyl acetate–methanol (5:4:1, *V*/*V*), detected at UV 366 nm **(a)**, 254 nm **(b)**, after *B. subtilis* assay (under white light, **(c)** and derivatization with *p*-anisaldehyde reagent [under white light, **(d)**].

**Figure 2:**
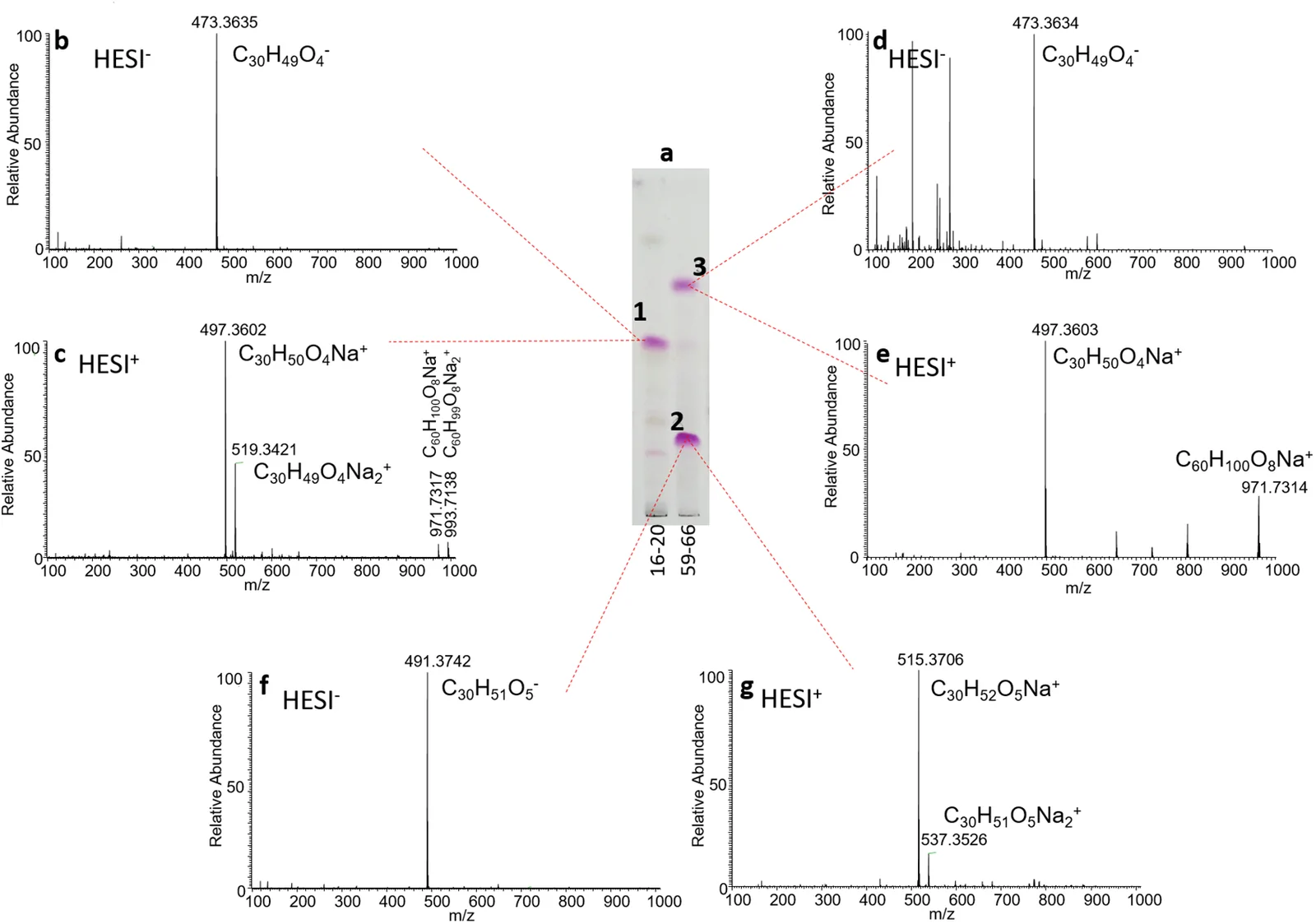
TLC chromatogram of the flash chromatography fractions (fr. 16–20 and 59–66) of the methanolic *Ailanthus altissima* petiole crude extracts (H.18 and H.19) developed with toluene–isopropyl acetate–methanol (5:4:1, *V*/*V*) after derivatization with *p*-anisaldehyde reagent **(a)**, and TLC–HR-HESI^–^MS **(b, d, f)** and TLC–HR-HESI^+^-MS **(c, e, g)** spectra of the bioactive zones of compounds **1**–**3** obtained via the elution head-based TLC-MS interface and background subtraction.

The methanolic extracts of the *A. altissima* petioles were screened by TLC combined with UV detection, derivatization using *p*-anisaldehyde reagent, and a *B. subtilis* bioassay. Similar chemical profiles were observed for samples collected at different locations and time points (**Supplementary Table 1**, **Supplementary Figures 1**, **2**). Compounds in the antibacterial zones revealed in TLC–*B. subtilis* assay (white zones in **Figure 1**; **Supplementary Figures 2c**) were further characterized using TLC hyphenations (UV/FLD, chemical derivatization, HRMS) as well as fractionated and optionally purified by flash column chromatography. In the inhibitory zone at *hR*_F_ 69, a mixture of palmitic acid, linolenic acid, and linoleic acid was identified by comparison with reference standards and TLC–HRMS. The antibacterial activities of these compounds have previously been reported (Móricz et al., 2017). In the zones at *hR*_F_ 41 and 47, a mixture of hydroxylated octadecadienoic acid (HODE, with mass signal at *m/z* 295.2271 [M–H]^–^) and hydroxylated octadecatrienoic acid (HOTrE, with mass signal at *m/z* 293.2113 [M–H]^–^) isomers was detected, which were enriched in the H18/36–39 and H19/34–38 flash chromatographic fractions (**Figures 1** and **S3**). The presence of 13-HODE (compound **4** with characteristic fragment ion at *m/z* 195.1) and 9-HODE (compound **5** with characteristic fragment ion at *m/z* 171.1) in the petiole extracts, which were previously isolated from stem bark (Cselőtey et al., 2024), was confirmed by HPLC–HRMS/MS analysis (**Supplementary Figure 4**). Note that HPLC–HRMS/MS also enabled the tentative identification of 10/12-HODE, 9-HOTrE, 10/12-HOTrE, 13-HOTrE, and 16-HOTrE, with characteristic fragment ions at *m/z* 183.1, *m/z* 171.1, *m/z* 183.1, *m/z* 195.1, and *m/z* 235.2, respectively (Koch et al., 2023). Preparative normal- and reversed-phase flash column chromatographic fractionation process was guided by TLC–bioassay, TLC–*p*-anisaldehyde reagent, and TLC–HRMS, resulting in the isolation of compounds **1** and **3** from the zones at *hR*_F_ 51 and 66, respectively (**Figures 1**, **2**). This approach also enabled the detection of a previously invisible inhibitory zone in the crude extract at *hR*_F_ 24, corresponding to compound **2**, which appeared only after the first fractionation step in fractions H18/40–54 and H19/39-52 (**Figure 2**; **Supplementary Figure S3**).

### 3.2 Structure elucidation

The structures of the isolated compounds (**1**–**3**) were elucidated by comprehensive, in-depth spectroscopic and spectrometric methods and comparison with literature data. The novelty of compounds **2** and **3** was confirmed using CAS SciFinder^®^ and Reaxys databases. The recorded one-dimensional (1D) and two-dimensional (2D) NMR spectra (**Supplementary Figures 7–15** for **1**, **Supplementary Figures 20–29** for **2**, **Supplementary Figures 36–45** for **3**), HR-HESI-MS(/MS) spectra (**Supplementary Figures 16–19** for **1**, **Supplementary Figures 30–33** for **2**, **Supplementary Figures 46–49** for **3**), UV spectra (**Supplementary Figure 34** for **2**, **Supplementary Figure 50** for **3**), and ATR-FTIR spectra (**Supplementary Figure 35** for **2**, **Supplementary Figure 51** for **3**) are provided in the **Supplementary Materials**.

**Figure 3:**
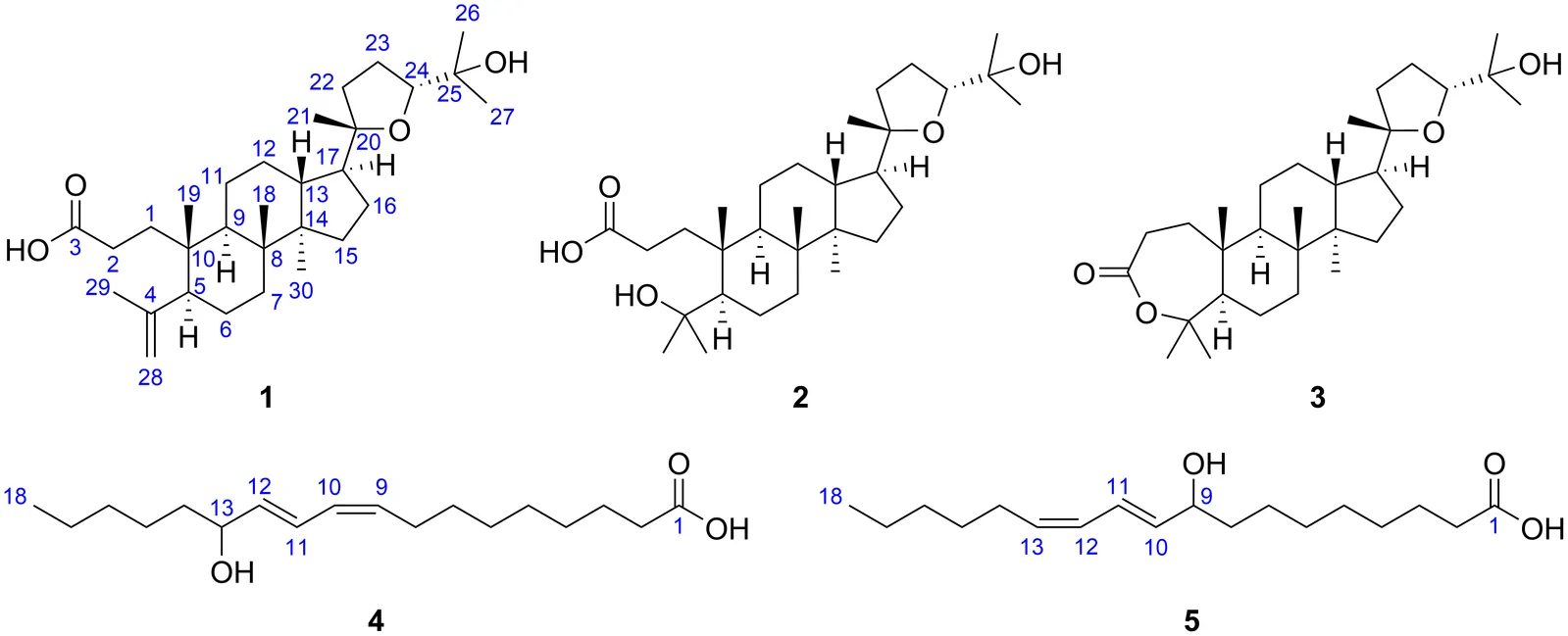
The chemical structures of the isolated compounds (**1**–**5**) with the atomic numbering (blue).

Shoreic acid (**1**) (**Figure 3**) was obtained as a white amorphous solid. Its molecular formula was established as C_30_H_50_O_4_, deduced from the ^13^C DEPTQ NMR spectrum and based on the positive-ion mode (*m*/*z* 497.3602 [M+Na]^+^ (calculated for C_30_H_50_O_4_Na^+^, *m*/*z* 497.3601 [M+Na]^+^, error: +0.1 ppm)) and the negative-ion mode HR-HESI-MS spectra (*m*/*z* 473.3633 [M−H]^−^ (calculated for C_30_H_49_O_4_^−^, *m*/*z* 473.3636 [M−H]^−^, error: −0.7 ppm)), indicating 6 double bond equivalents (DBEs). The ^1^H NMR spectrum of compound **1** (**Table 1**; **Supplementary Table S2**) exhibited proton resonances corresponding to seven methyl groups at *δ*_H_ 1.73 (s, 3H, H_3_-29), 1.21 (s, 3H, H_3_-26), 1.13 (s, 3H, H_3_-21), 1.12 (s, 3H, H_3_-27), 1.00 (s, 3H, H_3_-18), 0.88 (s, 3H, H_3_-30), 0.84 (s, 3H, H_3_-19), a methylidene group at *δ*_H_ 4.84 (t, *J* = 2.0 Hz, 1H, H_2_-28a), 4.66 (m, 1H, H_2_-28b), and an oxymethine group at *δ*_H_ 3.73 (t, *J* = 7.4 Hz, 1H, H-24). Based on ^13^C DEPTQ (**Tables 1**; **Supplementary Table S2**), ^1^H–^13^C multiplicity-edited HSQC (edHSQC), and ^1^H–^13^C HMBC spectroscopic data, the thirty ^13^C NMR resonances were assigned to seven methyl groups (*δ*_C_ 27.5, 24.4, 23.6, 23.3, 20.3, 16.5, 15.4), one vinylic methylene (*δ*_C_ 113.6) and ten aliphatic methylene groups (*δ*_C_ 35.9, 34.4, 34.0, 31.6, 28.4, 27.4, 26.3, 25.8, 24.7, 22.2), one oxygenated methine (*δ*_C_ 83.5) and four aliphatic methine groups (*δ*_C_ 50.9, 49.6, 43.2, 41.3), and seven non-hydrogenated carbons, including one carboxylic (*δ*_C_ 179.8), one olefinic (*δ*_C_ 147.6), two oxygenated (*δ*_C_ 86.6, 71.7), and three aliphatic carbons (*δ*_C_ 50.5, 40.2, 39.2). Thus, the presence of one carboxyl group and one carbon–carbon double bond was revealed, indicating a tetracyclic molecule to account for the required number of DBEs. A total of 48 hydrogen atoms were accounted for, and the remaining two exchangeable hydrogens were assigned to one carboxyl and one hydroxyl group. 2D NMR spectroscopic data along with comparison to previously reported data (Roux et al., 1998; Guo et al., 2023) supported the identification of compound **1** as shoreic acid, a 20,24-epoxy-3,4-secodammarane triterpene with a (20*S*,24*R*) absolute configuration. Note that the stereoisomers eichlerianic acid (20*S*,24*S* isomer) (Lavie et al., 1984) and isoeichlerianic acid (20*R*,24*S* isomer) (Seger et al., 2008) can be distinguished from shoreic acid based on the ^1^H NMR chemical shift and multiplicity of H-24, ^13^C NMR chemical shifts, and ^1^H–^1^H NOE correlations (Seger et al., 2008). Shoreic acid has been reported from several trees, including the leaves of *A. altissima* (Guo et al., 2023), the bark of *Aglaia foveolate* (Roux et al., 1998), and the leaves of *Cabralea canjeranaand* (Leitão et al., 2008). However, to the best of our knowledge, the present study constitutes the first report of the isolation of shoreic acid from the petioles of *A. altissima*.

**Table 1:** ^1^H NMR (600 MHz, CDCl_3_) and ^13^C NMR (151 MHz, CDCl_3_) spectroscopic data for the isolated compounds (**1**–**3**) in chloroform-*d* (*δ* in ppm, *J* in Hz).

Position	Shoreic acid (1)		Foveolin C (2)		(20*S*,24*R*)-epoxy-25-hydroxy-A-homo-4-oxadammaran-3-one (3)	
	*δ*_H_ (ppm), Multiplicity, *J* (Hz)	*δ*_C_ (ppm), Type	*δ*_H_ (ppm), Multiplicity, *J* (Hz)	*δ*_C_ (ppm), Type	*δ*_H_ (ppm), Multiplicity, *J* (Hz)	*δ*_C_ (ppm), Type
1a	1.61^a^	34.4, CH_2_	2.42, m	34.9, CH_2_	1.81^a^	40.5, CH_2_
1b			1.78^a^		1.58^a^	
2a	2.38, ddd (*J* = 15.9, 10.0, 6.4 Hz)	28.4, CH_2_	2.49, m	29.3, CH_2_	2.64, ddd (*J* = 14.3, 12.8, 4.6 Hz)	32.5, CH_2_
2b	2.18, m		2.20, m		2.51, m	
3	–	179.8, C	–	178.3, C	–	175.2, C
4	–	147.6, C	–	76.9, C	–	86.3, C
5	1.97, dd (*J* = 12.7, 3.1 Hz)	50.9, CH	1.42^a^	51.9, CH	1.77, dd (*J* = 12.5, 3.3 Hz)	53.3, CH
6a	1.82^a^	24.7, CH_2_	1.56^a^	22.7, CH_2_	1.69^a^	23.7, CH_2_
6b	1.36, dq (*J* = 13.5, 3.4 Hz)		1.46^a^		1.42^a^	
7a	1.53^a^	34.0, CH_2_	1.50^a^	34.9, CH_2_	1.53^a^	34.4, CH_2_
7b	1.23^a^		1.24^a^		1.29^a^	
8	–	40.2, C	–	40.2, C	–	40.3, C
9	1.51^a^	41.3, CH	1.49^a^	42.6, CH	1.43^a^	51.5, CH
10	–	39.2, C	–	41.4, C	–	39.4, C
11a	1.42, m	22.2, CH_2_	1.52^a^	21.4, CH_2_	1.52^a^	22.9, CH_2_
11b	1.24^a^		1.18^a^		1.28^a^	
12a	1.79^a^	27.4, CH_2_	1.79^a^	27.5, CH_2_	1.80^a^	27.6, CH_2_
12b	1.24^a^		1.24^a^		1.24^a^	
13	1.59^a^	43.2, CH	1.59, m	43.2, CH	1.63^a^	43.4, CH
14	–	50.5, C	–	50.5, C	–	50.1, C
15a	1.46^a^	31.6, CH_2_	1.45^a^	31.6, CH_2_	1.45, m	31.4, CH_2_
15b	1.10, m		1.08, m		1.10^a^	
16a	1.78^a^	25.8, CH_2_	1.78^a^	25.8, CH_2_	1.77^a^	25.7, CH_2_
16b	1.46^a^		1.48^a^		1.49^a^	
17	1.81^a^	49.6, CH	1.81^a^	49.6, CH	1.82^a^	49.5, CH
18	1.00, s	15.4, CH_3_	0.97, s	15.5, CH_3_	1.01, s	15.0, CH_3_
19	0.84, s	20.3, CH_3_	1.01, s	20.8, CH_3_	1.07, s	18.5, CH_3_
20	–	86.6, C	–	86.5, C	–	86.4, C
21	1.13, s	23.6, CH_3_	1.13, s	23.6, CH_3_	1.14, s	23.6, CH_3_
22a	1.70, m	35.9, CH_2_	1.70, m	36.0, CH_2_	1.70^a^	36.0, CH_2_
22b	1.63^a^		1.64, m		1.64^a^	
23a	1.87, m	26.3, CH_2_	1.87, m	26.3, CH_2_	1.87, m	26.3, CH_2_
23b	1.78^a^		1.79^a^		1.79^a^	
24	3.73, t (*J* = 7.4 Hz)	83.5, CH	3.73, t (*J* = 7.4 Hz)	83.5, CH	3.73, t (*J* = 7.4 Hz)	83.5, CH
25	–	71.7, C	–	71.6, C	–	71.6, C
26	1.21^b^, s	27.5^b^, CH_3_	1.21^b^, s	27.6^b^, CH_3_	1.21^b^, s	27.6^b^, CH_3_
27	1.12^b^, s	24.4^b^, CH_3_	1.12^b^, s	24.4^b^, CH_3_	1.12^b^, s	24.4^b^, CH_3_
28a	4.84, t (*J* = 1.9 Hz)	113.6, CH_2_	1.31^c^, s	34.7^c^, CH_3_	1.48^c^, s	31.1^c^, CH_3_
28b	4.66, m					
29	1.73, s	23.3, CH_3_	1.26^c^, s	27.5^c^, CH_3_	1.41^c^, s	27.0^c^, CH_3_
30	0.88, s	16.5, CH_3_	0.86, s	16.4, CH_3_	0.86, s	16.3, CH_3_

^a^ Overlapped signals (multiplicities could not be determined).

^b^ , ^c^Interchangeable resonances.

The multiplicity of the overlapped (ov.) signals could not be determined and was therefore not reported.

**Figure 4:**
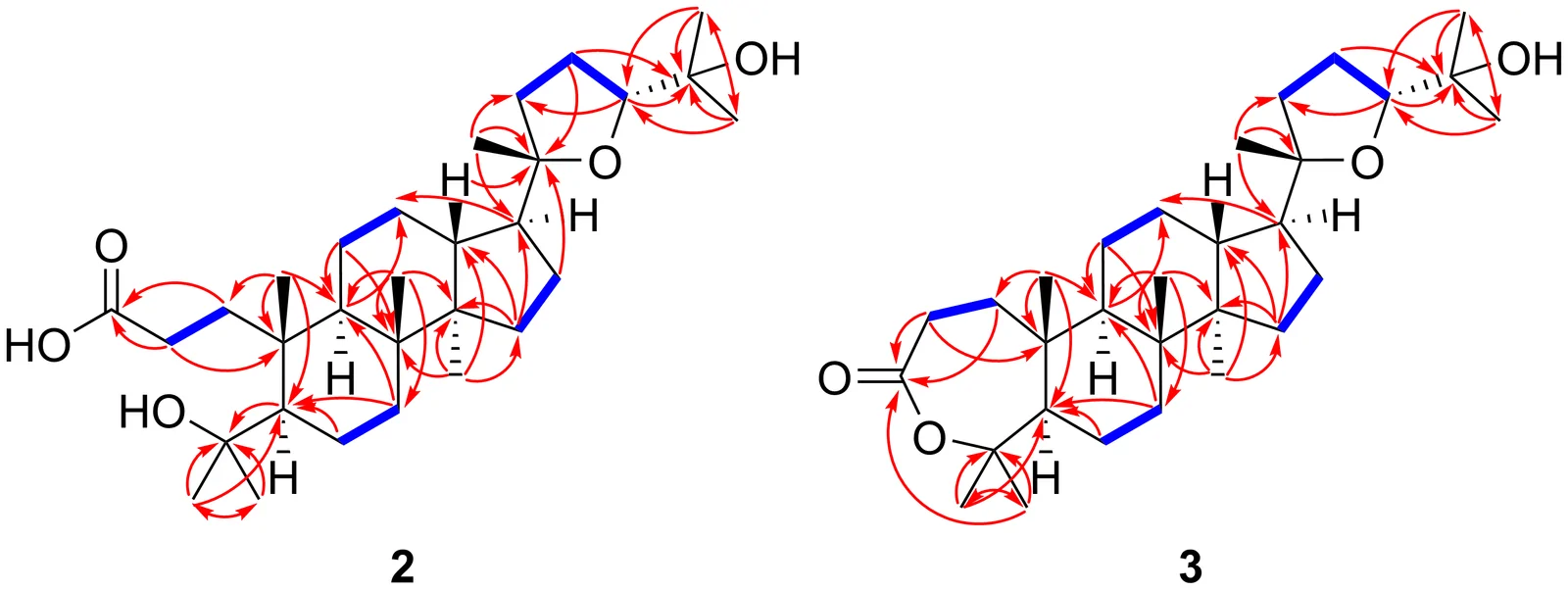
Key ^1^H–^1^H COSY (blue) and ^1^H–^13^C HMBC (red) correlations of foveolin C (**2**) and (20*S*,24*R*)-epoxy-25-hydroxy-A-homo-4-oxadammaran-3-one (**3**).

**Figure 5:**
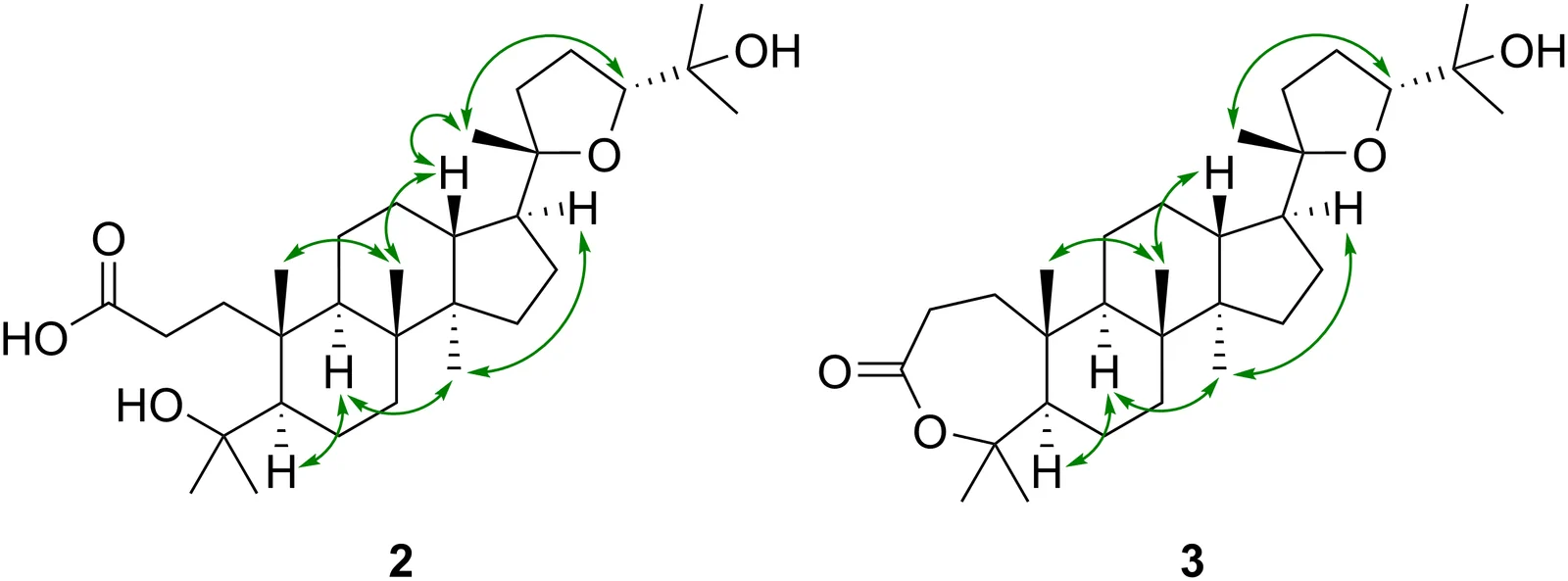
Key ^1^H–^1^H NOE correlations (green) of foveolin C (**2**) and (20*S*,24*R*)-epoxy-25-hydroxy-A-homo-4-oxadammaran-3-one (**3**).

Foveolin C (**2**) (**Figure 3**) was obtained as a white amorphous solid with a specific optical rotation of [*α*]_D_^25^ +19.3 (*c* 0.25, CHCl_3_). It gave a molecular formula of C_30_H_52_O_5_ based on the ^13^C DEPTQ NMR data and the positive-ion HR-HESI-MS signal at *m*/*z* 515.3706 [M+Na]^+^ (calculated for C_30_H_52_O_5_Na^+^, *m*/*z* 515.3707 [M+Na]^+^, error: −0.2 ppm) and at *m*/*z* 491.3739 [M−H]^−^ (calculated for C_30_H_51_O_5_^−^, *m*/*z* 491.3742 [M−H]^−^, error: −0.6 ppm), demanding 6 DBEs. The ^1^H and ^13^C NMR spectroscopic data (**Table 1**) demonstrated that compound **2** shared the same 20,24-epoxy-3,4-secodammarane skeleton of shoreic acid (**1**), except for the presence of a tertiary alcohol [C(OH)(CH_3_)_2_] moiety at C-5 in **2** replacing the terminal isopropenyl group in **1**. This was indicated by the C–OH resonance at *δ*_C_ 76.9 (C-4) and signals at *δ*_H_ 1.31 (s, 3H, H_3_-28) and *δ*_C_ 34.7 (C-28) corresponding to an additional tertiary methyl group. ^1^H-^13^C HMBC correlations (**Figure 4**) from H_3_-28 to C-29 and from H_3_-29 to C-28, together with the fact that H_3_-28 and H_3_-29 shared all other correlations (to C-4 and C-5), confirmed the presence of two geminal-related methyl groups. The attachment of C(OH)(CH_3_)_2_ moiety to C-5 was confirmed by the HMBC correlations from H_3_-28 to C-5, from H_3_-29 to C-5, and from H-5 to C-4. The intense, sharp IR absorption band at 1708 cm^−1^ (C=O stretching vibration) is indicative of a carboxyl moiety. The relative configuration of **2** was deduced to be the same as that of **1** by comparing their ^1^H and ^13^C NMR chemical shifts (**Table 1**) and ^1^H-^1^H NOE interactions, including the key NOE enhancements between H-5/H-9, H-9/H_3_-30, H_3_-30/H-17 for *α*-oriented hydrogens and between H_3_-19/H_3_-18, H_3_-18/H-13, H-13/H_3_-21 for *β*-oriented hydrogens (**Figure 5**). Foveolin A (20*S*,24*S*-isomer, **2a**, **Supplementary Figure 5**) and foveolin B (20*R*,24*S*-isomer, **2b**, **Supplementary Figure 5**) possess the same planar structure as compound **2**; however, key differences were revealed in their NMR data. In compounds **2** and **2b** H-24 resonated as a triplet (*δ*_H_ 3.73 (t, *J* = 7.4 Hz) for **2** and *δ*_H_ 3.73 (t, *J* = 7 Hz) for **2b**), whereas in compound **2a** it appeared as a doublet of doublets [*δ*_H_ 3.62 (dd, *J* = 10, 5.5 Hz)] (**Supplementary Table 3**) (Roux et al., 1998). In addition, significant ^13^C NMR chemical shift differences were observed at C-21 (*δ*_C_ 23.6 for **2**, 27.0 for **2a**, and 22.0 for **2b**), at C-22 (*δ*_C_ 36.0 for **2**, 34.8 for **2a**, and 37.5 for **2b**), at C-24 (*δ*_C_ 83.5 for **2**, 86.3 for **2a**, and 84.5 for **2b**), and at C-25 (*δ*_C_ 71.6 for **2**, 70.3 for **2a**, and 71.6 for **2b**) (**Supplementary Table 4**) (Roux et al., 1998; Seger et al., 2008). The ^13^C NMR chemical shifts of compound **2** in the stereochemically variable region of the tetrahydrofuran (20,24-epoxy) ring closely matched those of compound **1** and differed significantly from those of foveolins A and B, supporting the assignment of compound **2** to the same stereochemical series as compound **1**. ^1^H–^1^H ROESY spectrum featured a strong cross peak between H_3_-21/H-24 (**Figure 5**), corroborating their co-facial orientation on the ring, and between H-13/H_3_-21 (**Figure 5**), confirming the (20*S*,24*R*) configuration. Compound **2** was consequently identified as a previously undescribed 20,24-epoxy-3,4-secodammarane triterpene, representing the (20*S*,24*R*) diastereomer relative to foveolins A and B, and was accordingly named foveolin C. Foveolins A and B were firstly isolated from the bark of *A. foveolate*, and their stereochemistry was originally assigned as (20*S*,24*S*) and (20*S*,24*R*), respectively, by Roux et al (Roux et al., 1998). However, it was subsequently demonstrated that the absolute configuration of foveolin B is not identical to that of shoreic acid [(20*S*,24*R*)], and its configuration was revised to (20*R*,24*S*) (Seger et al., 2008). The present work further supports and is fully consistent with this stereochemical revision.

(20*S*,24*R*)-Epoxy-25-hydroxy-A-homo-4-oxadammaran-3-one (**3**) (**Figure 3**) was obtained as a white amorphous solid with a specific optical rotation of [*α*]_D_^25^ +40.8 (*c* 0.085, CHCl_3_). Its molecular formula was determined to be C_30_H_50_O_4_, the same as that of compound **1**, deduced from the ^13^C DEPTQ NMR and positive- and negative-ion mode HR-HESI-MS spectra at *m*/*z* 497.3603 [M+Na]^+^ (calculated for C_30_H_50_O_4_Na^+^, *m*/*z* 497.3601 [M+Na]^+^, error: +0.3 ppm) and *m*/*z* 473.3634 [M−H]^−^ (calculated for C_30_H_49_O_4_^−^, *m*/*z* 473.3636 [M−H]^−^, error: −0.6 ppm), demanding 6 DBEs. The ^1^H and ^13^C NMR spectroscopic data (**Table 1**) revealed that compound **3** is a structural isomer of compound **1** and showed similar structural pattern as that of compound **2**. However, notable differences were observed in the ^1^H NMR chemical shifts of H_2_-1 (*δ*_H_ 1.81, 1.58 in **3**; 2.42, 1.78 in **2**), H_2_-2 (*δ*_H_ 2.64, 2.51 in **3**; 2.49, 2.20 in **2**), H_3_-28 (*δ*_H_ 1.48 in **3**, 1.31 in **2**), H_3_-29 (*δ*_H_ 1.41 in **3**, 1.26 in **2**), H-5 (*δ*_H_ 1.77 in **3**, 1.42 in **2**). Furthermore, corresponding differences were also evident in the ^13^C NMR chemical shifts of C-3 (*δ*_C_ 175.2 in **3**, 178.3 in **2**; Δ*δ*_C_ = −3.1 ppm), C-4 (*δ*_C_ 86.3 in **3**, 76.9 in **2**; Δ*δ*_C_ = +9.4 ppm), C-1 (*δ*_C_ 40.5 in **3**, 34.9 in **2**; Δ*δ*_C_ = +5.6 ppm), C-2 (*δ*_C_ 32.5 in **3**, 29.3 in **2**; Δ*δ*_C_ = +3.2 ppm), and C-28 (*δ*_C_ 31.1 in **3**, 34.7 in **2**; Δ*δ*_C_ = −3.6 ppm). In addition, the loss of H_2_O unit between the molecular formulae of compounds **2** (C_30_H_52_O_5_) and **3** (C_30_H_50_O_4_) proposed the presence of an ester bond between the carbonyl group of C-3 and the quaternary oxygenated carbon of C-4, resulting in the closure of the 3,4-seco A ring to form a seven-membered lactone. The *ϵ*-lactone ring formation was confirmed by the key HMBC correlations from H_3_-29 to C-3, from H_2_-1 to C-3, and from H_2_-2 to C-3 (**Figure 4**) and corroborated by an intense, sharp IR absorption band at 1728 cm^−1^, corresponding to a 20 cm^−1^ shift to higher wavenumbers relative to compound **2**. The relative configuration of compound **3** was determined to be identical to that of compounds **1** and **2** based on the comparison of their ^1^H–^1^H NOE interactions (**Figure 5**) and ^1^H and ^13^C NMR chemical shifts (**Table 1**), indicating the 20*S*, 24*R* configuration of the epoxy group. (20*S*,24*S*)-epoxy-25-hydroxy-A-homo-4-oxadammaran-3-one (**3a**, **Supplementary Figure 6**) and (20*R*,24*S*)-epoxy-25-hydroxy-A-homo-4-oxadammaran-3-one (**3b**, **Supplementary Figure 6**) was isolated from the stem bark of *A. foveolate* (Pan et al., 2010) and both possess the same planar structure as compound **3**. Comparison of their NMR data with those of compound **3** demonstrated slight but distinctive differences in the tetrahydrofuran ring signals. In particular, H-24 resonated as a triplet in compounds **3** and **3b** (*δ*_H_ 3.73 (t, *J* = 7.4 Hz) for **3** and *δ*_H_ 3.77 (t, *J* = 7.2 Hz) for **3b**), whereas the resonance of H-24 was observed as a doublet of doublets in compound **3a** [*δ*_H_ 3.65 (dd, *J* = 9.9, 5.6 Hz)] (**Supplementary Table 5**) (Pan et al., 2010). Furthermore,^13^C NMR chemical shift differences were observed at C-21 (*δ*_C_ 23.6 for **3**, 27.2 for **3a**, and 22.1 for **3b**), at C-22 (*δ*_C_ 36.0 for **3**, 34.8 for **3a**, and 37.8 for **3b**), at C-24 (*δ*_C_ 83.5 for **3**, 86.4 for **3a**, and 84.9 for **3b**), and at C-25 (*δ*_C_ 71.6 for **3**, 70.2 for **3a**, and 71.5 for **3b**) (**Supplementary Table 6**) (Pan et al., 2010). ^1^H–^1^H ROESY spectrum displayed a strong cross peak between H_3–_21 and H-24 (**Figure 5**), supporting their co-facial orientation on the tetrahydrofuran ring, and between H-13/H_3_-21 (**Figure 5**), confirming the (20*S*,24*R*) configuration. Thus, compound **3** was therefore elucidated as a previously undescribed 20,24-epoxy-3,4-secodammarane triterpene, (20*S*,24*R*)-epoxy-25-hydroxy-A-homo-4-oxadammaran-3-one, the (20*S*,24*R*) isomer of its known diastereomers.

Compounds **4** and **5** (**Figure 3**) were identified in our previous work (Cselőtey et al., 2024) as the monohydroxylated, polyunsaturated fatty acids (9*Z*,11*E*)-13-hydroxy-9,11-octadecadienoic acid (13-HODE; **4**) and (10*E*,12*Z*)-9-hydroxy-10,12-octadecadienoic acid (9-HODE; **5**), respectively. Their structures were confirmed by comparison of their NMR spectroscopic data (Trapp et al., 2015) and their characteristic MS/MS fragmentation patterns (Koch et al., 2023) with those reported in the literature. The purity of all isolated compounds (≥95% for **1**, ~90% for **2**, ~75% for **3**, **4**, and **5**), as assessed by ^1^H NMR spectroscopy, was considered sufficient for the *in vitro* antimicrobial bioassays.

### 3.3 Compound characterization

Shoreic acid (**1**): White amorphous solid; ^1^H (600 MHz, CDCl_3_) and ^13^C (151 MHz, CDCl_3_) NMR spectroscopic data, see **Table 1**; HR-HESI-MS *m*/*z* 497.3602 [M+Na]^+^ (calculated for C_30_H_50_O_4_Na^+^, *m*/*z* 497.3601 [M+Na]^+^, error: +0.1 ppm), *m*/*z* 473.3633 [M−H]^−^ (calculated for C_30_H_49_O_4_^−^, *m*/*z* 473.3636 [M−H]^−^, error: −0.7 ppm); TLC (silica gel): *h*R_F_ 51 (toluene–isopropyl acetate–methanol 5:4:1, *V*/*V*); color after derivatization with *p*-anisaldehyde reagent under white light: pink.

Foveolin C (**2**): White amorphous solid; [*α*]_D_^25^ +19.3 (*c* 0.25, CHCl_3_); UV (EtOH) *λ*_max_ (log *ϵ*) 204 nm (3.46); IR (ATR) *ν*_max_ 3415, 2961, 2930, 2872, 1708, 1459, 1377, 1289, 1265, 1207, 1180, 1114, 1082 cm^−1^; ^1^H (600 MHz, CDCl_3_) and ^13^C (151 MHz, CDCl_3_) NMR spectroscopic data, see **Table 1**; HR-HESI-MS *m*/*z* 515.3706 [M+Na]^+^ (calculated for C_30_H_52_O_5_Na^+^, *m*/*z* 515.3707 [M+Na]^+^, error: −0.2 ppm), *m*/*z* 491.3739 [M−H]^−^ (calculated for C_30_H_51_O_5_^−^, *m*/*z* 491.3742 [M−H]^−^, error: −0.6 ppm); TLC (silica gel): *h*R_F_ 24 (toluene–isopropyl acetate–methanol 5:4:1, *V*/*V*); color after derivatization with *p*-anisaldehyde reagent under white light: pink.

(20*S*,24*R*)-Epoxy-25-hydroxy-A-homo-4-oxadammaran-3-one (**3**): White amorphous solid; [*α*]_D_^25^ +40.8 (*c* 0.085, CHCl_3_); UV (EtOH) *λ*_max_ (log *ϵ*) 205 nm (3.37); IR (ATR) *ν*_max_ 3442, 2962, 2930, 2871, 1728, 1459, 1375, 1329, 1284, 1246, 1211, 1183, 1140, 1112, 1071, 1026 cm^−1^; ^1^H (600 MHz, CDCl_3_) and ^13^C (151 MHz, CDCl_3_) NMR spectroscopic data, see **Table 1**; HR-HESI-MS *m*/*z* 497.3603 [M+Na]^+^ (calculated for C_30_H_50_O_4_Na^+^, *m*/*z* 497.3601 [M+Na]^+^, error: +0.3 ppm), *m*/*z* 473.3634 [M−H]^−^ (calculated for C_30_H_49_O_4_^−^, *m*/*z* 473.3636 [M−H]^−^, error: −0.6 ppm); TLC (silica gel): *h*R_F_ 66 (toluene–isopropyl acetate–methanol 5:4:1, *V*/*V*); color after derivatization with *p*-anisaldehyde reagent under white light: pink.

(9*Z*,11*E*)-13-Hydroxy-9,11-octadecadienoic acid (**4**): White amorphous solid; ^1^H NMR (600 MHz, CDCl_3_) *δ* 6.49 (ddd, *J* = 15.2, 11.1, 1.2 Hz, 1H, H-11), 5.97 (t, *J* = 10.9 Hz, 1H, H-10), 5.66 (dd, *J* = 15.2, 6.7 Hz, 1H, H-12), 5.44 (dt, *J* = 10.9, 7.7 Hz, 1H, H-9), 4.18 (q, *J* = 6.6 Hz, 1H, H-13), 2.34 (t, *J* = 7.4 Hz, 2H, H-2), 2.17 (m, 2H, H-8), 1.63 (p, *J* = 7.3 Hz, 2H, H-3), 1.54 (m, 2H, H-14), 1.38 (p, *J* = 6.6 Hz, 2H, H-7), 1.31 (m, 12H, H-4–H-6, H-15–H-17), 0.89 (t, *J* = 6.7 Hz, 3H, H-18); ^13^C NMR (151 MHz, CDCl_3_) *δ* 178.0 (C, C-1), 135.9 (CH, C-12), 133.0 (CH, C-9), 128.0 (CH, C-10), 125.9 (CH, C-11), 73.1 (CH, C-13), 37.4 (CH_2_, C-14), 33.8 (CH_2_, C-2), 31.9 (CH_2_, C-16), 29.4 (CH_2_, C-7), 28.9 (CH_2_, C-4–C-6), 27.7 (CH_2_, C-8), 25.3 (CH_2_, C-15), 24.8 (CH_2_, C-3), 22.7 (CH_2_, C-17), 14.2 (CH_3_, C-18); HR-HESI-MS *m*/*z* 319.2243 [M+Na]^+^ (calculated for C_18_H_32_O_3_Na^+^, *m*/*z* 319.2244 [M+Na]^+^, error: −0.3 ppm), *m*/*z* 295.2277 [M−H]^−^ (calculated for C_18_H_31_O_3_^−^, *m*/*z* 295.2279 [M−H]^−^, error: −0.7 ppm); TLC (silica gel): *h*R_F_ 47 (toluene–isopropyl acetate–methanol 5:4:1, *V*/*V*); color after derivatization with *p*-anisaldehyde reagent under white light: pink.

(10*E*,12*Z*)-9-Hydroxy-10,12-octadecadienoic acid (**5**): White amorphous solid; ^1^H NMR (600 MHz, CDCl_3_) *δ* 6.48 (ddt, *J* = 15.2, 11.0, 1.1 Hz, 1H, H-11), 5.97 (tt, *J* = 11.1, 1.8 Hz, 1H, H-12), 5.66 (dd, *J* = 15.1, 6.9 Hz, 1H, H-10), 5.45 (dt, *J* = 10.9, 7.6 Hz, 1H, H-13), 4.15 (qd, *J* = 6.3, 2.4 Hz, 1H, H-9), 2.34 (t, *J* = 7.5 Hz, 2H, H-2), 2.18 (m, 2H, H-14), 1.63 (p, *J* = 7.4 Hz, 2H, H-3), 1.54 (m, 2H, H-8), 1.38 (p, *J* = 7.3 Hz, 2H, H-15), 1.30 (m, 12H, H-4–H-7, H-16, H-17), 0.89 (t, *J* = 6.7 Hz, 3H, H-18); ^13^C NMR (151 MHz, CDCl_3_) *δ* 178.2 (C, C-1), 135.8 (CH, C-10), 133.3 (CH, C-13), 127.8 (CH, C-12), 126.1 (CH, C-11), 73.1 (CH, C-9), 37.4 (CH_2_, C-8), 33.9 (CH_2_, C-2), 31.6 (CH_2_, C-16), 29.5, 29.4, 29.3 (CH_2_, C-5, C-6, C-15), 29.1 (CH_2_, C-4), 27.9 (CH_2_, C-14), 25.5 (CH_2_, C-7, C-15), 24.8 (CH_2_, C-3), 22.7 (CH_2_, C-17), 14.2 (CH_3_, C-18); HR-HESI-MS *m*/*z* 319.2243 [M+Na]^+^ (calculated for C_18_H_32_O_3_Na^+^, *m*/*z* 319.2244 [M+Na]^+^, error: −0.1 ppm), *m*/*z* 295.2277 [M−H]^−^ (calculated for C_18_H_31_O_3_^−^, *m*/*z* 295.2279 [M−H]^−^, error: −0.7 ppm); TLC (silica gel): *h*R_F_ 41 (toluene–isopropyl acetate–methanol 5:4:1, *V*/*V*); color after derivatization with *p*-anisaldehyde reagent under white light: pink.

### 3.4 Antimicrobial activity of isolated compounds

The antibacterial and antifungal efficacy of compounds **1**–**5** was assessed by an *in vitro* microdilution assay against the Gram-positive, non-pathogenic soil bacterium *B. subtilis* and the Gram-positive, plant pathogenic bacteria *C. flaccumfaciens* pv. *flaccumfaciens*, *C. michiganensis*, and *R. fascians*, as well as the fungal plant pathogen *F. graminearum*. All isolated compounds showed varying degrees of inhibitory activity against certain tested bacteria, with MIC values ranging from 1.0 to 133.3 μg/mL (**Table 2**). The strongest antibacterial effect of the isolates was observed for compound **1**, with MIC values two-fold lower than those of the positive control neomycin against *C. michiganensis* and *R. fascians*, and MIC values equal to or slightly higher than those of the positive control against *C. flaccumfaciens* pv. *flaccumfaciens* and *B. subtilis*. Compound **1** also displayed the highest antifungal activity among the isolated compounds against *F. graminearum*, with an IC_50_ value approximately 3 times higher than that of the known fungicide benomyl (**Table 2**). Compound **2** demonstrated notable antibacterial and antifungal activity, especially against *R. fascians* and *C. michiganensis*, with MIC values approximately 2 and 8 times higher than those of neomycin, respectively. Compounds **3**, **4**, and **5** exhibited weak to moderate antimicrobial activity overall; however, all three exerted a more pronounced antibacterial effect against *R. fascians*, with MIC values equal to or two-fold higher than that of the positive control.

**Table 2:** The minimum inhibitory concentration (MIC) values for bacterial strains and half-maximal inhibitory concentration (IC_50_) values (mean ± standard deviation) for the fungal pathogen, both expressed in µg/mL, of the isolated compounds (**1**–**5**), and the positive controls neomycin and benomyl, determined against the Gram-positive bacterial strains *Bacillus subtilis*, *Curtobacterium flaccumfaciens* pv. *flaccumfaciens*, *Clavibacter michiganensis*, and *Rhodococcus fascians*, as well as the fungal pathogen *Fusarium graminearum*.

	*Bacillus subtilis*	*Curtobacterium flaccumfaciens* pv*. flaccumfaciens*	*Clavibacter michiganensis*	*Rhodococcus fascians*	*Fusarium graminearum*
Compound	MIC (µg/mL)				IC_50_ (µg/mL)
**1**	2.1	1.0	1.0	2.1	13.2 ± 2.9
**2**	33.3	33.3	16.7	8.3	85.4 ± 5.3
**3**	>133.3^*^	>133.3	33.3	8.3	>166.7
**4**	66.7	133.3	33.3	4.2	>166.7
**5**	66.7	133.3	33.3	8.3	>166.7
Neomycin ^a^	1.7	1.0	2.1	4.2	
Benomyl ^b^					4.2 ± 0.4

^a^ positive control for antibacterial assay.

^b^ positive control for antifungal assay.

^*^ 20% inhibitory activity was observed at a concentration of 133.3 µg/Ml.

The isolates were also evaluated against Gram-negative plant pathogenic bacteria, such as *D. chrysanthemi*, *P. carotovorum* ssp. *carotovorum*, *P. atrosepticum*, *X. citri* pv. *malvacearum*, *P. syringae* pv. *tomato*, *P. syringae* pv. *maculicola*, and *A. tumefaciens*, but remained inactive at the studied concentrations (maximum tested concentration: 133.3 μg/mL).

In previous studies, shoreic acid (**1**) showed cytotoxic (Farabi et al., 2017) and antimycobacterial (Leitão et al., 2008) effects, while the epimeric mixture of shoreic and eichlerianic acid exhibited moderate antibacterial activity against *B. subtilis, Staphylococcus aureus*, *Escherichia coli*, and *Pseudomonas aeruginosa* (Hutagaol et al., 2025). In agar plate diffusion tests, both 13-HODE (**4**) and 9-HODE (**5**) were found to be active against *B. subtilis*, *S. aureus*, and *Micrococcus flavus* (Mundt et al., 2003), while in another study, 13-HODE (**4**) also demonstrated antibacterial effect against *Streptococcus pyogenes* and *P. aeruginosa* (Gebrehiwot et al., 2024). The antibacterial activity of 13-HODE (**4**) and 9-HODE (**5**) against *B. subtilis*, isolated from *A. altissima* stem bark, was previously confirmed in our laboratory by a microdilution assay (Cselőtey et al., 2024).

Our results indicate that the isolated compounds are highly specific, showing pronounced selectivity against Gram-positive plant pathogenic bacteria. This result is particularly relevant; although the majority of economically important bacterial plant pathogens belong to Gram-negative groups, there are several Gram-positive species that also cause substantial economic damage (Mansfield et al., 2012; Sharma et al., 2025). Generally, Gram-positive bacteria are typically more susceptible to terpene treatments, including triterpenes (Khameneh et al., 2019). The intrinsic resistance and lower susceptibility of Gram-negative bacteria to the hydrophobic isolated compounds (**1**–**5**), **1**–**3** identified as triterpenes, may be explained by the fundamental structural differences between Gram-positive and Gram-negative bacteria. This is because, the outer membrane of Gram-negative bacteria - located external to the peptidoglycan layer - acts as an effective permeability barrier, restricting the penetration of many lipophilic natural products and thereby reducing their antibacterial efficacy. The hydrophilic nature of this outer membrane, primarily due to embedded lipopolysaccharide (LPS) molecules, inherently may reduce the uptake of lipophilic terpenes (Nikaido, 2003; May and Grabowicz, 2018). In addition, terpenes diffuse relatively easily through the porous peptidoglycan layer of Gram-positive bacteria, while the lipoteichoic acid constituents present in their cell membranes may further enhance the cellular uptake of these hydrophobic molecules (Nazzaro et al., 2013; Miörner et al., 1983). Furthermore, terpenes have been shown to inhibit efflux pumps more frequently and effectively in Gram-positive bacteria than in Gram-negative species, which further contributes to the higher susceptibility of Gram-positive phytopathogens (Dias et al., 2022). Additionally, the selective porin channels in the outer membrane of Gram-negative bacteria favor the passage of small, hydrophilic molecules, making it difficult for hydrophobic compounds to pass through (Galdiero et al., 2012). From a practical perspective, our findings suggest that the isolated secondary metabolites are unlikely to serve as broad-spectrum antibacterial agents for plant disease control. However, their selectivity could be highly relevant for targeted disease management. Their pronounced activity against several Gram-positive plant pathogens, particularly *C. michiganensis* and *R. fascians*, together with the moderate antifungal activity of compound **1** against *F. graminearum*, implies that they may serve as promising lead structures for developing more potent plant protection agents.

## 4 Conclusions

In this work, a non-targeted, effect-directed phytochemical analysis of the methanolic extracts of *A. altissima* petioles was carried out using TLC coupled with UV detection, derivatization with *p*-anisaldehyde reagent, direct bioautography using *B. subtilis*, and HRMS. The bioassay-guided isolation process yielded three antimicrobial *seco*-dammarane triterpenes (**1**–**3**), which were characterized by HR-HESI-MS/MS, one- and two-dimensional NMR spectroscopy, and polarimetry. Foveolin C (**2**) and (20*S*,24*R*)-epoxy-25-hydroxy-A-homo-4-oxadammaran-3-one (**3**) are reported here as new natural products, while the known shoreic acid (**1**) and the previously isolated 13-HODE (**4**) and 9-HODE (**5**) were identified in *A. altissima* petioles for the first time.

In microdilution assays, compound **1** exhibited strong antibacterial activity against the plant pathogenic bacteria *C. michiganensis* and *R. fascians*, with MIC values two-fold lower than those of the positive control neomycin. Among the isolates, compound **1** also showed the highest antimicrobial activity against *B. subtilis*, *C. flaccumfaciens* pv. *flaccumfaciens*, and the fungal plant pathogen *F. graminearum*. Compound **2** demonstrated notable antimicrobial activity against all tested pathogens, while compounds **3**–**5** showed weak to moderate activity overall, with more pronounced antibacterial effects against *R. fascians*. After proper structure optimization, these compounds could become valuable leads for the development of novel antimicrobial agents. Future research can evaluate their antimicrobial efficacy under *in planta* conditions, along with the assessment of their phytotoxicity, chemical stability, and environmental safety profile. In addition, future studies can investigate formulations to assess their feasibility as sustainable plant protection products.

## Data Availability

The original contributions presented in the study are included in the article/**Supplementary Material**, further inquiries can be directed to the corresponding author/s.

## References

[B1] Andonova T. G. Dimitrova-Dyulgerova I. Z. Slavov Z. Dincheva I. N. Stoyanova A. S. (2021). Volatile compounds in flowers, samaras, leaves and stem bark of *Ailanthus altissima* (Mill.) Swingle, growing in Bulgaria. Iop Conf. Ser. Mater. Sci. Eng. 1031, 12087. doi: 10.1088/1757-899X/1031/1/012087

[B2] Asefa E. M. Damtew Y. T. Ober J. (2024). Pesticide water pollution, human health risks, and regulatory evaluation: a nationwide analysis in Ethiopia. J. Hazard. Mater. 478, 135326. doi: 10.1016/j.jhazmat.2024.135326 39116746

[B3] Baglyas M. Bozsó Z. Schwarczinger I. Ott P. G. Bakonyi J. Darcsi A. . (2025). Discovery of undescribed clerodane diterpenoids with antimicrobial activity isolated from the roots of *Solidago gigantea* Ait. Int. J. Mol. Sci. 26, 9187. doi: 10.3390/ijms26189187 41009749 PMC12471146

[B4] Bailly C. (2020). Anticancer properties and mechanism of action of the quassinoid ailanthone. Phytother. Res. 34, 2203–2213. doi: 10.1002/ptr.6681 32239572

[B5] Barringer L. Ciafré C. M. (2020). Worldwide feeding host plants of spotted lanternfly, with significant additions from North America. Environ. Entomol. 49, 999–1011. doi: 10.1093/ee/nvaa093 32797186

[B6] Basu P. Ngo H. T. Aizen M. A. Garibaldi L. A. Gemmill-Herren B. Imperatriz-Fonseca V. . (2024). Pesticide impacts on insect pollinators: current knowledge and future research challenges. Sci. Total Environ. 954, 176656. doi: 10.1016/j.scitotenv.2024.176656 39366587

[B7] Cselőtey A. Baglyas M. Király N. Ott P. G. Glavnik V. Vovk I. . (2024). Bioassay-guided isolation and identification of antibacterial compounds from invasive tree of heaven stem and trunk bark. Molecules 29, 5846. doi: 10.3390/molecules29245846 39769934 PMC11680021

[B8] Cui W. Quan X. Tao K. Teng Y. Zhang X. Liu Y. . (2009). Mechanism of action of neomycin on *Erwinia carotovora* subsp. *carotovora*. Pest. Biochem. Physiol. 95, 85–89. doi: 10.1016/j.pestbp.2009.07.002 38826717

[B9] Cuppels D. A. (1986). Generation and characterization of Tn5 insertion mutations in *Pseudomonas syringae* pv. *tomato*. Appl. Environ. Microbiol. 51, 323–327. doi: 10.1128/aem.51.2.323-327.1986 16346988 PMC238867

[B10] Dai J. Li N. Wang J. Schneider U. (2016). Fruitful decades for canthin-6-ones from 1952 to 2015: biosynthesis, chemistry, and biological activities. Molecules 21, 493. doi: 10.3390/molecules21040493 27092482 PMC6274392

[B11] Deblaere R. Bytebier B. De Greve H. Deboeck F. Schell J. Van Montagu M. . (1985). Efficient octopine Ti plasmid-derived vectors for *Agrobacterium*-mediated gene transfer to plants. Nucleic Acids Res. 13, 4777–4788. doi: 10.1093/nar/13.13.4777 4022773 PMC321826

[B12] De Feo V. De Martino L. Quaranta E. Pizza C. (2003). Isolation of phytotoxic compounds from tree-of-heaven (*Ailanthus altissima* Swingle). J. Agric. Food. Chem. 51, 1177–1180. doi: 10.1021/jf020686+ 12590453

[B13] Demeter A. Saláta D. Kovács E. T. Szirmai O. Trenyik P. Meinhardt S. . (2021). Effects of the invasive tree species *Ailanthus altissima* on the floral diversity and soil properties in the Pannonian region. Land 10, 1155. doi: 10.3390/LAND10111155 30654563

[B14] Dias K. J. S. O. Miranda G. M. Bessa J. R. Araújo A. C. J. Freitas P. R. Almeida R. S. . (2022). Terpenes as bacterial efflux pump inhibitors: a systematic review. Front. Pharmacol. 13, 953982. doi: 10.3389/fphar.2022.953982 36313340 PMC9606600

[B15] Fan J. Crooks C. Lamb C. (2008). High-throughput quantitative luminescence assay of the growth *in planta* of *Pseudomonas syringae* chromosomally tagged with *Photorhabdus luminescens luxCDABE*. Plant J. 53, 393–399. doi: 10.1111/j.1365-313X.2007.03303.x 17971037

[B16] Farabi K. Harneti D. Nurlelasari Maharani R. Hidayat A. T. Awang K. . (2017). New cytotoxic protolimonoids from the stem bark of *Aglaia argentea* (Meliaceae). Phytochem. Lett. 21, 211–215. doi: 10.1016/J.PHYTOL.2017.07.006 38826717

[B17] Farouil L. Sylvestre M. Fournet A. Cebrián-Torrejón G. (2022). Review on canthin-6-one alkaloids: distribution, chemical aspects and biological activities. Eur. J. Med. Chem. Rep. 5, 100049. doi: 10.1016/j.ejmcr.2022.100049 38826717

[B18] Galdiero S. Falanga A. Cantisani M. Tarallo R. Della Pepa M. E. D'Oriano V. . (2012). Microbe-host interactions: structure and role of Gram-negative bacterial porins. Curr. Protein Pept. Sci. 13, 843–854. doi: 10.2174/138920312804871120 23305369 PMC3706956

[B19] Gao Z. H. Duan Z. K. Ma Z. T. Ye L. Yao G. D. Huang X. X. . (2022). Chouchunsteride A–D, four new steroids from the leaves of *Ailanthus altissima* (Mill.) Swingle. Steroids 188, 109117. doi: 10.1016/j.steroids.2022.109117 36181833

[B20] Gebrehiwot H. Ensermu U. Dekebo A. Endale M. Duke T. N. (2024). *In vitro* antibacterial and antioxidant activities, pharmacokinetics, and *in silico* molecular docking study of phytochemicals from the roots of *Ziziphus spina-christi*. Biochem. Res. Int. 2024, 7551813. doi: 10.1155/2024/7551813 39263680 PMC11390196

[B21] Goulson D. Nicholls E. Botías C. Rotheray E. L. (2015). Bee declines driven by combined stress from parasites, pesticides, and lack of flowers. Science 347, 1255957. doi: 10.1126/science.1255957 25721506

[B22] Guo S. S. Duan Z. K. Huang X. X. Song S. J. (2023). Phytochemical investigation on the leaves of *Ailanthus altissima* (Mill.) Swingle. Biochem. Syst. Ecol. 110, 104668. doi: 10.1016/J.BSE.2023.104668 38826717

[B23] Heisey R. M. (1996). Identification of an allelopathic compound from *Ailanthus altissima* (Simaroubaceae) and characterization of its herbicidal activity. Am. J. Bot. 83, 192–200. doi: 10.1002/j.1537-2197.1996.tb12697.x 41531421

[B24] Hong Z. L. Xiong J. Wu S. B. Zhu J. J. Hong J. L. Zhao Y. . (2013). Tetracyclic triterpenoids and terpenylated coumarins from the bark of *Ailanthus altissima* (tree of heaven). Phytochemistry 86, 159–167. doi: 10.1016/j.phytochem.2012.10.008 23153518

[B25] Hutagaol R. P. Mozef T. Nurilmala F. Primahana G. Fajriah S. Prastya M. E. . (2025). Dammarane-type triterpenoids from twigs of *Aglaia foveolata* and their antibacterial activity. Molekul 20, 394–403. doi: 10.20884/1.jm.2025.20.2.16241

[B26] Khameneh B. Iranshahy M. Soheili V. Fazly Bazzaz B. S. (2019). Review on plant antimicrobials: a mechanistic viewpoint. Antimicrob. Resist. Infect. Control 8, 118. doi: 10.1186/S13756-019-0559-6 31346459 PMC6636059

[B27] Koch E. Löwen A. Kampschulte N. Plitzko K. Wiebel M. Rund K. M. . (2023). Beyond autoxidation and lipoxygenases: fatty acid oxidation products in plant oils. J. Agric. Food. Chem. 71, 13092–13106. doi: 10.1021/ACS.JAFC.3C02724 37624576

[B28] Kowarik I. Säumel I. (2007). Biological flora of Central Europe: *Ailanthus altissima* (Mill.) Swingle. Perspect. Plant Ecol. Evol. Syst. 8, 207–237. doi: 10.1016/j.ppees.2007.03.002 38826717

[B29] Kubota K. Fukamiya N. Hamada T. Okano M. Tagahara K. Lee K. H. (1996). Two new quassinoids, ailantinols A and B, and related compounds from *Ailanthus altissima*. J. Nat. Prod. 59, 683–686. doi: 10.1021/np960427c 8759166

[B30] Lavie D. Frolow F. Meshulam H. (1984). The X-ray structure of methyl shoreate and the stereochemistry of eichlerianic acid, cabraleone and ocotillone. Tetrahedron 40, 419–420. doi: 10.1016/S0040-4020(01)91190-1

[B31] Leitão G. G. Abreu L. F. Costa F. N. Brum T. B. Ramos D. F. Silva P. E. A. . (2008). Preparative isolation of antimycobacterial shoreic acid from *Cabralea canjerana* by high speed countercurrent chromatography. Nat. Prod. Commun. 3, 1995–1997. doi: 10.1177/1934578X0800301211

[B32] Li X. Li Y. Ma S. Zhao Q. Wu J. Duan L. . (2021). Traditional uses, phytochemistry, and pharmacology of *Ailanthus altissima* (Mill.) Swingle bark: a comprehensive review. J. Ethnopharmacol. 275, 114121. doi: 10.1016/j.jep.2021.114121 33862103

[B33] Mansfield J. Genin S. Magori S. Citovsky V. Sriariyanum M. Ronald P. . (2012). Top 10 plant pathogenic bacteria in molecular plant pathology. Mol. Plant Pathol. 13, 614–629. doi: 10.1111/j.1364-3703.2012.00804.x 22672649 PMC6638704

[B34] May K. L. Grabowicz M. (2018). The bacterial outer membrane is an evolving antibiotic barrier. Proc. Natl. Acad. Sci. U.S.A. 115, 8852–8854. doi: 10.1073/pnas.1812779115 30139916 PMC6130387

[B35] Miörner H. Johansson G. Kronvall G. (1983). Lipoteichoic acid is the major cell wall component responsible for surface hydrophobicity of group A streptococci. Infect. Immun. 39, 336–343. doi: 10.1128/iai.39.1.336-343.1983 6337099 PMC347944

[B36] Mohamed H. R. El-Wakil E. A. Abdel-Hameed E. S. S. El-Hashash M. M. Shemis M. (2021). Evaluation of total phenolics, flavonoids, and antioxidant and cytotoxic potential of *Ailanthus altissima* (Mill.) Swingle leaves. J. Rep. Pharm. Sci. 10, 130–136. doi: 10.4103/jrptps.JRPTPS_7_21 42435161

[B37] Móricz Á.M. Häbe T. T. Böszörményi A. Ott P. G. Morlock G. E. (2015). Tracking and identification of antibacterial components in the essential oil of *Tanacetum vulgare* L. by the combination of high-performance thin-layer chromatography with direct bioautography and mass spectrometry. J. Chromatogr. A 1422, 310–317. doi: 10.1016/j.chroma.2015.10.010 26499972

[B38] Móricz Á.M. Krüzselyi D. Alberti Á. Darcsi A. Horváth G. Csontos P. . (2017). Layer chromatography-bioassays directed screening and identification of antibacterial compounds from scotch thistle. J. Chromatogr. A 1524, 266–272. doi: 10.1016/j.chroma.2017.09.062 28989030

[B39] Muhammad Abdur Rahman H. Javaid S. Ashraf W. Fawad Rasool M. Saleem H. Ali Khan S. . (2023). Effects of long-term *Ailanthus altissima* extract supplementation on fear, cognition and brain antioxidant levels. Saudi Pharm. J. 31, 191–206. doi: 10.1016/j.jsps.2022.12.003 36942273 PMC10023549

[B40] Mundt S. Kreitlow S. Jansen R. (2003). Fatty acids with antibacterial activity from the cyanobacterium *Oscillatoria redekei* HUB 051. J. Appl. Phycol. 15, 263–267. doi: 10.1023/A:1023889813697 41886696

[B41] Nazzaro F. Fratianni F. De Martino L. Coppola R. De Feo V. (2013). Effect of essential oils on pathogenic bacteria. Pharmaceuticals 6, 1451–1474. doi: 10.3390/ph6121451 24287491 PMC3873673

[B42] Nikaido H. (2003). Molecular basis of bacterial outer membrane permeability revisited. Microbiol. Mol. Biol. Rev. 67, 593–656. doi: 10.1128/mmbr.67.4.593-656.2003 14665678 PMC309051

[B43] Ohmoto T. Tanaka R. Nikaido T. (1976). Studies on the constituents of *Ailanthus altissima* Swingle. On the alkaloidal constituents. Chem. Pharm. Bull. 24, 1532–1536. doi: 10.1248/cpb.24.1532 39482488

[B44] Pan L. Kardono L. B. S. Riswan S. Chai H. Carcache De Blanco E. J. Pannell C. M. . (2010). Isolation and characterization of minor analogues of silvestrol and other constituents from a large-scale re-collection of *Aglaia foveolata*. J. Nat. Prod. 73, 1873–1878. doi: 10.1021/np100503q 20939540 PMC2993763

[B45] Pedersini C. Bergamin M. Aroulmoji V. Baldini S. Picchio R. Pesce P. G. . (2011). Herbicide activity of extracts from *Ailanthus altissima* (Simaroubaceae). Nat. Prod. Commun. 6, 593–596. doi: 10.1177/1934578x1100600504 21615014

[B46] Pirani J. R. Majure L. C. Devecchi M. F. (2022). An updated account of Simaroubaceae with emphasis on American taxa. Braz. J. Bot. 45, 201–221. doi: 10.1007/s40415-021-00731-x 30311153

[B47] Poljuha D. Sladonja B. Šola I. Dudaš S. Bilić J. Rusak G. . (2017). Phenolic composition of leaf extracts of *Ailanthus altissima* (Simaroubaceae) with antibacterial and antifungal activity equivalent to standard antibiotics. Nat. Prod. Commun. 12, 1609–1612. doi: 10.1177/1934578x1701201021

[B48] Qureshi H. Anwar T. (2024). Role of allelopathy in invasion of invasive species. Allelopathy J. 62, 115–138. doi: 10.26651/allelo.j/2024-62-2-1490

[B49] Rahman S. Fukamiya N. Okano M. Tagahara K. Lee K. H. (1997). Anti-tuberculosis activity of quassinoids. Chem. Pharm. Bull. 45, 1527–1529. doi: 10.1248/cpb.45.1527 9332005

[B50] Roux D. Martin M. T. Adeline M. T. Sevenet T. Hadi A. H. A. Païs M. (1998). Foveolins A and B, dammarane triterpenes from *Aglaia foveolata*. Phytochemistry 49, 1745–1748. doi: 10.1016/S0031-9422(98)00305-7 11711093

[B51] Seger C. Pointinger S. Greger H. Hofer O. (2008). Isoeichlerianic acid from *Aglaia silvestris* and revision of the stereochemistry of foveolin B. Tetrahedron Lett. 49, 4313–4315. doi: 10.1016/J.TETLET.2008.04.109 38826717

[B52] Seon W. H. Jong R. L. Lee J. Hyun S. K. Min S. Y. Ki H. P. (2005). New coumarins from the *Ailanthus altissima*. Heterocycles 65, 1963–1966. doi: 10.3987/COM-05-10429

[B53] Sharma K. Yadav R. Sharma J. Devi E. P. (2025). Virulence mechanism in Gram negative plant pathogenic bacteria. Int. J. Emerg. Technol. 16, 102–107. doi: 10.65041/ijet.2025.16.2.15

[B54] Sladonja B. Sušek M. Guillermic J. (2015). Review on invasive tree of heaven (*Ailanthus altissima* (Mill.) Swingle) conflicting values: assessment of its ecosystem services and potential biological threat. Environ. Manage. 56, 1009–1034. doi: 10.1007/s00267-015-0546-5 26071766

[B55] Soler J. Izquierdo J. (2024). The invasive *Ailanthus altissima*: a biology, ecology, and control review. Plants 13, 931. doi: 10.3390/plants13070931 38611460 PMC11013224

[B56] Tao K. Fan J. Shi G. Zhang X. Zhao H. Hou T. (2011). *In vivo* and *in vitro* antibacterial activity of neomycin against plant pathogenic bacteria. Sci. Res. Essays 6, 6829–6834. doi: 10.5897/sre11.552

[B57] Trapp M. A. Kai M. Mithöfer A. Rodrigues-Filho E. (2015). Antibiotic oxylipins from *Alternanthera brasiliana* and its endophytic bacteria. Phytochemistry 110, 72–82. doi: 10.1016/j.phytochem.2014.11.005 25433629

[B58] Tudi M. Ruan H. D. Wang L. Lyu J. Sadler R. Connell D. . (2021). Agriculture development, pesticide application and its impact on the environment. Int. J. Environ. Res. Public Health 18, 1112. doi: 10.3390/ijerph18031112 33513796 PMC7908628

[B59] Vovk I. Glavnik V. Strgulc Krajšek S. Bensa M. Ott P. G. Móricz Á.M. (2026). Effect-directed analyses of bioactives in tree of heaven (*Ailanthus altissima* (Mill.) Swingle). Plants 15, 1026. doi: 10.3390/PLANTS15071026 41977685 PMC13074743

[B60] Yang X. L. Yuan Y. L. Zhang D. M. Li F. Ye W. C. (2014). Shinjulactone O, a new quassinoid from the root bark of *Ailanthus altissima*. Nat. Prod. Res. 28, 1432–1437. doi: 10.1080/14786419.2014.909418 24967875

[B61] Zhu T. H. Wang Y. F. Jiang T. Zhao L. K. Mi H. Ji Y. N. . (2021). Two new lignans from the fresh bark of *Ailanthus altissima*. Chem. Nat. Compd. 57, 422–424. doi: 10.1007/s10600-021-03379-x 30311153

[B62] Zúñiga-Venegas L. A. Hyland C. Muñoz-Quezada M. T. Quirós-Alcalá L. Butinof M. Buralli R. . (2022). Health effects of pesticide exposure in Latin American and the Caribbean populations: a scoping review. Environ. Health Perspect. 130, 96002. doi: 10.1289/EHP9934 36173136 PMC9521041

