# Supplementary material for: Novel seco-dammarane triterpenes with antimicrobial activity against plant pathogens from Ailanthus altissima petioles
Source: Front Plant Sci. 2026 Aug 11;17:1888371. doi: 10.3389/fpls.2026.1888371 (PMC13503308; doi:10.3389/fpls.2026.1888371)
Supplement: Supplementary file 1 [file DataSheet1.pdf]

Supplementary Material

Novel *seco*-Dammarane Triterpenes with Antimicrobial Activity against Plant Pathogens from *Ailanthus altissima* Petioles

Anna Cselőtey[1,2,*], Márton Baglyas[1,2], Panna Aschenbrenner[1], Zoltán Bozsó[1], Ildikó Schwarczinger[1], József Bakonyi[1], András Darcsi[3], Vesna Glavnik[4], Irena Vovk[4] and Ágnes M. Móricz[1,2,*]

[1]Plant Protection Institute, HUN-REN Centre for Agricultural Research, Fehérvári út 132–144, H-1116 Budapest, Hungary

[2]Doctoral School, Semmelweis University, Üllői út 26, H-1085 Budapest, Hungary

[3]Pharmaceutical Chemistry and Technology Department, National Center for Public Health and Pharmacy, Szabolcs utca 33, H-1135 Budapest, Hungary

[4]Laboratory for Food Chemistry, National Institute of Chemistry, Hajdrihova 19, 1000 Ljubljana, Slovenia

***Correspondence**:

Anna Cselőtey (e-mail: cselotey.anna@stud.semmelweis.hu)

Ágnes M. Móricz (e-mail: moricz.agnes@atk.hun-ren.hu)

Table of Contents

No.	Legend	Page
**Table S1.**	Collection area and time of the *A. altissima* petiole samples.	S-5
**Table S2.**	Comparison of the recorded (in this study) and the previously reported ^{1}H and ^{13}C NMR spectroscopic data in chloroform-*d* ($CDCl_3$) of shoreic acid (**1**).	S-6
**Table S3.**	Comparison of the ^{1}H NMR spectroscopic data in chloroform-*d* ($CDCl_3$) of the previously undescribed foveolin C (**2**) and the known foveolin A (**2a**) and foveolin B (**2b**).	S-7
**Table S4.**	Comparison of the ^{13}C NMR spectroscopic data in chloroform-*d* ($CDCl_3$) of the previously undescribed foveolin C (**2**) and the known foveolin A (**2a**) and foveolin B (**2b**).	S-8
**Table S5.**	Comparison of the ^{1}H NMR spectroscopic data (600 MHz, $CDCl_3$) of the previously undescribed (20*S*,24*R*)-epoxy-25-hydroxy-A-homo-4-oxadammaran-3-one (**3**) and the known (20*S*,24*S*)-epoxy-25-hydroxy-A-homo-4-oxadammaran-3-one (**3a**) and (20*R*,24*S*)-epoxy-25-hydroxy-A-homo-4-oxadammaran-3-one (**3b**).	S-9
**Table S6.**	Comparison of the ^{13}C NMR spectroscopic data (151 MHz, $CDCl_3$) of the previously undescribed (20*S*,24*R*)-epoxy-25-hydroxy-A-homo-4-oxadammaran-3-one (**3**) and the known (20*S*,24*S*)-epoxy-25-hydroxy-A-homo-4-oxadammaran-3-one (**3a**) and (20*R*,24*S*)-epoxy-25-hydroxy-A-homo-4-oxadammaran-3-one (**3b**).	S-10
**Figure S1.**	TLC chromatograms of the *A. altissima* petiole extracts (H2–H6, H16, H18–H21, B8–B10), developed with toluene–isopropyl acetate–methanol (5:4:1, *V*/*V*), before (at 254 nm (**a**) and 366 nm (**b**)) and after derivatization with *p*-anisaldehyde reagent under white light (**c**).	S-11
**Figure S2.**	TLC chromatograms at 254 nm (**a**) and 366 nm (**b**), and TLC–*B. subtilis* bioautogram under white light (**c**) of the *A. altissima* petiole extracts (H2–H7, L1, L3, L4, B1, B3, B5), developed with toluene–isopropyl acetate–methanol (5:4:1, *V*/*V*).	S-11
**Figure S3.**	TLC chromatograms (**a**, **b**, **c**) and bioautogram (**d**) of the merged flash chromatography fractions (30–33, 34–38, 39–43, 44–52), developed with toluene–isopropyl acetate–methanol (5:4:1, *V*/*V*), detected before (at 254 nm (**a**) and 366 nm (**b**)) and after derivatization with *p*-anisaldehyde reagent under white light (**c**), and *B. subtilis* bioassay under white light (**d**), as well as TLC–HESI^{-}-HRMS spectrum recorded from the zones of compounds **4** and **5** (fraction 34–38) obtained via the elution head-based TLC-MS interface and background subtraction.	S-11
**Figure S4.**	HPLC–HRMS(/MS) total ion chromatograms (TICs) and extracted ion chromatograms (EICs) of the flash chromatography fraction H19/34–38, and the compounds **4** and **5**.	S-12
**Figure S5.**	Chemical structures of the previously undescribed foveolin C (**2**) (20*S*,24*R* isomer) and the known foveolin A (**2a**) (20*S*,24*S* isomer) and foveolin B (**2b**) (20*R*,24*S* isomer).	S-13
**Figure S6.**	Chemical structures of the previously undescribed (20*S*,24*R*)-epoxy-25-hydroxy-A-homo-4-oxadammaran-3-one (**3**) (20*S*,24*R* isomer)	S-13

	and the known (20*S*,24*S*)-epoxy-25-hydroxy-A-homo-4-oxadammaran-3-one (**3a**) (20*S*,24*S* isomer) and (20*R*,24*S*)-epoxy-25-hydroxy-A-homo-4-oxadammaran-3-one (**3b**) (20*R*,24*S* isomer).	
**Figure S7.**	^{1}H NMR spectrum of shoreic acid (**1**) (600 MHz, $CDCl_3$).	S-14
**Figure S8.**	^{1}H NMR spectrum of shoreic acid (**1**) (600 MHz, $CDCl_3$) – expansion (δ_H 5.1–3.5).	S-14
**Figure S9.**	^{1}H NMR spectrum of shoreic acid (**1**) (600 MHz, $CDCl_3$) – expansion (δ_H 2.6–0.65).	S-15
**Figure S10.**	^{13}C DEPTQ NMR spectrum of shoreic acid (**1**) (151 MHz, $CDCl_3$).	S-15
**Figure S11.**	^{1}H–^{1}H COSY NMR spectrum of shoreic acid (**1**) (600 MHz, $CDCl_3$).	S-16
**Figure S12.**	^{1}H–^{13}C multiplicity-edited HSQC NMR spectrum of shoreic acid (**1**) (600/151 MHz, $CDCl_3$).	S-16
**Figure S13.**	^{1}H–^{13}C HMBC NMR spectrum of shoreic acid (**1**) (600/151 MHz, $CDCl_3$).	S-17
**Figure S14.**	^{1}H–^{1}H TOCSY NMR spectrum of shoreic acid (**1**) (600 MHz, $CDCl_3$).	S-17
**Figure S15.**	^{1}H–^{1}H ROESY NMR spectrum of shoreic acid (**1**) (600 MHz, $CDCl_3$).	S-18
**Figure S16.**	HR-ESI^{+}-MS spectrum of shoreic acid (**1**).	S-18
**Figure S17.**	HR-ESI^{+}-MS/MS of shoreic acid (**1**) with a normalized HCD collision energy of 15%. Precursor ion: *m*/*z* 475.3775 $[M+H]^{+}$, $C_{30}H_{51}O_4^{+}$.	S-19
**Figure S18.**	HR-ESI^{-}-MS spectrum of shoreic acid (**1**).	S-19
**Figure S19.**	HR-ESI^{-}-MS/MS of shoreic acid (**1**) with a normalized HCD collision energy of 60%. Precursor ion: *m*/*z* 473.3634 $[M-H]^{-}$, $C_{30}H_{49}O_4^{-}$.	S-19
**Figure S20.**	^{1}H NMR spectrum of foveolin C (**2**) (600 MHz, $CDCl_3$).	S-20
**Figure S21.**	^{1}H NMR spectrum of foveolin C (**2**) (600 MHz, $CDCl_3$) – expansion (δ_H 4.0–0.65).	S-20
**Figure S22.**	^{13}C DEPTQ NMR spectrum of foveolin C (**2**) (151 MHz, $CDCl_3$).	S-21
**Figure S23.**	^{1}H–^{1}H COSY NMR spectrum of foveolin C (**2**) (600 MHz, $CDCl_3$).	S-21
**Figure S24.**	^{1}H–^{13}C multiplicity-edited HSQC NMR spectrum of foveolin C (**2**) (600/151 MHz, $CDCl_3$).	S-22
**Figure S25.**	^{1}H–^{13}C band-selective HSQC NMR spectrum of foveolin C (**2**) (600/151 MHz, $CDCl_3$) – aliphatic region (δ_C 35.5–27.0).	S-22
**Figure S26.**	^{1}H–^{13}C HMBC NMR spectrum of foveolin C (**2**) (600/151 MHz, $CDCl_3$).	S-23
**Figure S27.**	^{1}H–^{13}C band-selective HMBC NMR spectrum of foveolin C (**2**) (600/151 MHz, $CDCl_3$) – aliphatic region (δ_C 35.5–27.0).	S-23
**Figure S28.**	^{1}H–^{1}H TOCSY NMR spectrum of foveolin C (**2**) (600 MHz, $CDCl_3$).	S-24
**Figure S29.**	^{1}H–^{1}H ROESY NMR spectrum of foveolin C (**2**) (600 MHz, $CDCl_3$).	S-24
**Figure S30.**	HR-ESI^{+}-MS spectrum of foveolin C (**2**).	S-25
**Figure S31.**	HR-ESI^{+}-MS/MS spectrum of foveolin C (**2**) with a normalized HCD collision energy of 50%. Precursor ion: *m*/*z* 515.3707 $[M+Na]^{+}$, $C_{30}H_{52}O_5Na^{+}$.	S-25
**Figure S32.**	HR-ESI^{-}-MS spectrum of foveolin C (**2**).	S-25

**Figure S33.**	HR-ESI$^-$-MS/MS spectrum of foveolin C (**2**) with a normalized HCD collision energy of 50%. Precursor ion: *m*/*z* 491.3737 [M−H]$^-$, $C_{30}H_{51}O_5^-$.	S-26
**Figure S34.**	UV spectrum of foveolin C (**2**) in ethanol.	S-26
**Figure S35.**	ATR-FTIR spectrum of foveolin C (**2**).	S-27
**Figure S36.**	^{1}H NMR spectrum of (20*S*,24*R*)-epoxy-25-hydroxy-A-homo-4-oxadammaran-3-one (**3**) (600 MHz, $CDCl_3$).	S-28
**Figure S37.**	^{1}H NMR spectrum of (20*S*,24*R*)-epoxy-25-hydroxy-A-homo-4-oxadammaran-3-one (**3**) (600 MHz, $CDCl_3$) – expansion (δ_H 4.0–0.65).	S-28
**Figure S38.**	^{13}C DEPTQ NMR spectrum of (20*S*,24*R*)-epoxy-25-hydroxy-A-homo-4-oxadammaran-3-one (**3**) (151 MHz, $CDCl_3$).	S-29
**Figure S39.**	^{1}H–^{1}H COSY NMR spectrum of (20*S*,24*R*)-epoxy-25-hydroxy-A-homo-4-oxadammaran-3-one (**3**) (600 MHz, $CDCl_3$).	S-29
**Figure S40.**	^{1}H–^{13}C multiplicity-edited HSQC NMR spectrum of (20*S*,24*R*)-epoxy-25-hydroxy-A-homo-4-oxadammaran-3-one (**3**) (600/151 MHz, $CDCl_3$).	S-30
**Figure S41.**	^{1}H–^{13}C band-selective HSQC NMR spectrum of (20*S*,24*R*)-epoxy-25-hydroxy-A-homo-4-oxadammaran-3-one (**3**) (600/151 MHz, $CDCl_3$) – aliphatic region (δ_C 41.5–22.0).	S-30
**Figure S42.**	^{1}H–^{13}C HMBC NMR spectrum of (20*S*,24*R*)-epoxy-25-hydroxy-A-homo-4-oxadammaran-3-one (**3**) (600/151 MHz, $CDCl_3$).	S-31
**Figure S43.**	^{1}H–^{13}C band-selective HMBC NMR spectrum of (20*S*,24*R*)-epoxy-25-hydroxy-A-homo-4-oxadammaran-3-one (**3**) (600/151 MHz, $CDCl_3$) – aliphatic region (δ_C 41.5–14.0).	S-31
**Figure S44.**	^{1}H–^{1}H TOCSY NMR spectrum of (20*S*,24*R*)-epoxy-25-hydroxy-A-homo-4-oxadammaran-3-one (**3**) (600 MHz, $CDCl_3$).	S-32
**Figure S45.**	^{1}H–^{1}H ROESY NMR spectrum of (20*S*,24*R*)-epoxy-25-hydroxy-A-homo-4-oxadammaran-3-one (**3**) (600 MHz, $CDCl_3$).	S-32
**Figure S46.**	HR-ESI$^+$-MS spectrum of (20*S*,24*R*)-epoxy-25-hydroxy-A-homo-4-oxadammaran-3-one (**3**).	S-33
**Figure S47.**	HR-ESI$^+$-MS/MS spectrum of (20*S*,24*R*)-epoxy-25-hydroxy-A-homo-4-oxadammaran-3-one (**3**) with a normalized HCD collision energy of 15%. Precursor ion: *m*/*z* 475.3770 [M+H]$^+$, $C_{30}H_{51}O_4Na^+$.	S-33
**Figure S48.**	HR-ESI$^-$-MS spectrum of (20*S*,24*R*)-epoxy-25-hydroxy-A-homo-4-oxadammaran-3-one (**3**).	S-34
**Figure S49.**	HR-ESI$^-$-MS/MS spectrum of (20*S*,24*R*)-epoxy-25-hydroxy-A-homo-4-oxadammaran-3-one (**3**) with a normalized HCD collision energy of 50%. Precursor ion: *m*/*z* 473.3632 [M−H]$^-$, $C_{30}H_{49}O_4^-$.	S-34
**Figure S50.**	UV spectrum of (20*S*,24*R*)-epoxy-25-hydroxy-A-homo-4-oxadammaran-3-one (**3**) in ethanol.	S-35
**Figure S51.**	ATR-FTIR spectrum of (20*S*,24*R*)-epoxy-25-hydroxy-A-homo-4-oxadammaran-3-one (**3**).	S-35
	References	S-36

Table S1. Collection area and time of the *A. altissima* petiole samples.

Sample	Collection Area	Collection Time
H2	Harta	16 May 2022
H3	Harta	30 May 2022
H4	Harta	03 July 2022
H5/1[a]	Harta	13 August 2022
H5/2[b]	Harta	13 August 2022
H6	Harta	05 September 2022
H7	Harta	24 October 2022
H16	Harta	20 May 2023
H18	Harta	27 June 2023
H19	Harta	31 July 2023
H20	Harta	08 October 2023
H21	Harta	30 October 2023
L1	Leányfalu	21 May 2022
L3	Leányfalu	09 July 2022
L4	Leányfalu	15 October 2022
B1	Balatongyörök	23 May 2022
B3	Balatongyörök	26 July 2022
B5	Balatongyörök	17 October 2022
B8	Balatongyörök	07 June 2023
B9	Balatongyörök	08 August 2023
B10	Balatongyörök	21 October 2023

[a] tree with seeds

[b] tree without seeds

Table S2. Comparison of the recorded (in this study) and the previously reported ^{1}H and ^{13}C NMR spectroscopic data in chloroform-*d* ($CDCl_3$) of shoreic acid (**1**).

**Position**	**Recorded data**[a] (in this study)		**Previously reported data**[b] (Roux et al., 1998)	
	**δ_H (ppm), Multiplicity, (*J* in Hz)**	**δ_C (ppm)**	**δ_H (ppm), Multiplicity, (*J* in Hz)**	**δ_C (ppm)**
1a	1.61[a]	34.4		34.0
1b				
2a	2.38, ddd (15.9, 10.0, 6.4)	28.4	2.38[c]	28.1
2b	2.18, m		2.18[c]	
3	–	179.8		179.5
4	–	147.6		147.2
5	1.97, dd (12.7, 3.1)	50.9		50.5
6a	1.82[a]	24.7		24.3
6b	1.36, dq (13.5, 3.4)			
7a	1.53[a]	34.0		33.6
7b	1.23[a]			
8	–	40.2		39.8
9	1.51[a]	41.3		40.9
10	–	39.2		38.8
11a	1.42, m	22.2		22.9
11b	1.24[a]			
12a	1.79[a]	27.4		27.0
12b	1.24[a]			
13	1.59[a]	43.2		42.7
14	–	50.5		50.1
15a	1.46[a]	31.6		31.2
15b	1.10, m			
16a	1.78[a]	25.8		25.4
16b	1.46[a]			
17	1.81[a]	49.6		49.2
18	1.00, s	15.4		15.0
19	0.84, s	20.3	0.82, s	19.9
20	–	86.6		86.1
21	1.13, s	23.6	1.11, s	23.9
22a	1.70, m	35.9		35.5
22b	1.63[a]			
23a	1.87, m	26.3		25.9
23b	1.78[a]			
24	3.73, t (7.4)	83.5		83.1
25	–	71.7		71.4
26	1.21[b], s	27.5	1.18, s	27.1
27	1.12[b], s	24.4	1.10, s	24.3
28a	4.84, t (1.9)	113.6	4.82, br s	113.2
28b	4.66, m		4.63, br s	
29	1.73, s	23.3	1.70[c]	23.2
30	0.88, s	16.5	0.86, s	16.1

[a] recorded at 600/151 MHz
[b] recorded at 400/101 MHz
[c] multiplicity not reported in the publication

Table S3. Comparison of the ^{1}H NMR spectroscopic data in chloroform-*d* ($CDCl_3$) of the previously undescribed foveolin C (**2**) and the known foveolin A (**2a**) and foveolin B (**2b**).

	**Foveolin C (2)**[a] **(20*S*,24*R* isomer)** (in this study)	**Foveolin A (2a)**[b] **(20*S*,24*S* isomer)** (Roux et al., 1998)	**Foveolin B (2b)**[b] **(20*R*,24*S* isomer)** (Roux et al., 1998)
**Position**	**δ_H (ppm), (*J* in Hz)**		
2a	2.49, m	2.44[c]	2.44[c]
2b	2.20, m	2.18[c]	2.18[c]
18	0.97, s	0.96, s	0.96, s
19	1.01, s	0.99, s	0.99, s
21	1.13, s	1.12, s	1.12, s
24	**3.73, t (7.4)**	**3.62, dd (10, 5.5)**	**3.73, t (7)**
26	1.21, s	1.17, s	1.19, s
27	1.12, s	1.09, s	1.09[c]
28	1.31, s	1.29, s	1.29[c]
29	1.26, s	1.24, s	1.24, s
30	0.86, s	0.84, s	0.84, s

[a] recorded at 600 MHz
[b] recorded at 400 MHz
[c] multiplicity not provided in the publication

Table S4. Comparison of the ^{13}C NMR spectroscopic data in chloroform-*d* ($CDCl_3$) of the previously undescribed foveolin C (**2**) and the known foveolin A (**2a**) and foveolin B (**2b**).

	**Foveolin C (2)**[a] **(20*S*,24*R* isomer)** (in this study)	**Foveolin A (2a)**[b] **(20*S*,24*S* isomer)** (Roux et al., 1998)	**Foveolin B (2b)**[b] **(20*R*,24*S* isomer)** (Roux et al., 1998)
Position	δ_C (ppm)		
1	34.9	34.7	34.9
2	29.3	29.1	27.8
3	178.3	179.7	179.7
4	76.9	76.5	76.8
5	51.9	49.9	53.3
6	22.7	22.5	22.7
7	34.9	34.7	34.9
8	40.2	40.0	40.2
9	42.6	42.5	42.5
10	41.4	41.2	41.5
11	21.4	21.4	21.4
12	27.5	27.0	27.2
13	43.2	42.9	43.2
14	50.5	50.3	50.4
15	31.6	31.4	31.3
16	25.8	25.8	26.0
17	49.6	49.7	51.9
18	15.5	15.3	15.5
19	20.8	19.7	20.7
20	86.5	86.5	86.6
21	**23.6**	**27.0**	**22.0**
22	**36.0**	**34.8**	**37.5**
23	26.3	26.4	26.0
24	**83.5**	**86.3**	**84.5**
25	**71.6**	**70.3**	**71.6**
26	27.6	27.7	27.8
27	24.4	24.2	24.5
28	34.7	34.2	34.6
29	27.5	27.4	27.4
30	16.4	16.2	16.1

[a] recorded at 151 MHz
[b] recorded at 101 MHz

Table S5. Comparison of the ^{1}H NMR spectroscopic data (600 MHz, $CDCl_3$) of the previously undescribed (20*S*,24*R*)-epoxy-25-hydroxy-A-homo-4-oxadammaran-3-one (**3**) and the known (20*S*,24*S*)-epoxy-25-hydroxy-A-homo-4-oxadammaran-3-one (**3a**) and (20*R*,24*S*)-epoxy-25-hydroxy-A-homo-4-oxadammaran-3-one (**3b**).

	**(20*S*,24*R*)-epoxy-25-hydroxy-A-homo-4-oxadammaran-3-one (3)** (in this study)	**(20*S*,24*S*)-epoxy-25-hydroxy-A-homo-4-oxadammaran-3-one (3a)** (Pan et al., 2010)	**(20*R*,24*S*)-epoxy-25-hydroxy-A-homo-4-oxadammaran-3-one (3b)** (Pan et al., 2010)
**Position**	**δ_H (ppm), Multiplicity, (*J* in Hz)**		
1a	1.81[a]	1.82[a]	1.82[a]
1b	1.58[a]	1.63[a]	1.62[a]
2a	2.64, ddd (14.3, 12.8, 4.6)	2.67, td (14.0, 4.5)	2.65, td (14.0, 4.6)
2b	2.51, m	2.52, ddd (14.2, 5.7, 3.5)	
5	1.77, dd (12.5, 3.3)	1.80[a]	1.78[a]
6a	1.69[a]	1.77[a]	1.77[a]
6b	1.42[a]	1.44[a]	1.43 [a]
7a	1.53[a]	1.31[a]	1.30[a]
7b	1.29[a]		
9	1.43[a]	1.42[a]	1.42[a]
11a	1.52[a]	1.55[a]	1.55[a]
11b	1.28[a]	1.26[a]	1.26[a]
12a	1.80[a]	1.86[a]	2.03, br d (10.0)
12b	1.24[a]	1.10[a]	1.19[a]
13	1.63[a]	1.75[a]	1.75[a]
15a	1.45, m	1.45[a]	1.45[a]
15b	1.10[a]	1.10[a]	1.10[a]
16a	1.77[a]	1.78[a]	1.78[a]
16b	1.49[a]	1.30[a]	1.30[a]
17	1.82[a]	1.91[a]	1.79[a]
18	1.01, s	1.05, s	1.02, s
19	1.07, s	1.10, s	1.08, s
21	1.14, s	1.17, s	1.15, s
22a	1.70[a]	1.71[a]	1.71[a]
22b	1.64[a]		
23a	1.87, m	1.88[a]	1.80[a]
23b	1.79[a]	1.82[a]	
24	3.73, t (7.4)	3.65, dd (9.9, 5.6)	3.77, t (7.2)
26	1.21, s	1.21, s	1.20, s
27	1.12, s	1.13, s	1.10, s
28	1.48, s	1.50, s	1.48, s
29	1.41, s	1.42, s	1.40, s
30	0.86, s	0.88, s	0.86, s

[a] overlapped signal (multiplicity could not be determined)

Table S6. Comparison of the ^{13}C NMR spectroscopic data (151 MHz, $CDCl_3$) of the previously undescribed (20*S*,24*R*)-epoxy-25-hydroxy-A-homo-4-oxadammaran-3-one (**3**) and the known (20*S*,24*S*)-epoxy-25-hydroxy-A-homo-4-oxadammaran-3-one (**3a**) and (20*R*,24*S*)-epoxy-25-hydroxy-A-homo-4-oxadammaran-3-one (**3b**).

	**(20*S*,24*R*)-epoxy-25-hydroxy-A-homo-4-oxadammaran-3-one (3)** (in this study)	**(20*S*,24*S*)-epoxy-25-hydroxy-A-homo-4-oxadammaran-3-one (3a)** (Pan et al., 2010)	**(20*R*,24*S*)-epoxy-25-hydroxy-A-homo-4-oxadammaran-3-one (3b)** (Pan et al., 2010)
**Position**	**δ_C (ppm)**		
1	40.5	40.3	40.7
2	32.5	32.4	32.8
3	175.2	175.0	175.5
4	86.3	86.0	86.4
5	53.3	53.1	53.5
6	23.7	23.6	23.9
7	34.4	34.2	34.6
8	40.3	40.2	40.5
9	51.5	51.4	51.7
10	39.4	39.3	39.7
11	22.9	23.0	23.1
12	27.6	27.2	27.3
13	43.4	43.1	43.5
14	50.1	49.9	50.2
15	31.4	30.9	31.4
16	25.7	25.7	26.1
17	49.5	49.6	50.3
18	15.0	14.9	15.3
19	18.5	18.4	18.8
20	86.4	86.5	86.8
21	**23.6**	**27.2**	**22.1**
22	**36.0**	**34.8**	**37.8**
23	26.3	26.3	26.2
24	**83.5**	**86.4**	**84.9**
25	**71.6**	**70.2**	**71.5**
26	27.6	27.8	27.6
27	24.4	24.0	24.4
28	31.1	30.9	31.3
29	27.0	26.9	26.7
30	16.3	16.1	16.2

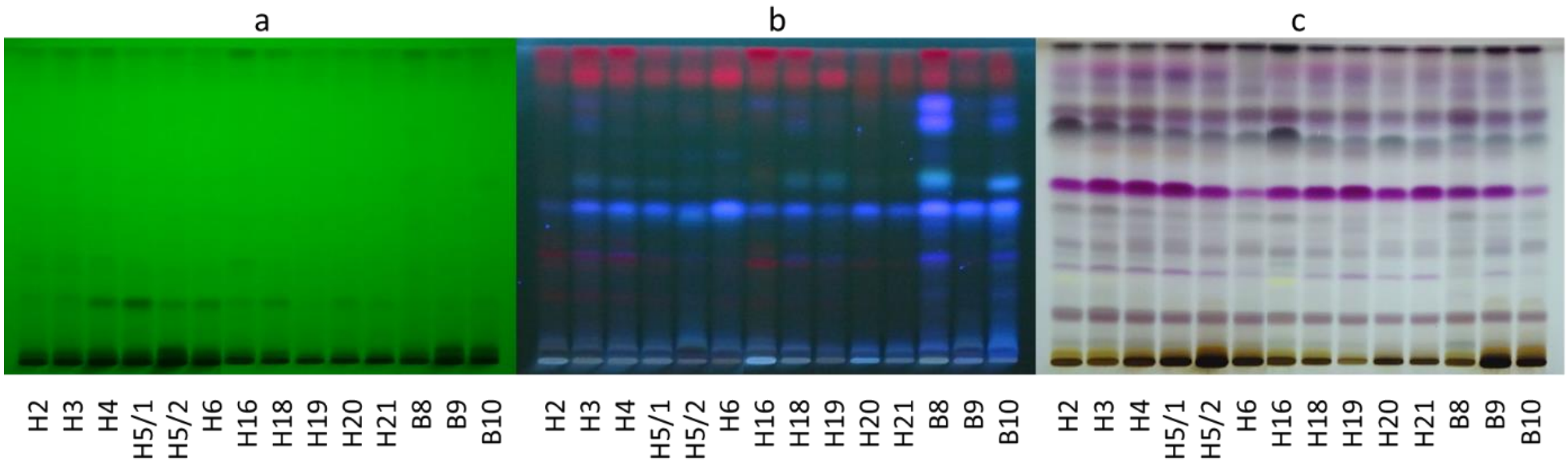

Figure S1. TLC chromatograms of the *A. altissima* petiole extracts (H2–H6, H16, H18–H21, B8–B10), developed with toluene–isopropyl acetate–methanol (5:4:1, *V/V*), before (at 254 nm (**a**) and 366 nm (**b**)) and after derivatization with *p*-anisaldehyde reagent under white light (**c**).

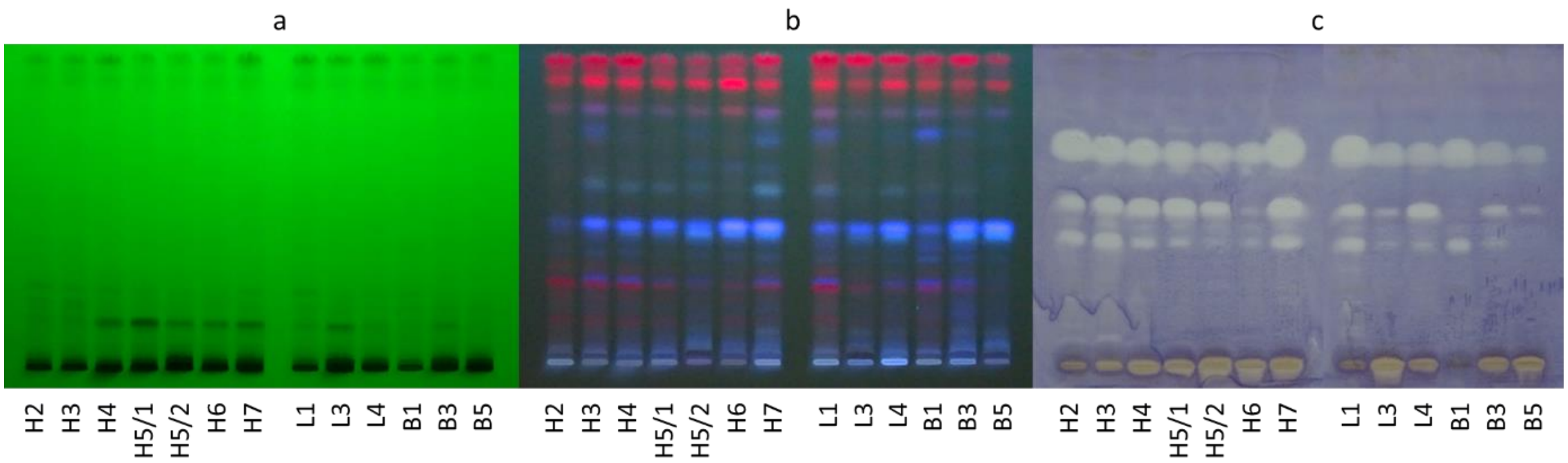

Figure S2. TLC chromatograms at 254 nm (**a**) and 366 nm (**b**), and TLC–*B. subtilis* bioautogram under white light (**c**) of the *A. altissima* petiole extracts (H2–H7, L1, L3, L4, B1, B3, B5), developed with toluene–isopropyl acetate–methanol (5:4:1, *V/V*).

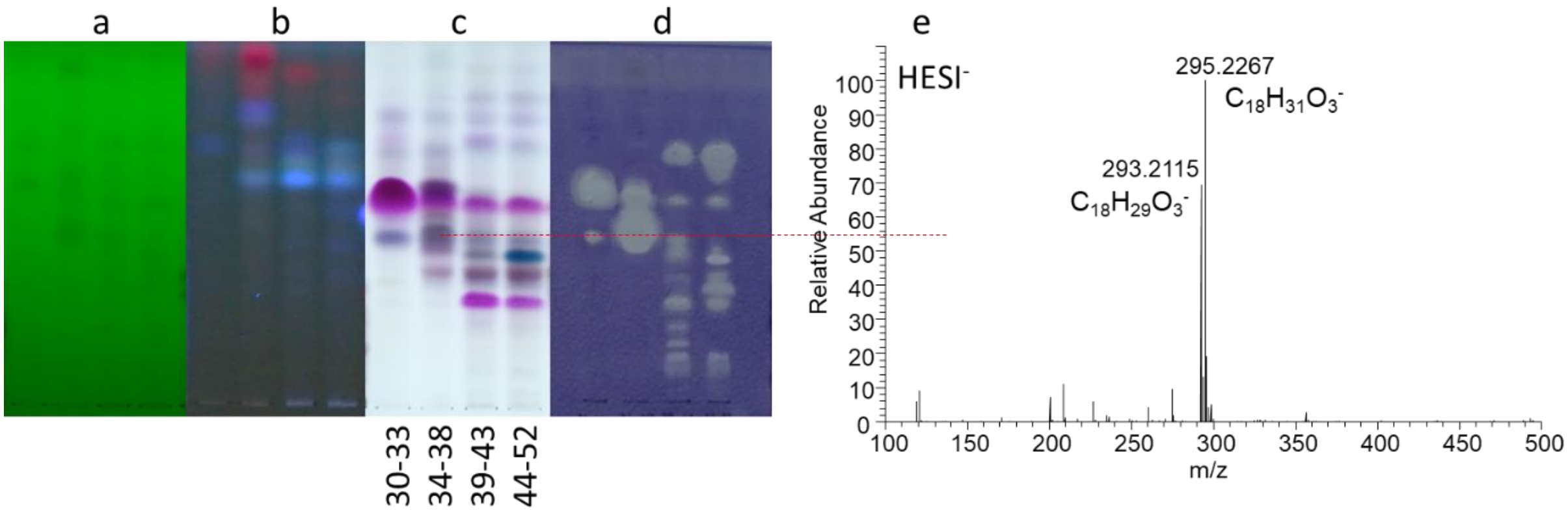

Figure S3. TLC chromatograms (**a**, **b**, **c**) and bioautogram (**d**) of the merged flash chromatography fractions (30–33, 34–38, 39–43, 44–52), developed with toluene–isopropyl acetate–methanol (5:4:1, *V/V*), detected before (at 254 nm (**a**) and 366 nm (**b**)) and after derivatization with *p*-anisaldehyde reagent under white light (**c**), and *B. subtilis* bioassay (**d**), as well as TLC–HESI$^-$-HRMS spectrum (**e**) recorded from the zones of compounds **4** and **5** (fraction 34–38) obtained via the elution head-based TLC-MS interface and background subtraction.

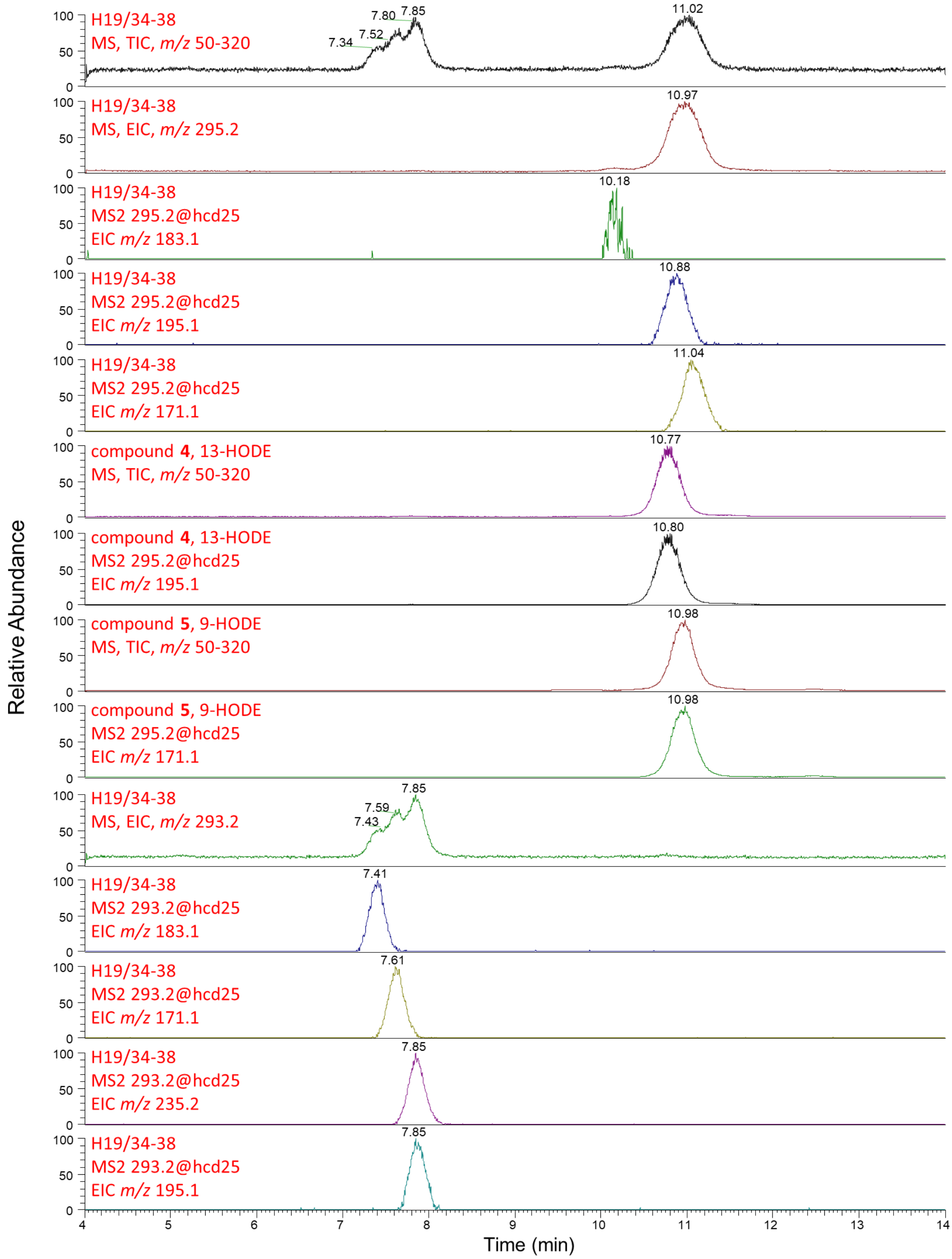

Figure S4. HPLC–HRMS(/MS) total ion chromatograms (TICs) and extracted ion chromatograms (EICs) of the flash chromatography fraction H19/34–38, and the compounds **4** and **5**.

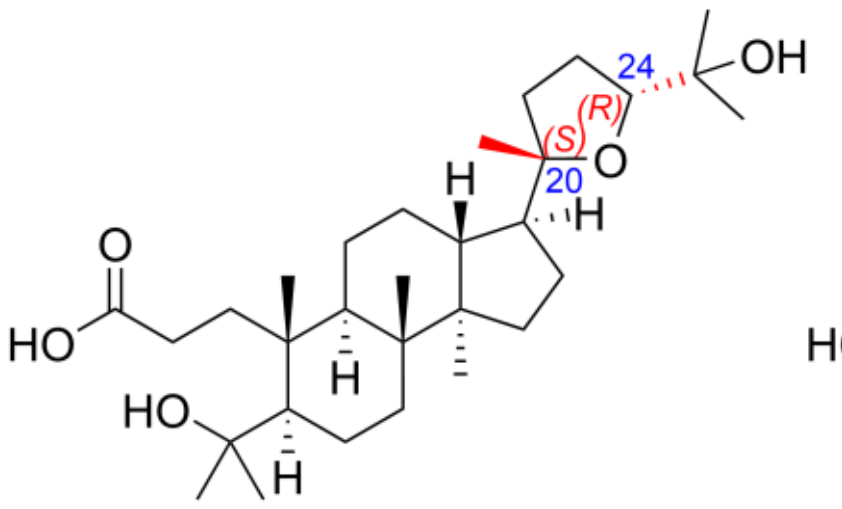

2

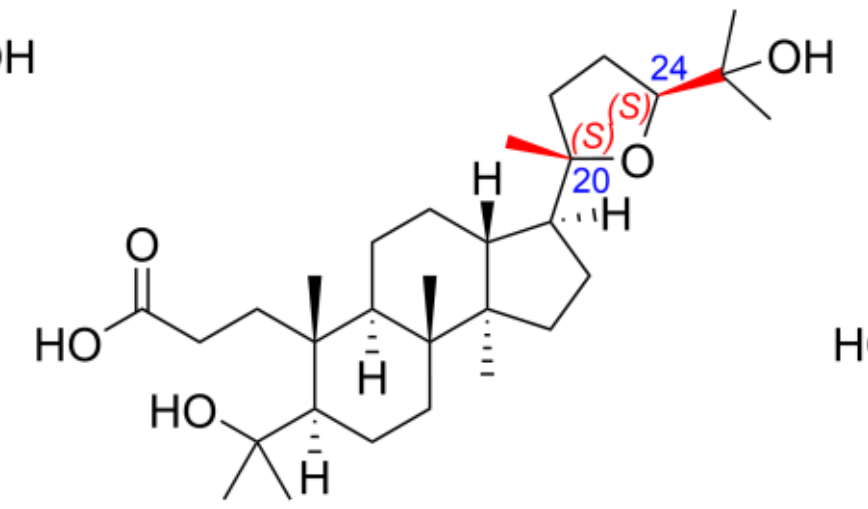

2a

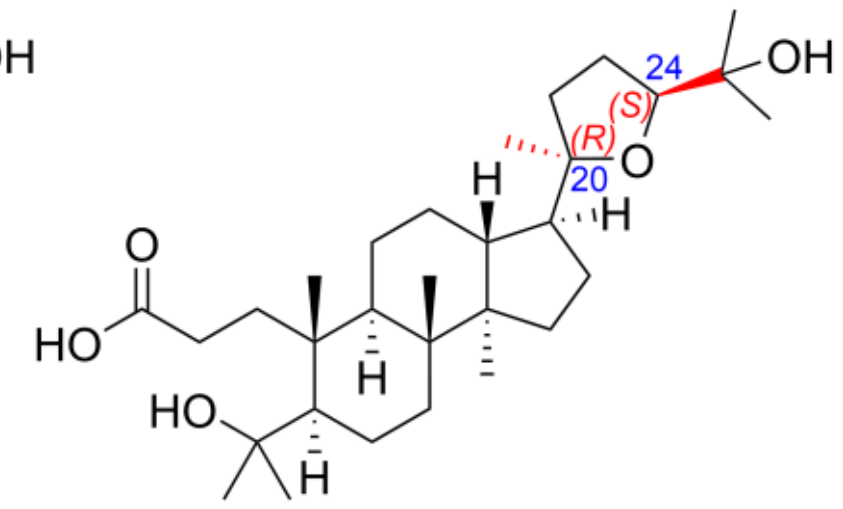

2b

Figure S5. Chemical structures of the previously undescribed foveolin C (**2**) (20*S*,24*R* isomer) and the known foveolin A (**2a**) (20*S*,24*S* isomer) and foveolin B (**2b**) (20*R*,24*S* isomer).

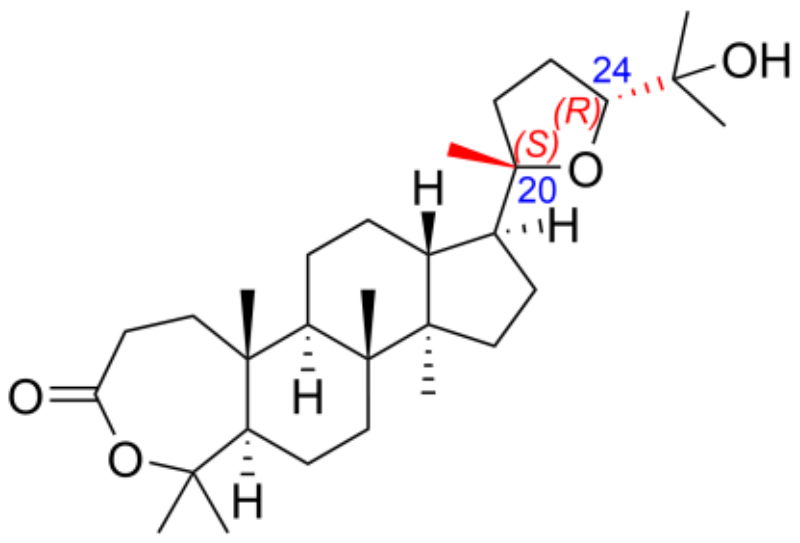

3

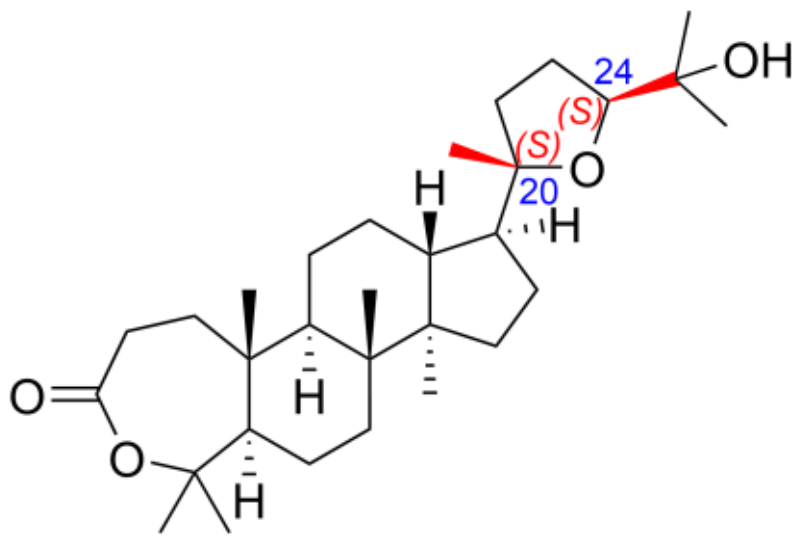

3a

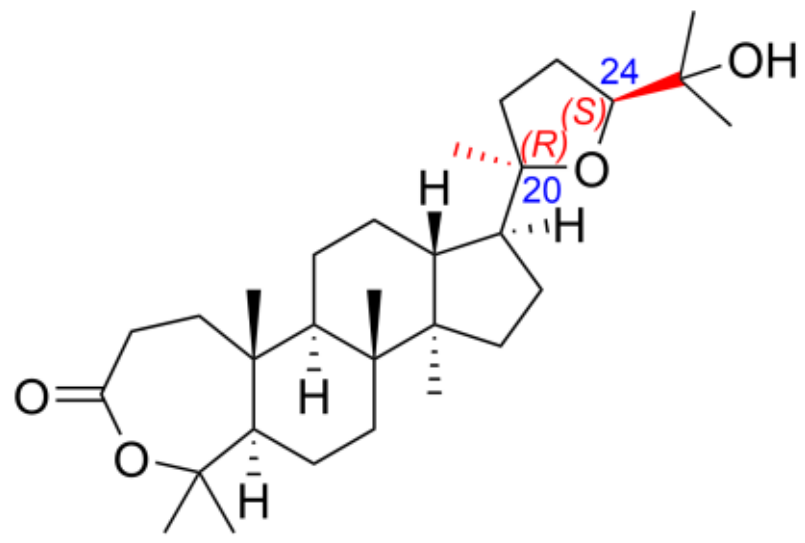

3b

Figure S6. Chemical structures of the previously undescribed (20*S*,24*R*)-epoxy-25-hydroxy-A-homo-4-oxadammaran-3-one (**3**) (20*S*,24*R* isomer) and the known (20*S*,24*S*)-epoxy-25-hydroxy-A-homo-4-oxadammaran-3-one (**3a**) (20*S*,24*S* isomer) and (20*R*,24*S*)-epoxy-25-hydroxy-A-homo-4-oxadammaran-3-one (**3b**) (20*R*,24*S* isomer).

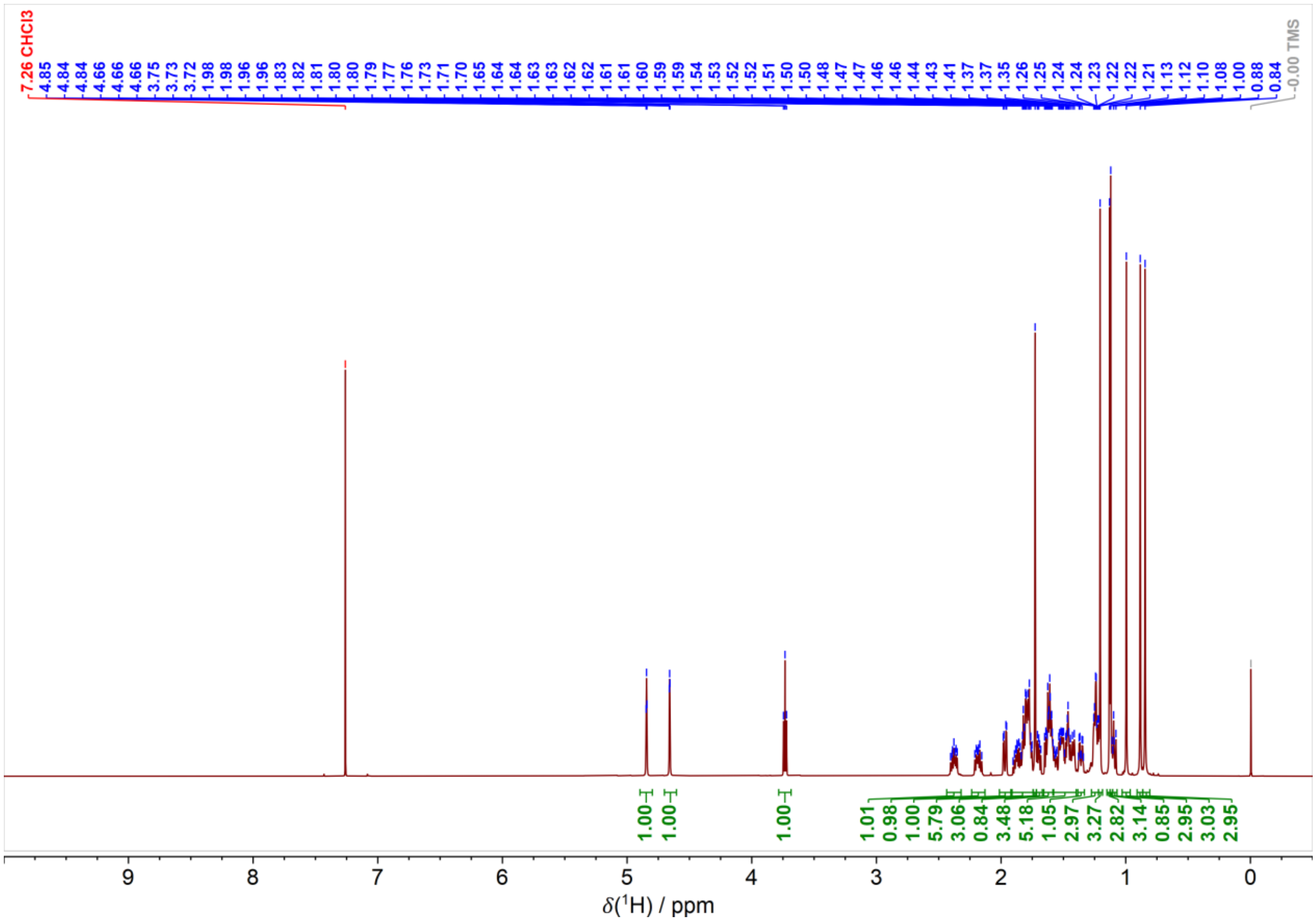

Figure S7. ^{1}H NMR spectrum of shoreic acid (**1**) (600 MHz, $CDCl_3$).

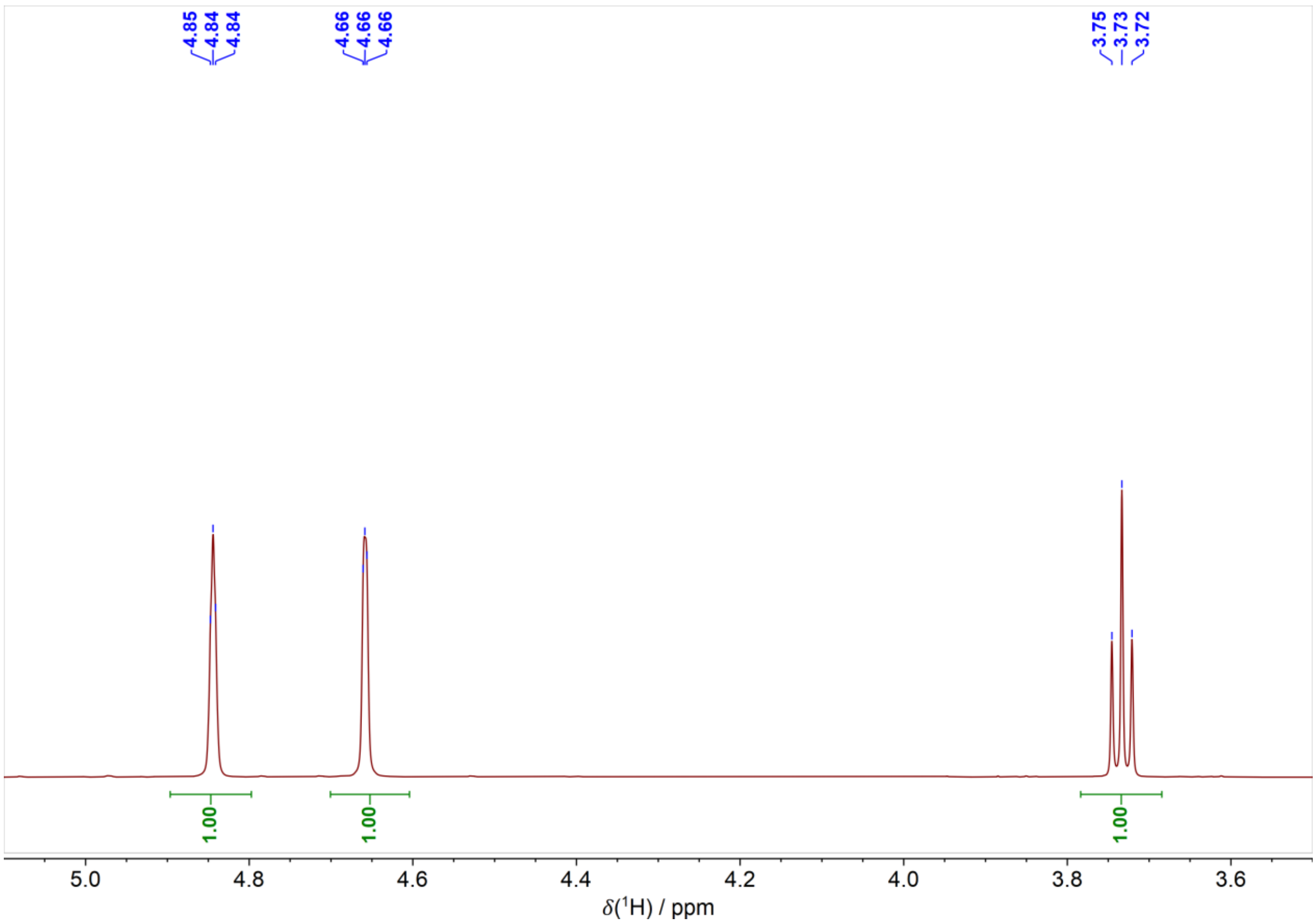

Figure S8. ^{1}H NMR spectrum of shoreic acid (**1**) (600 MHz, $CDCl_3$) – expansion (δ_H 5.1–3.5).

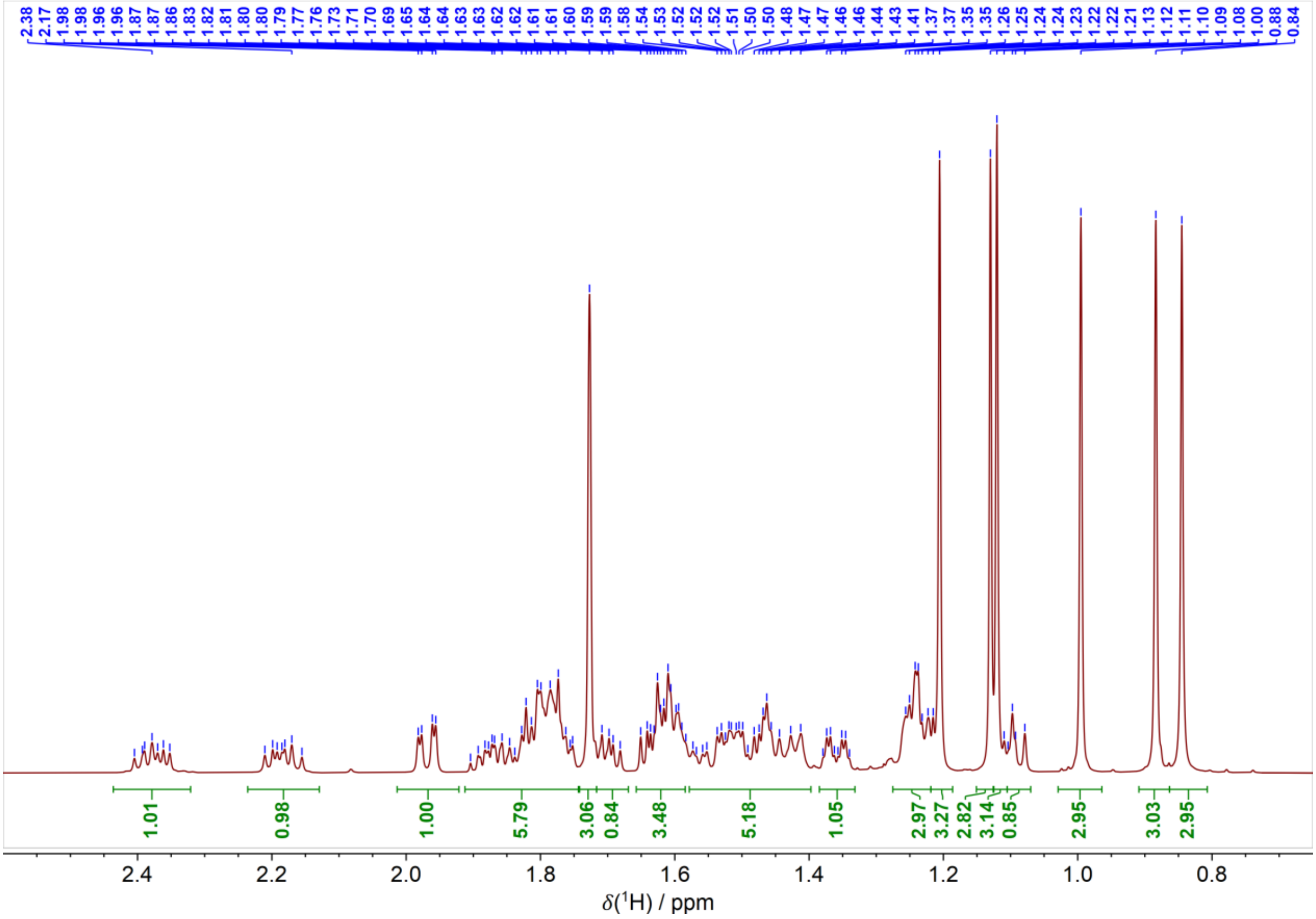

Figure S9. ^{1}H NMR spectrum of shoreic acid (**1**) (600 MHz, $CDCl_3$) – expansion (δ_H 2.6–0.65).

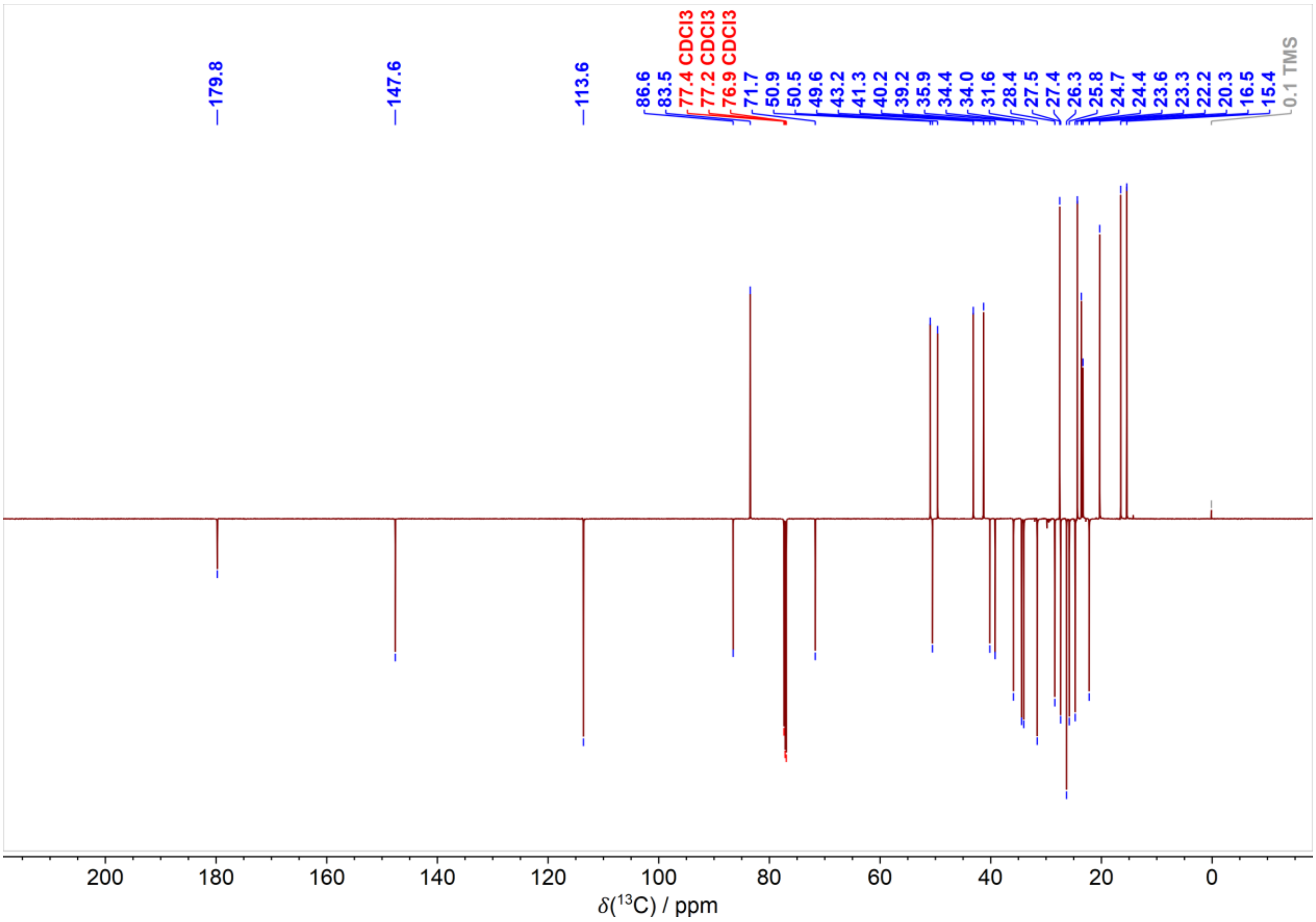

Figure S10. ^{13}C DEPTQ NMR spectrum of shoreic acid (**1**) (151 MHz, $CDCl_3$).

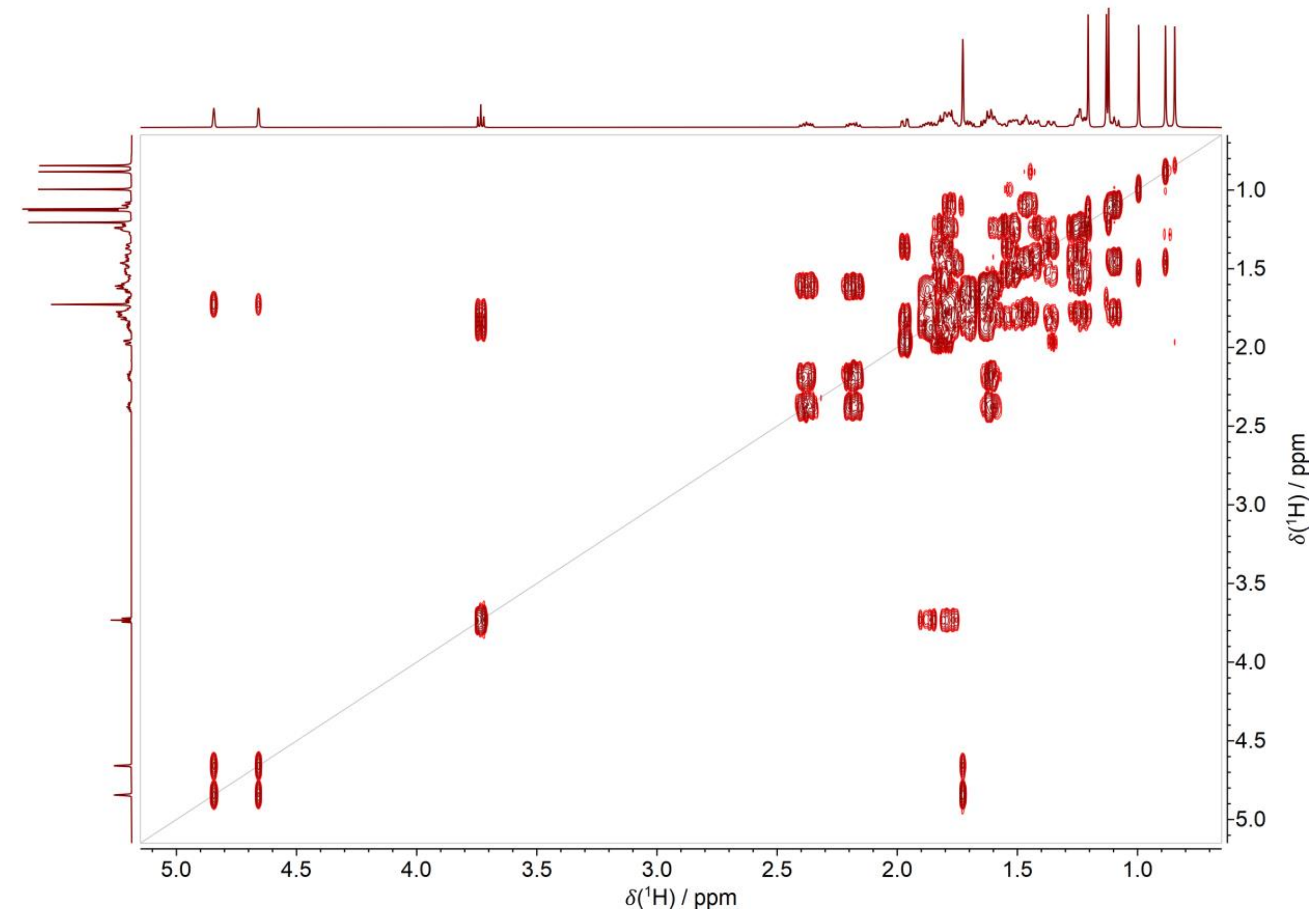

Figure S11. ^{1}H–^{1}H COSY NMR spectrum of shoreic acid (**1**) (600 MHz, $CDCl_3$).

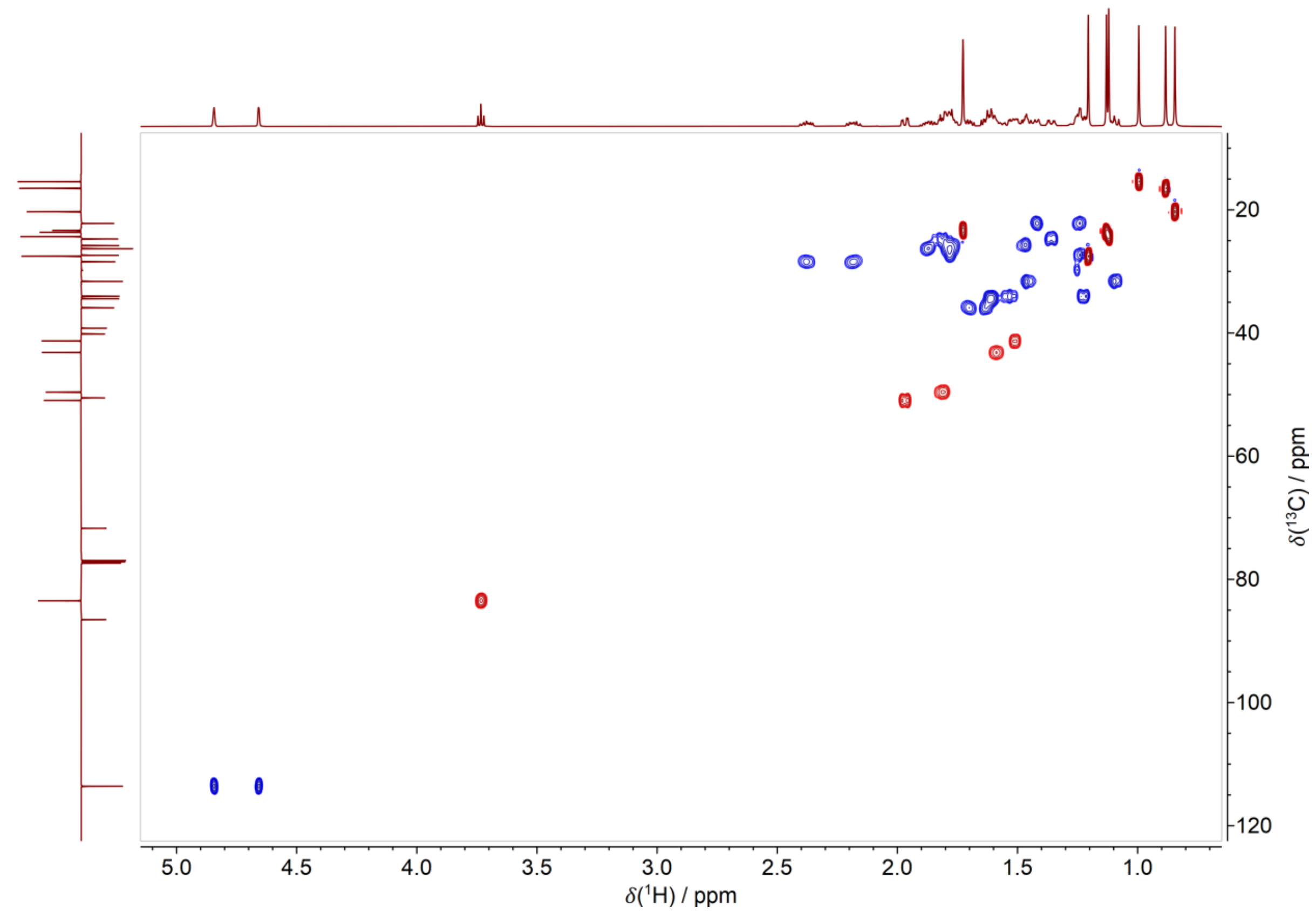

Figure S12. ^{1}H–^{13}C multiplicity-edited HSQC NMR spectrum of shoreic acid (**1**) (600/151 MHz, $CDCl_3$).

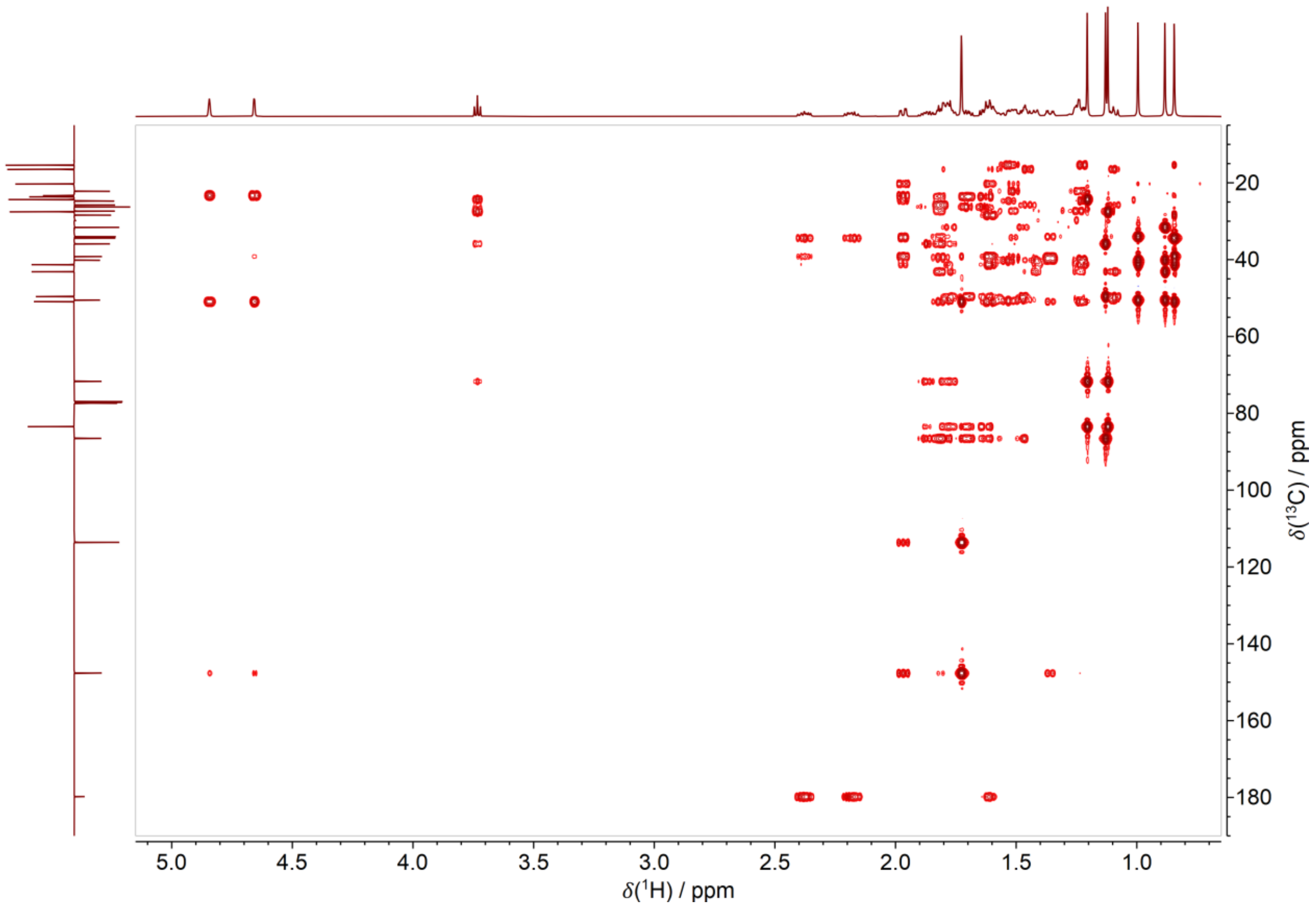

Figure S13. ^{1}H–^{13}C HMBC NMR spectrum of shoreic acid (**1**) (600/151 MHz, $CDCl_3$).

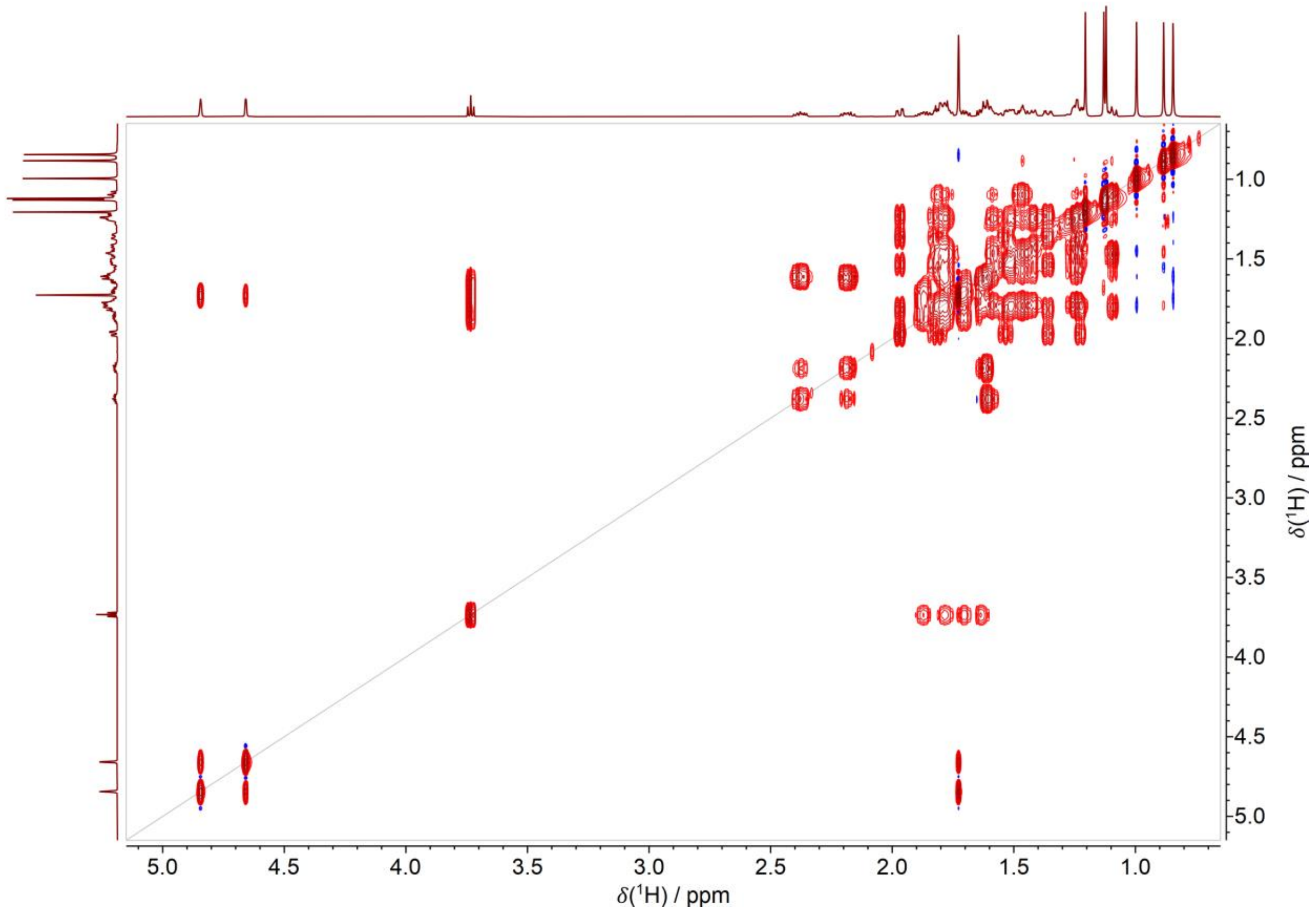

Figure S14. ^{1}H–^{1}H TOCSY NMR spectrum of shoreic acid (**1**) (600 MHz, $CDCl_3$).

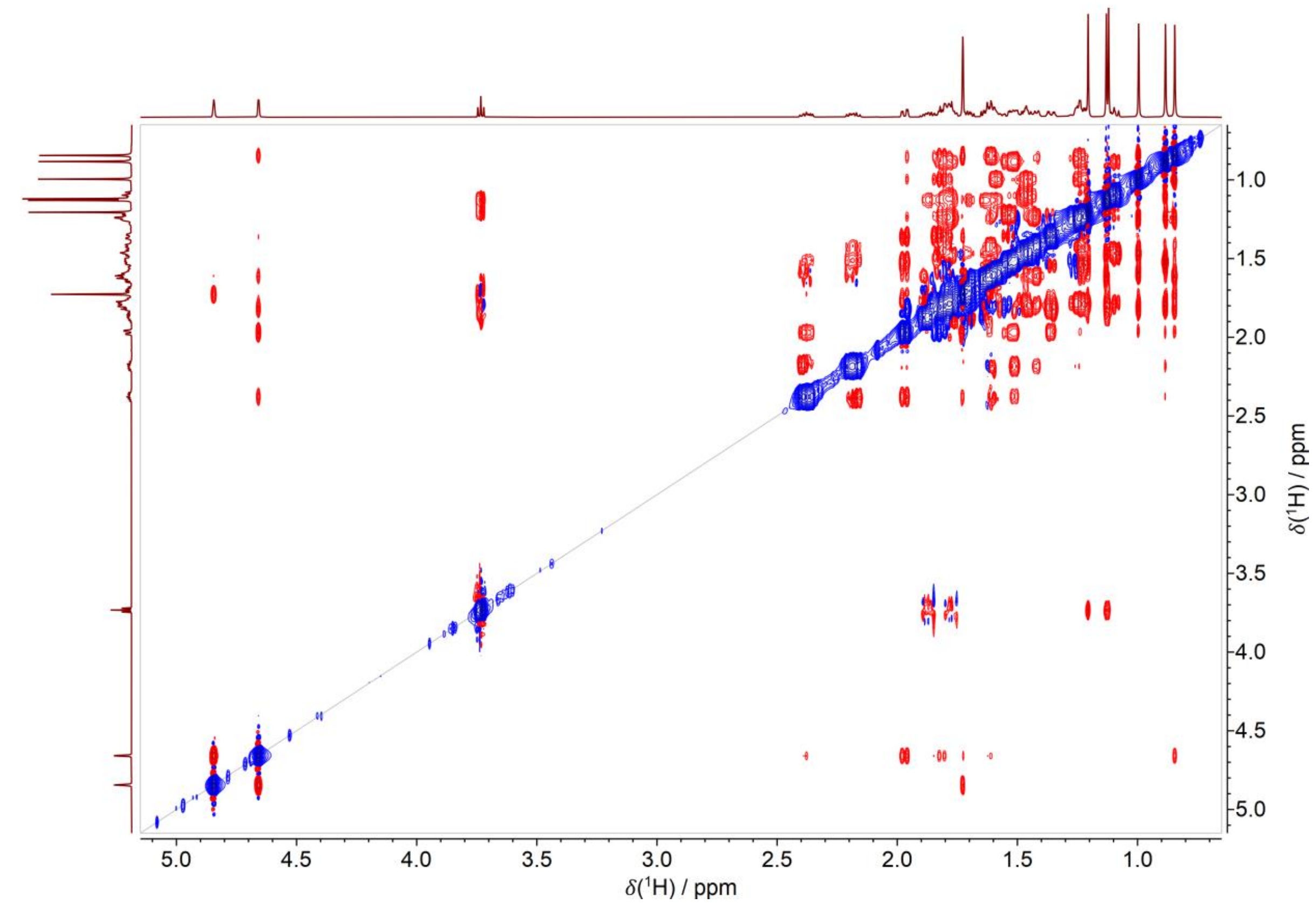

Figure S15. ^{1}H–^{1}H ROESY NMR spectrum of shoreic acid (**1**) (600 MHz, $CDCl_3$).

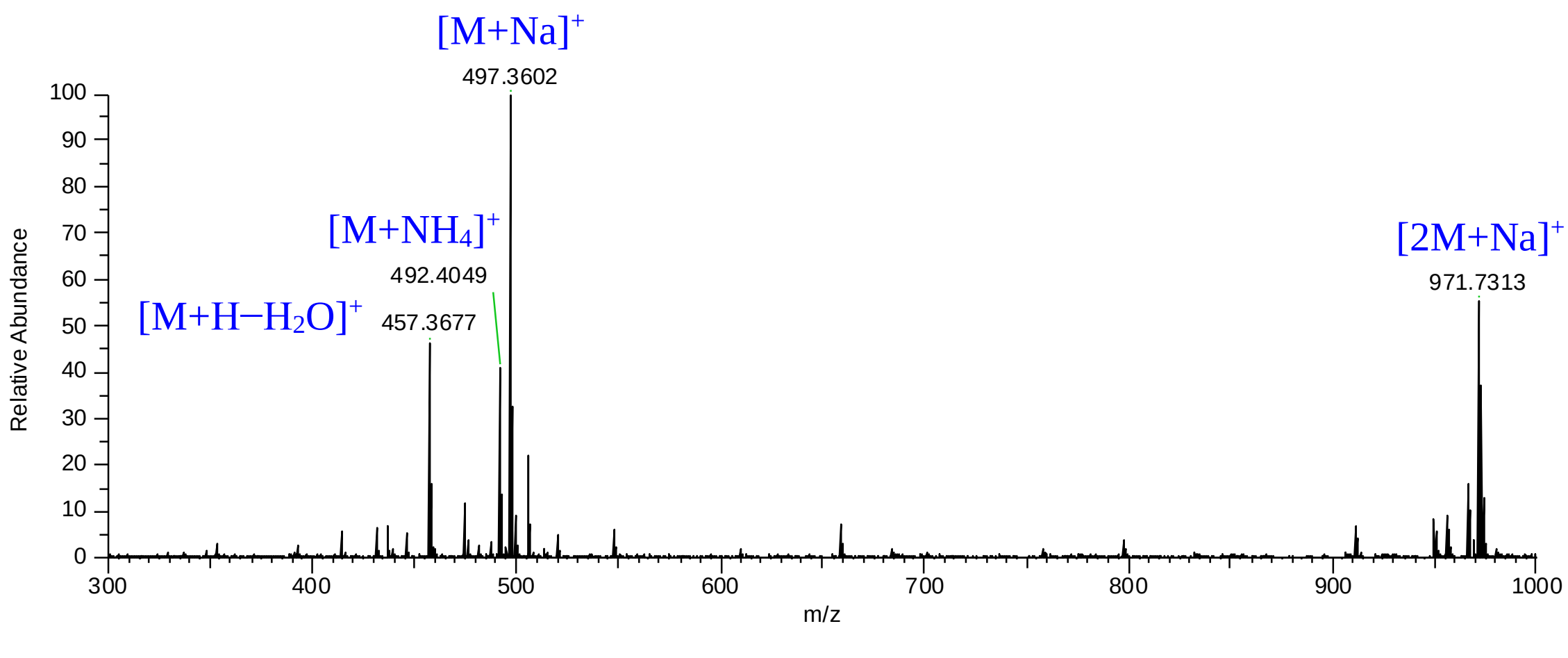

Figure S16. HR-ESI^{+}-MS spectrum of shoreic acid (**1**), *m*/*z* 497.3602 $[M+Na]^+$ (calculated for $C_{30}H_{50}O_4Na^+$, *m*/*z* 497.3601 $[M+Na]^+$, error: +0.1 ppm).

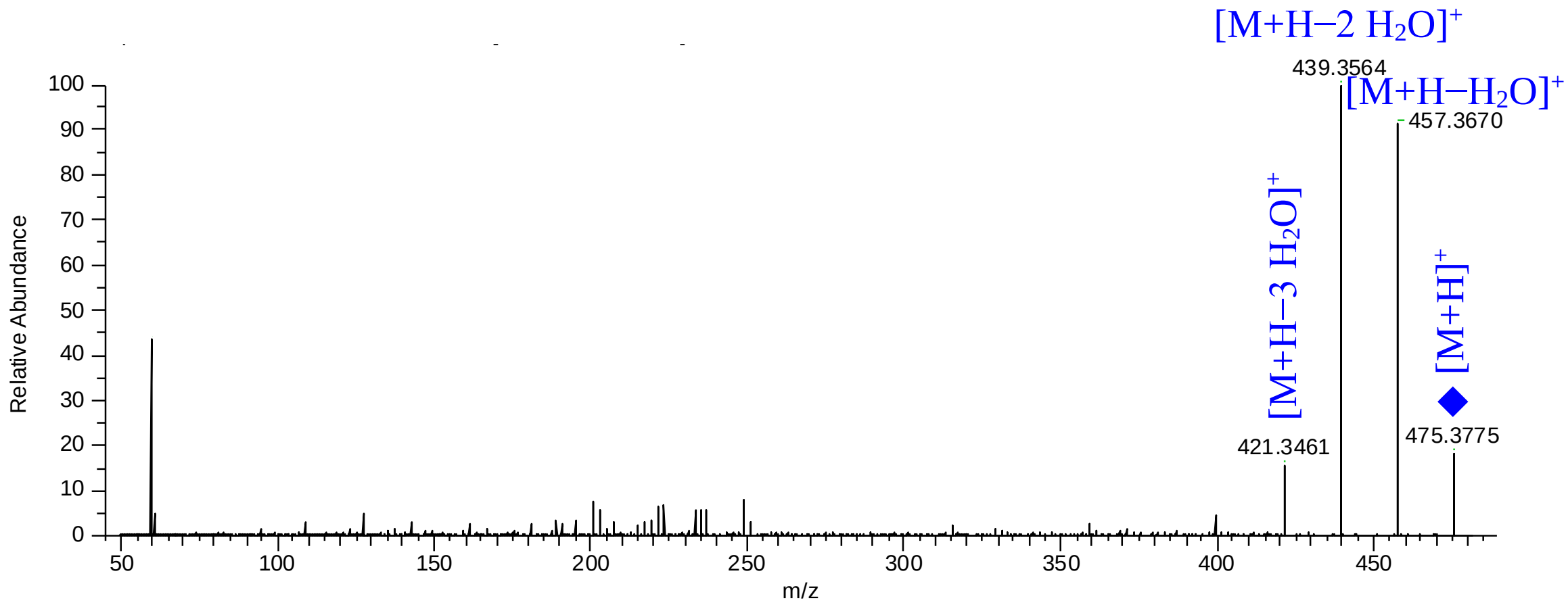

Figure S17. HR-ESI[+]-MS/MS spectrum of shoreic acid (**1**) with a normalized HCD collision energy of 15%. Precursor ion: *m/z* 475.3775 $[M+H]^+$, $C_{30}H_{51}O_4^+$.

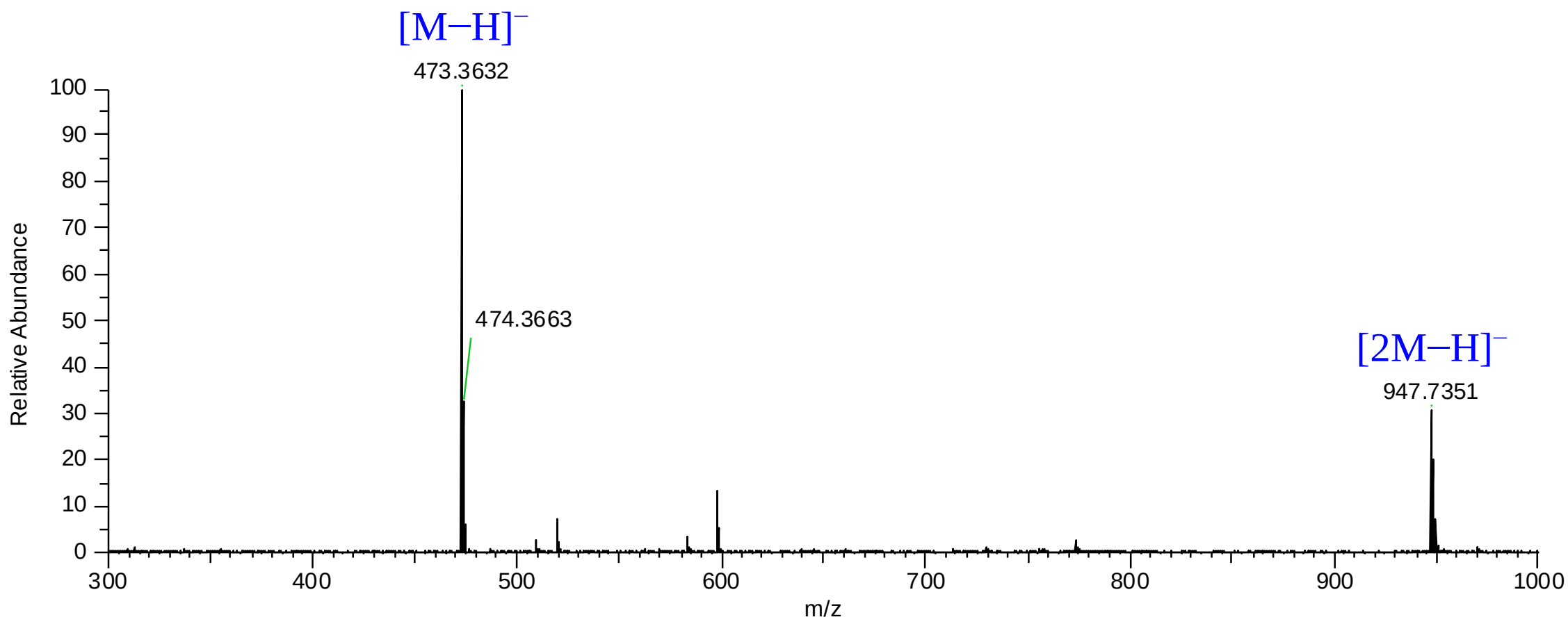

Figure S18. HR-ESI[−]-MS spectrum of shoreic acid (**1**), *m/z* 473.3632 $[M-H]^-$ (calculated for $C_{30}H_{49}O_4^-$, *m/z* 473.3636 $[M-H]^-$, error: −0.8 ppm).

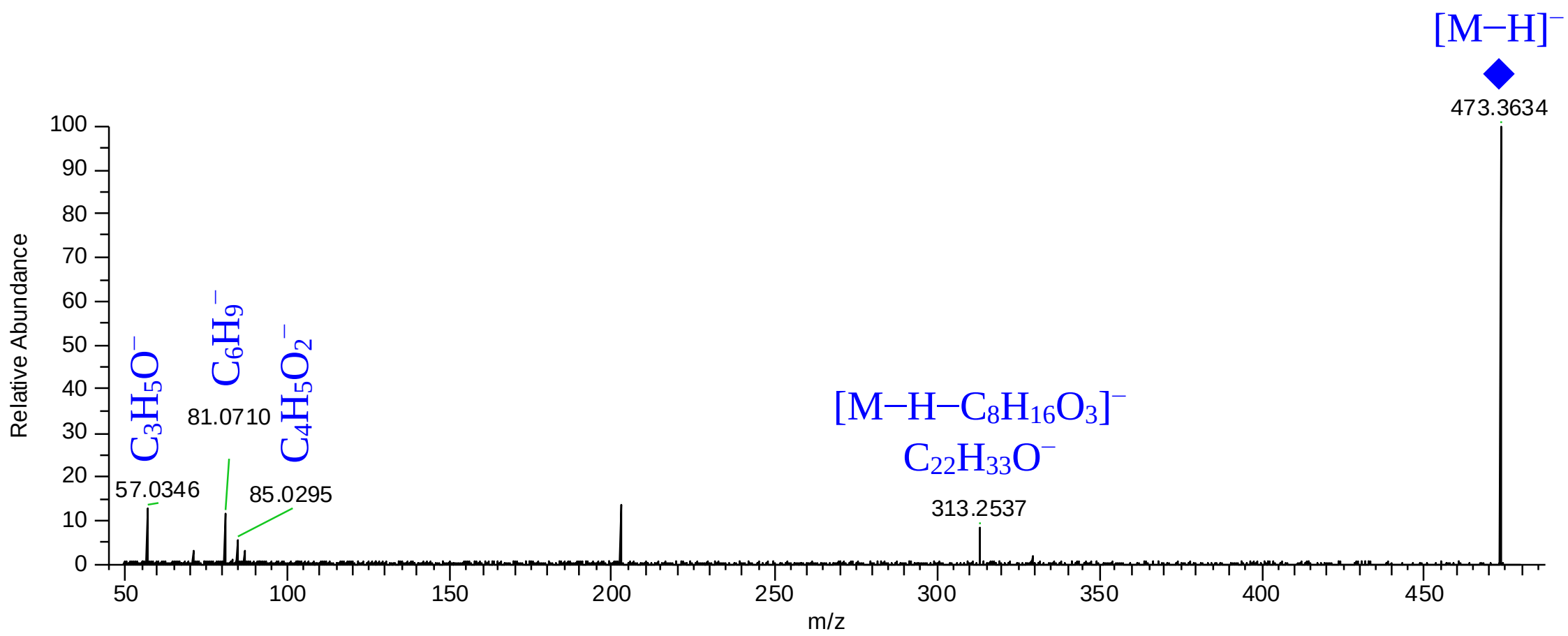

Figure S19. HR-ESI[−]-MS/MS spectrum of shoreic acid (**1**) with a normalized HCD collision energy of 60%. Precursor ion: *m/z* 473.3634 $[M-H]^-$, $C_{30}H_{49}O_4^-$.

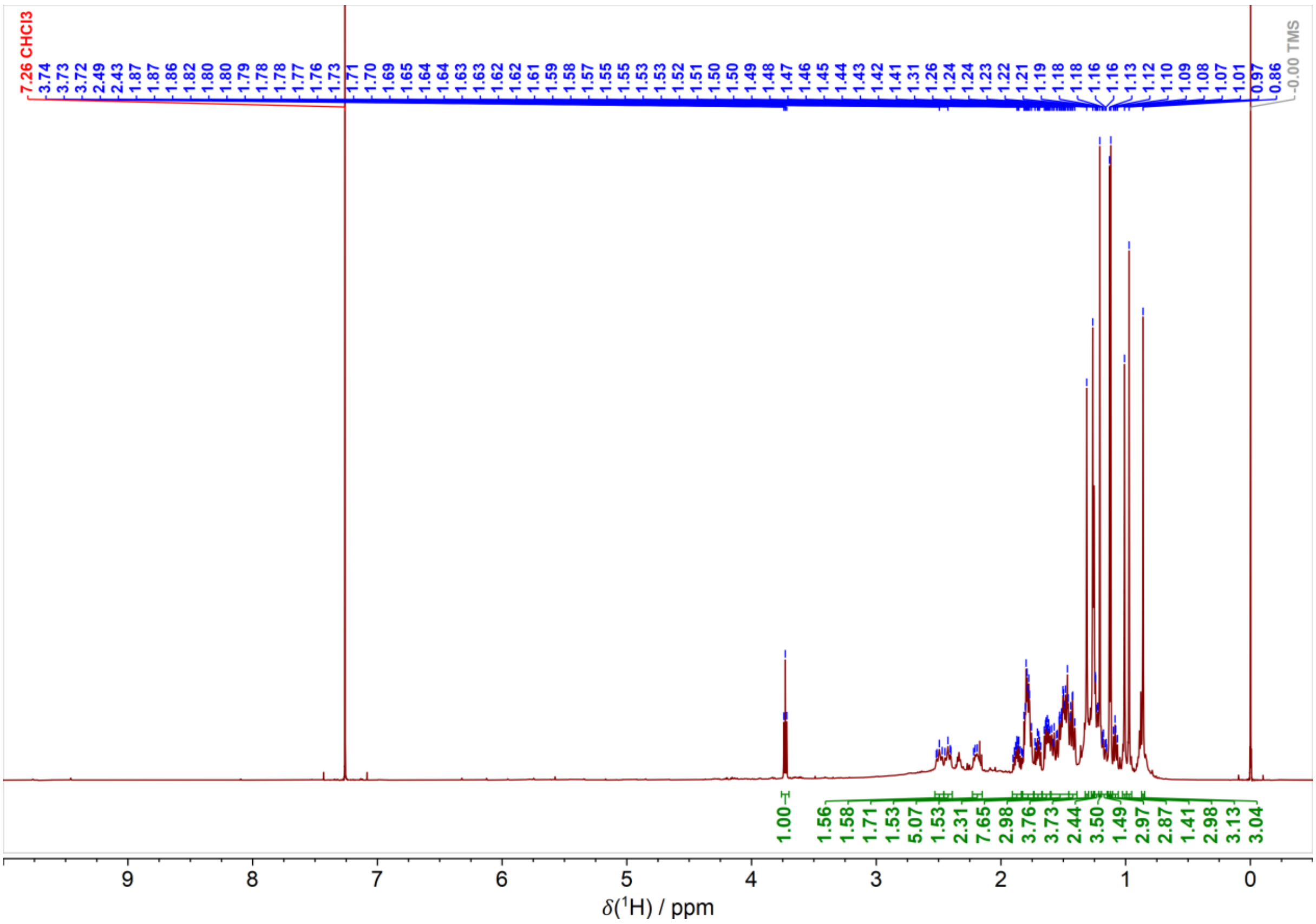

Figure S20. ^{1}H NMR spectrum of foveolin C (**2**) (600 MHz, $CDCl_3$).

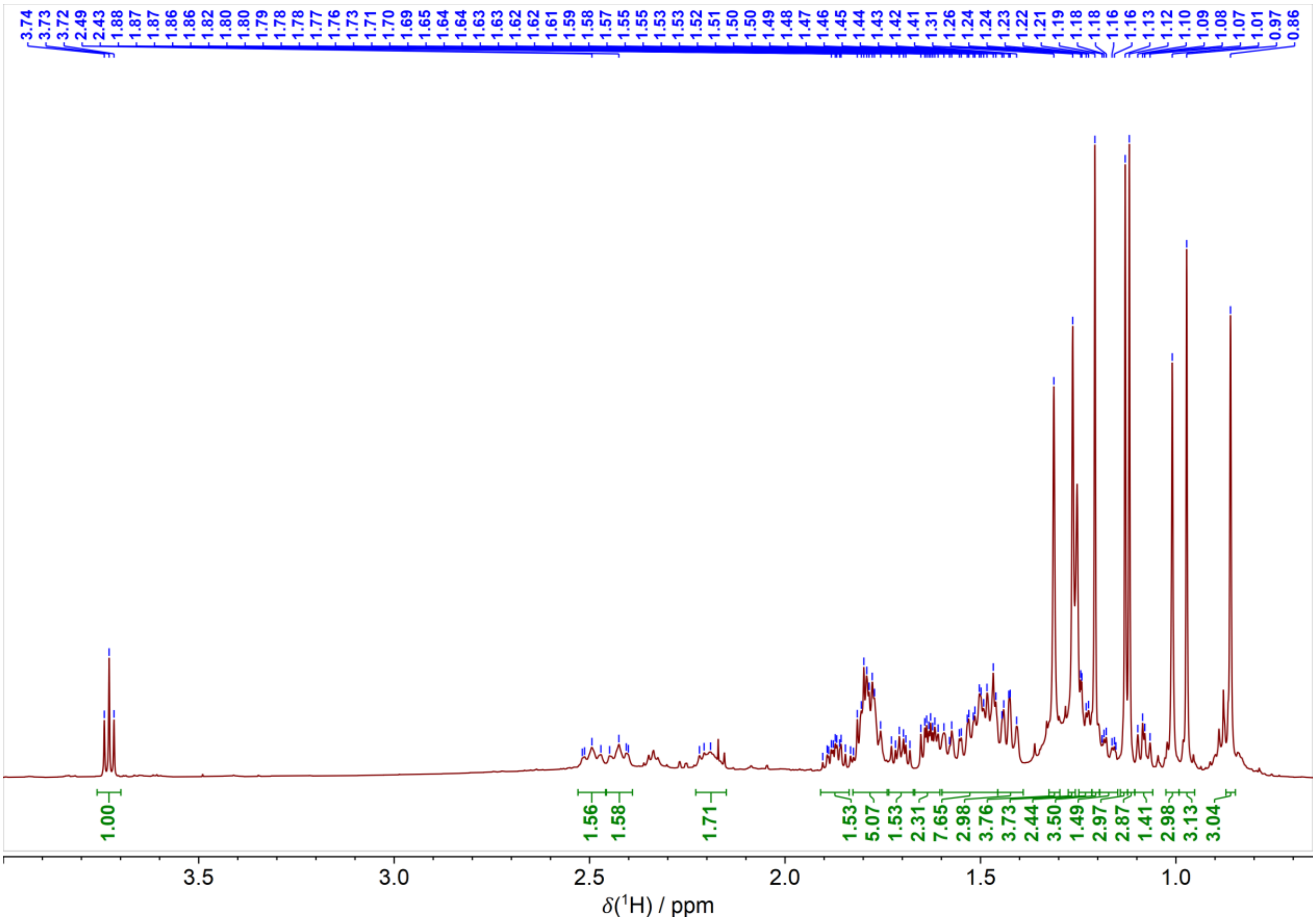

Figure S21. ^{1}H NMR spectrum of foveolin C (**2**) (600 MHz, $CDCl_3$) – expansion (δ_H 4.0–0.65).

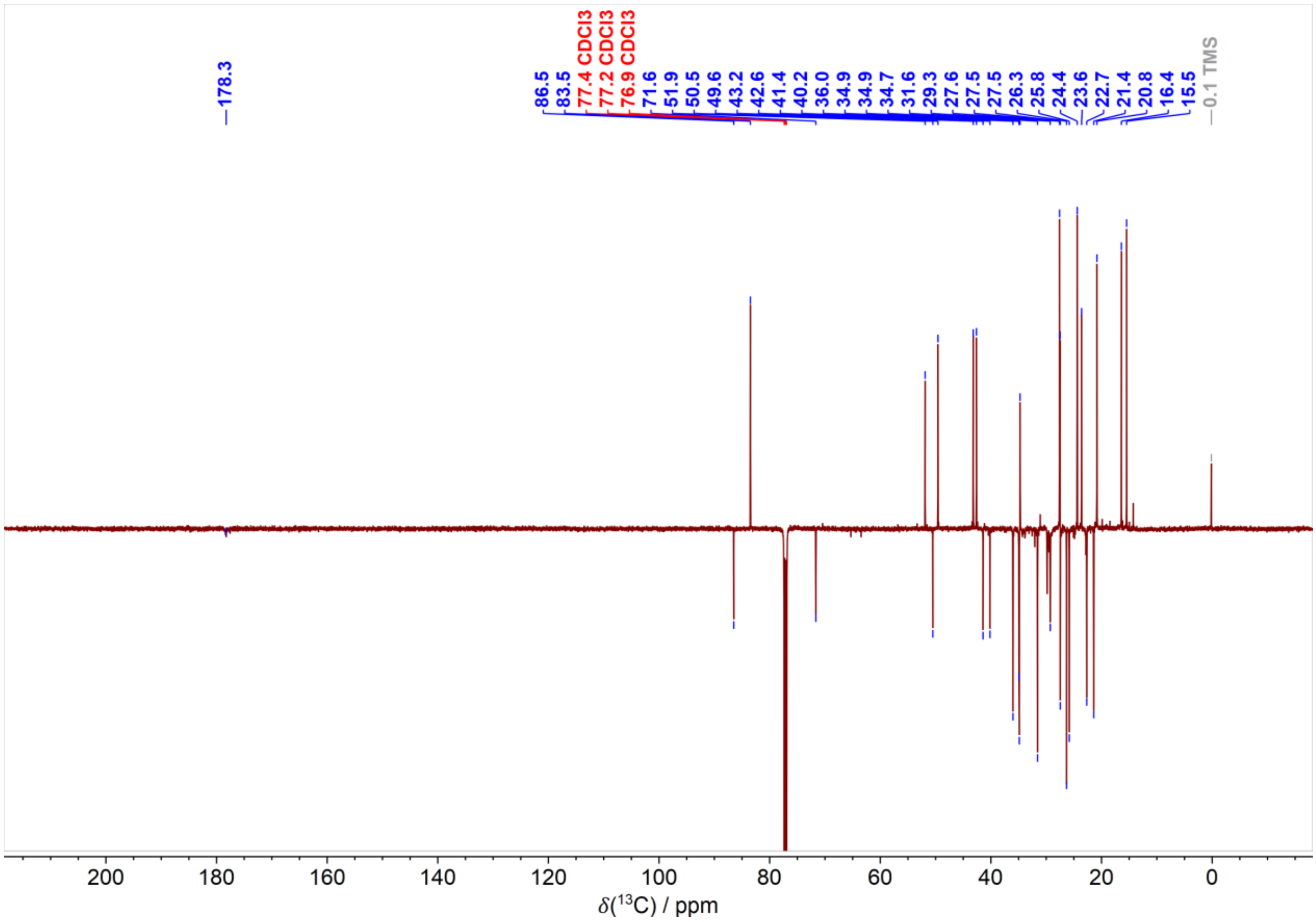

Figure S22. ^{13}C DEPTQ NMR spectrum of foveolin C (**2**) (151 MHz, $CDCl_3$).

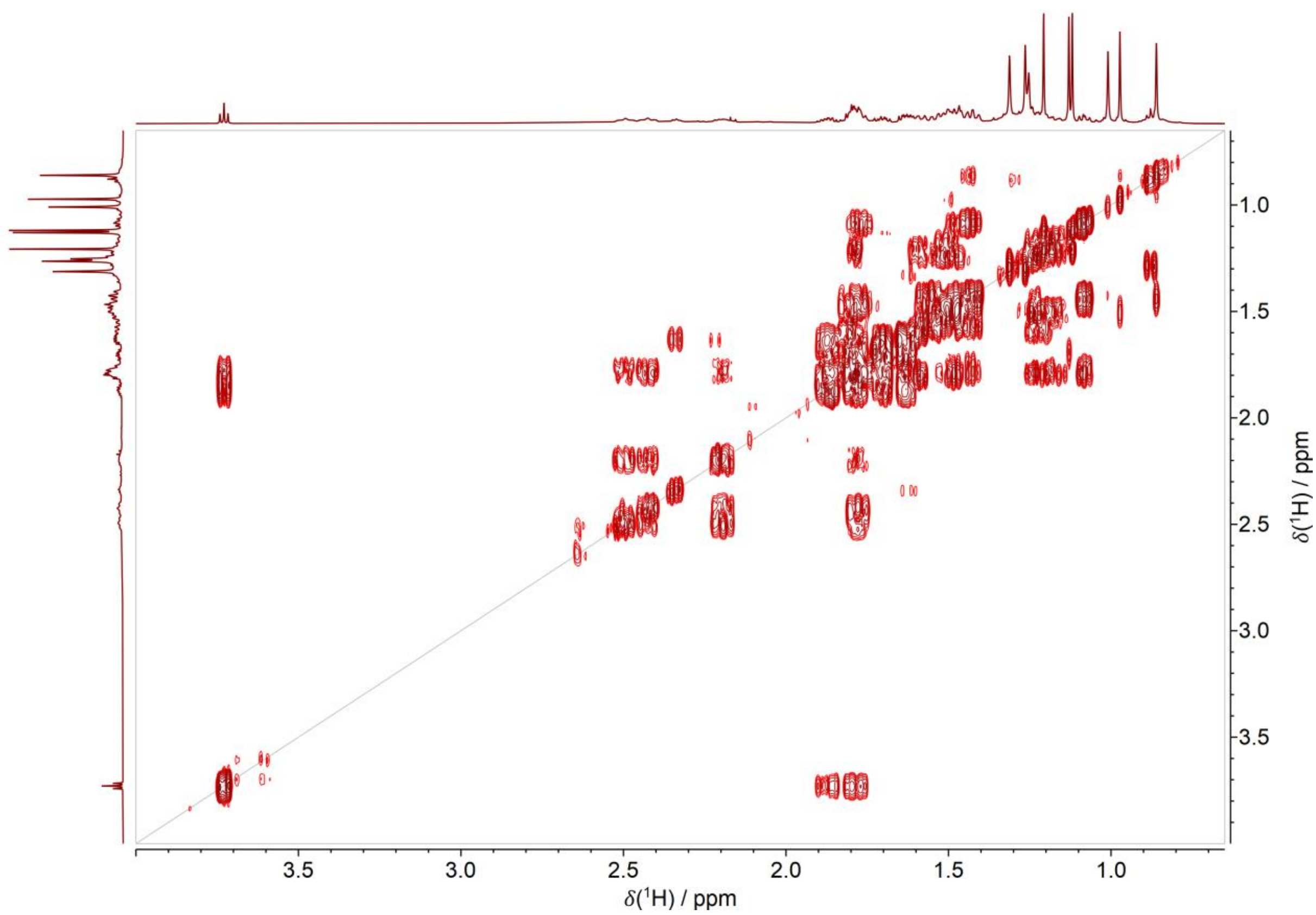

Figure S23. ^{1}H–^{1}H COSY NMR spectrum of foveolin C (**2**) (600 MHz, $CDCl_3$).

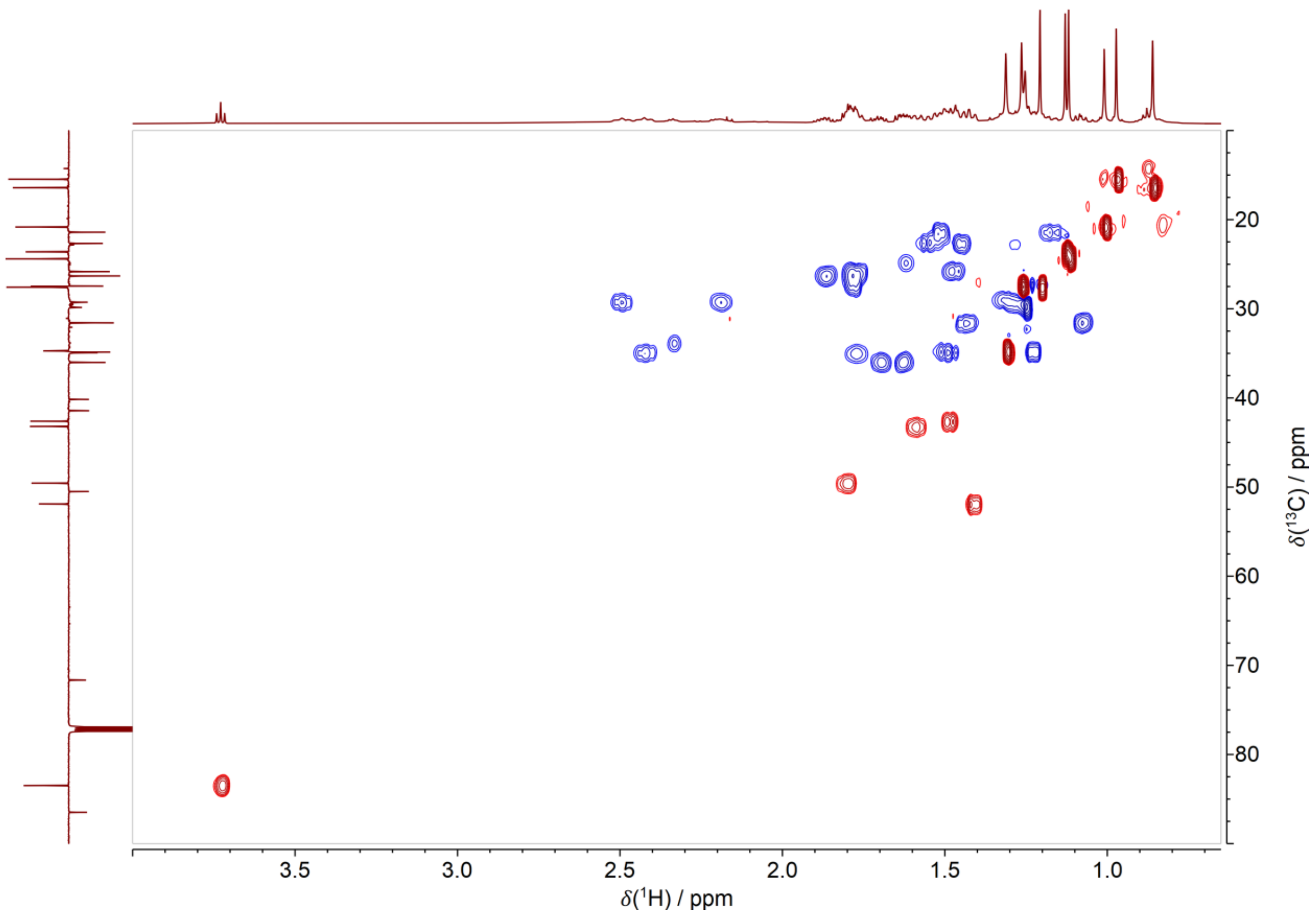

Figure S24. ^{1}H–^{13}C multiplicity-edited HSQC NMR spectrum of foveolin C (**2**) (600/151 MHz, $CDCl_3$).

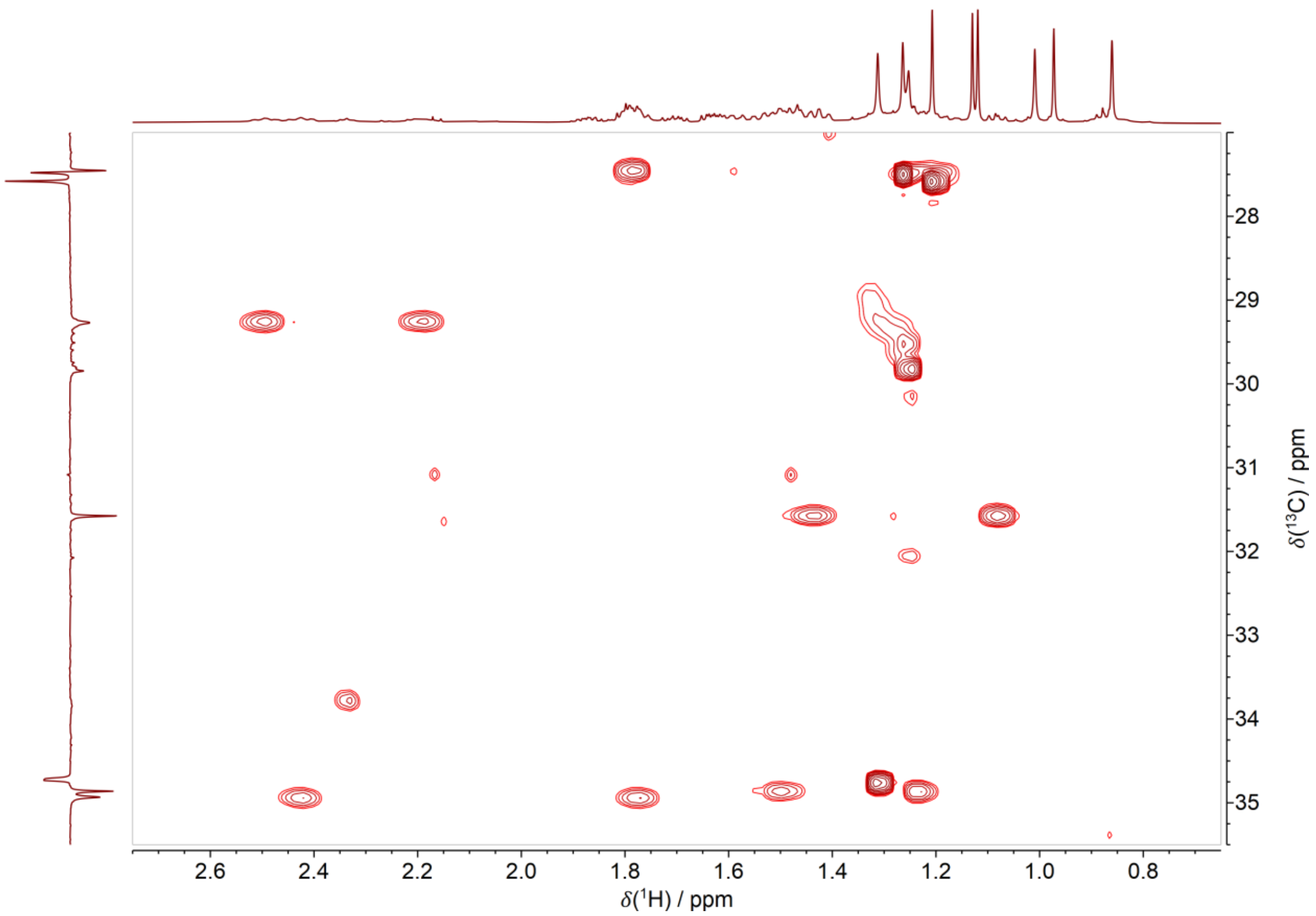

Figure S25. ^{1}H–^{13}C band-selective HSQC NMR spectrum of foveolin C (**2**) (600/151 MHz, $CDCl_3$) – aliphatic region (δ_C 35.5–27.0).

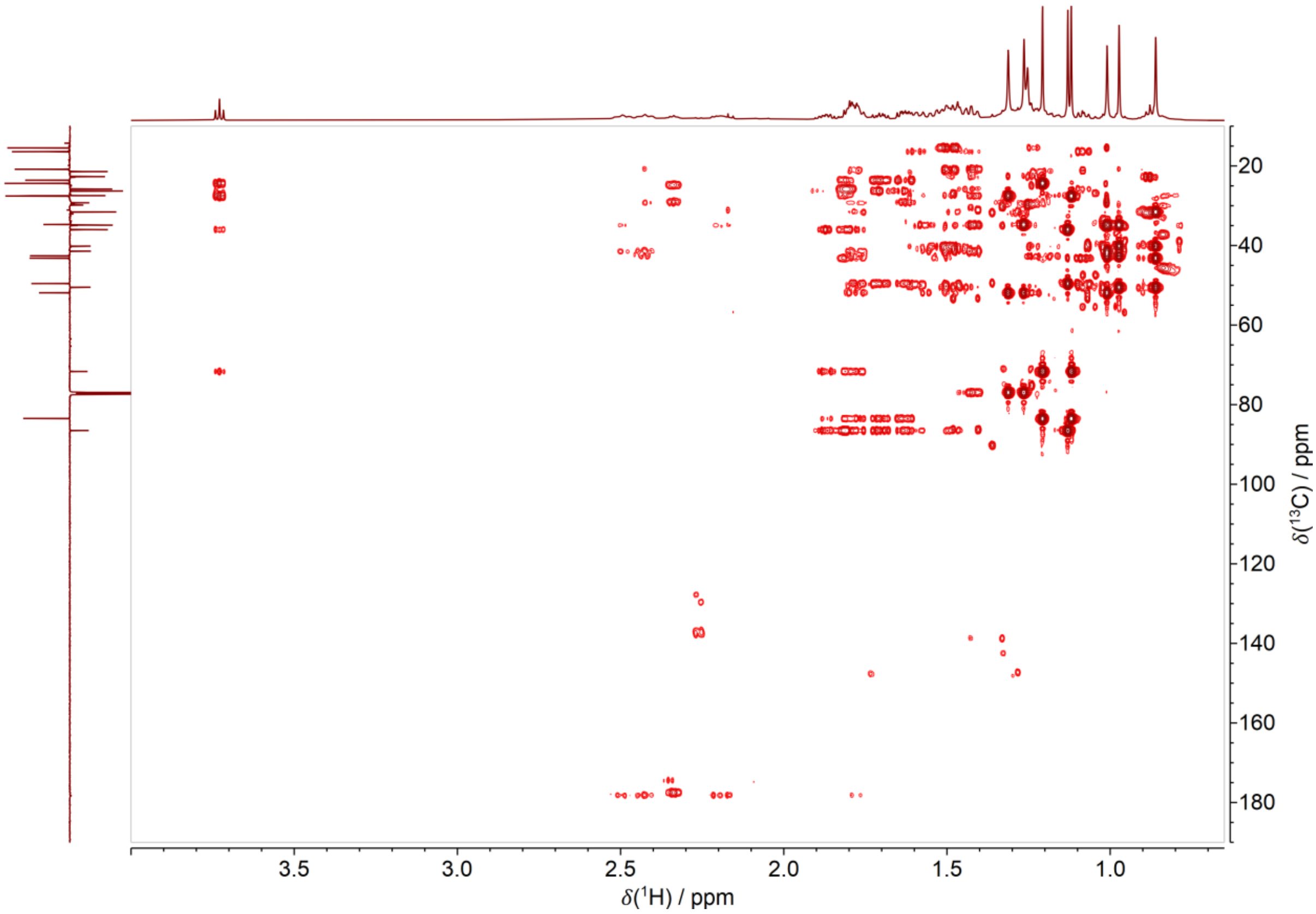

Figure S26. ^{1}H–^{13}C HMBC NMR spectrum of foveolin C (**2**) (600/151 MHz, $CDCl_3$).

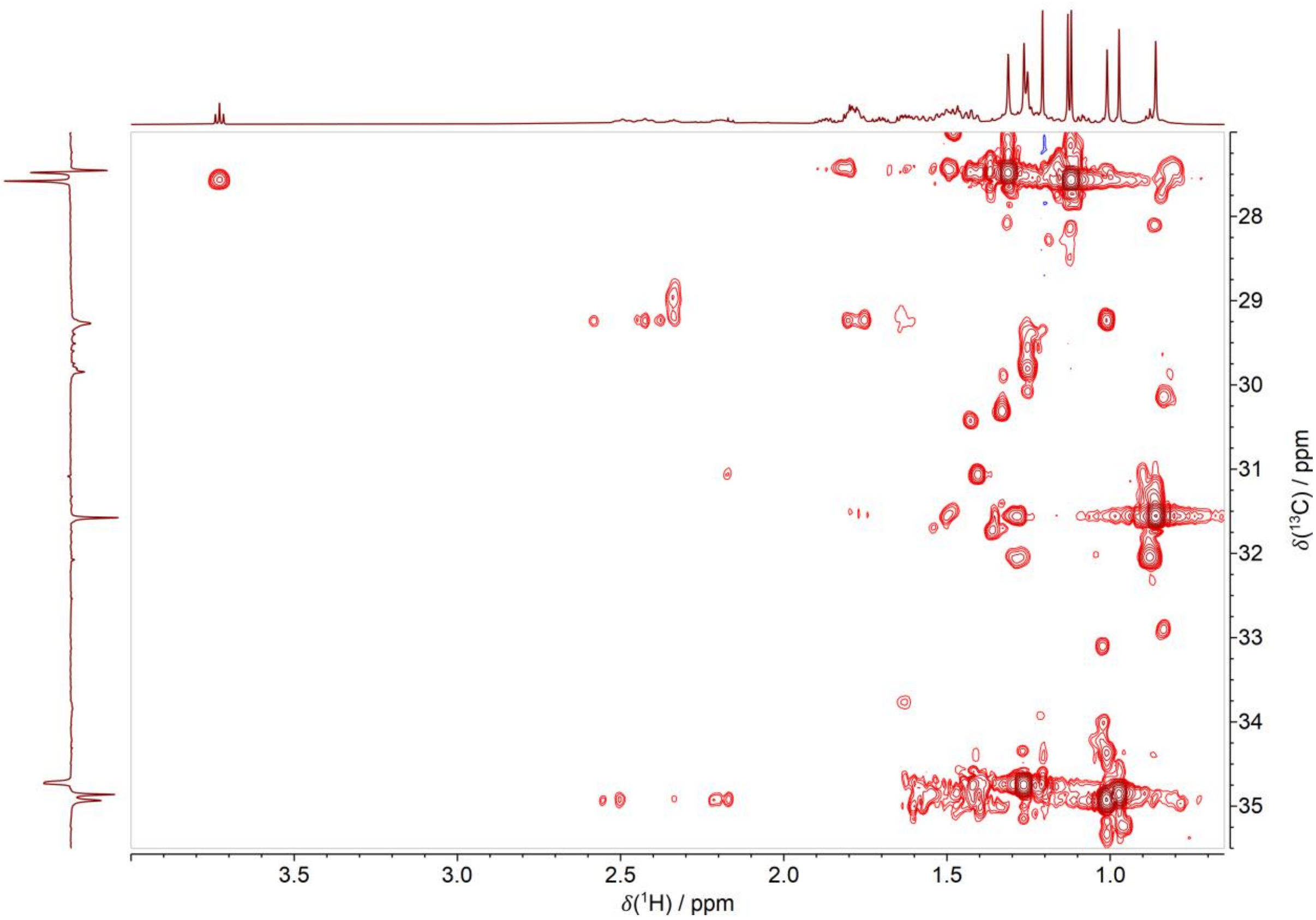

Figure S27. ^{1}H–^{13}C band-selective HMBC NMR spectrum of foveolin C (**2**) (600/151 MHz, $CDCl_3$) – aliphatic region (δ_C 35.5–27.0).

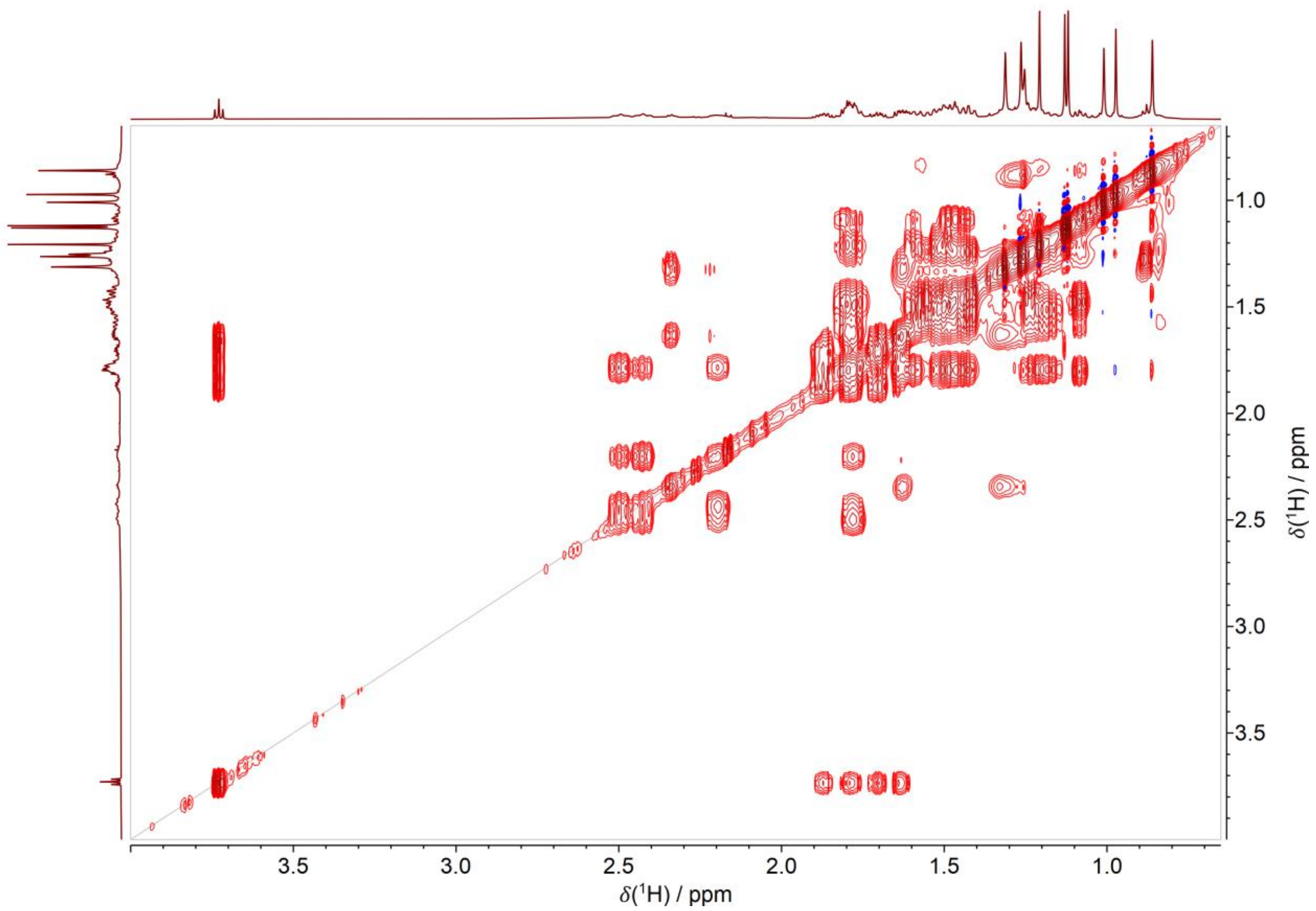

Figure S28. ^{1}H–^{1}H TOCSY NMR spectrum of foveolin C (**2**) (600 MHz, $CDCl_3$).

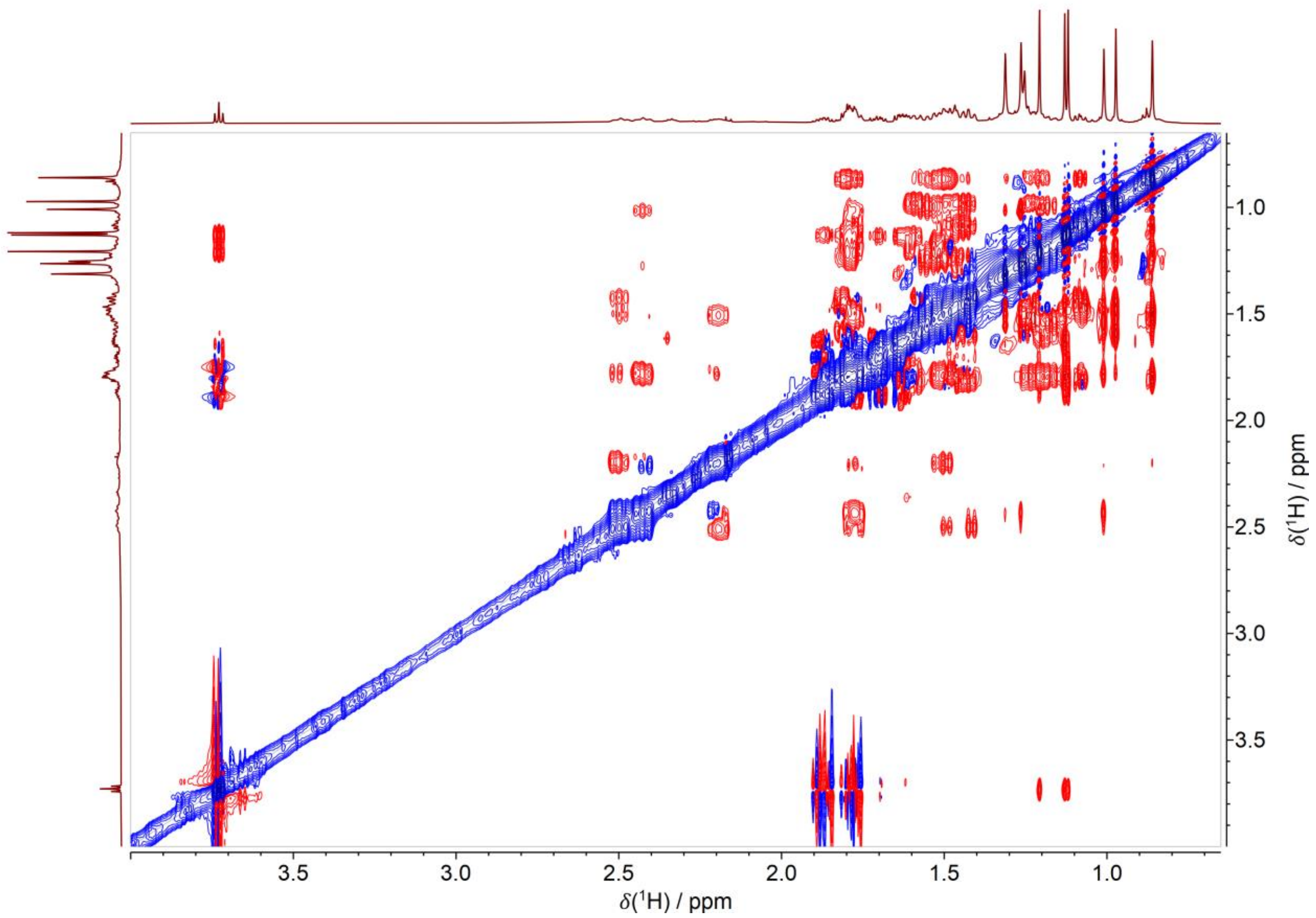

Figure S29. ^{1}H–^{1}H ROESY NMR spectrum of foveolin C (**2**) (600 MHz, $CDCl_3$).

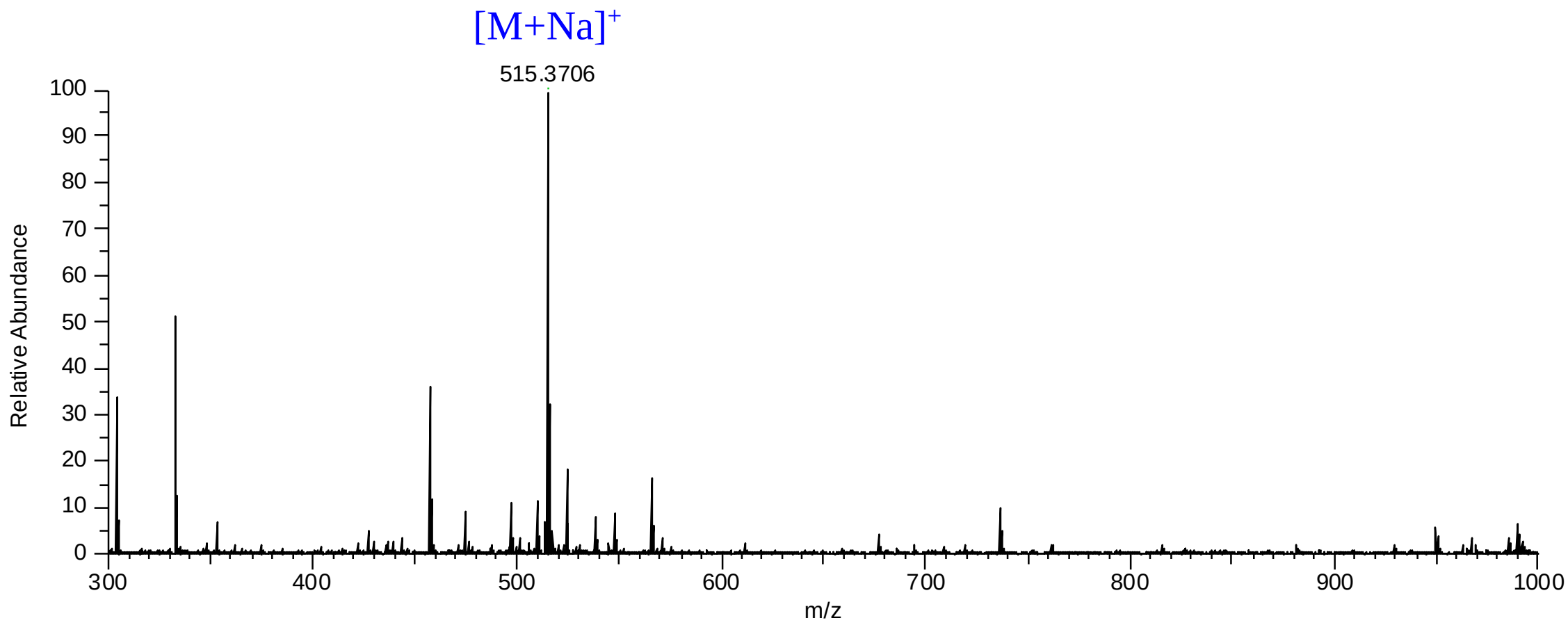

Figure S30. HR-ESI$^+$-MS spectrum of foveolin C (**2**), *m/z* 515.3706 [M+Na]$^+$ (calculated for $C_{30}H_{52}O_5Na^+$, *m/z* 515.3707 [M+Na]$^+$, error: −0.2 ppm).

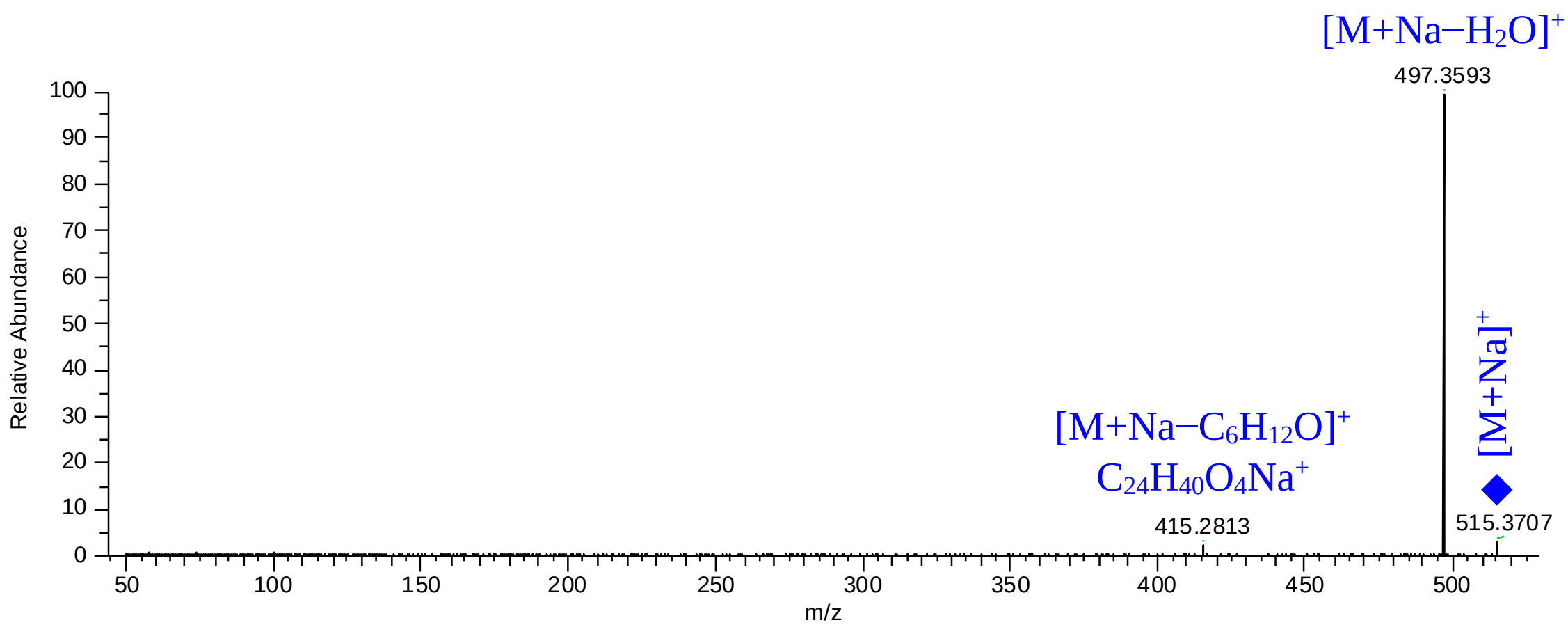

Figure S31. HR-ESI$^+$-MS/MS spectrum of foveolin C (**2**) with a normalized HCD collision energy of 50%. Precursor ion: *m/z* 515.3707 [M+Na]$^+$, $C_{30}H_{52}O_5Na^+$.

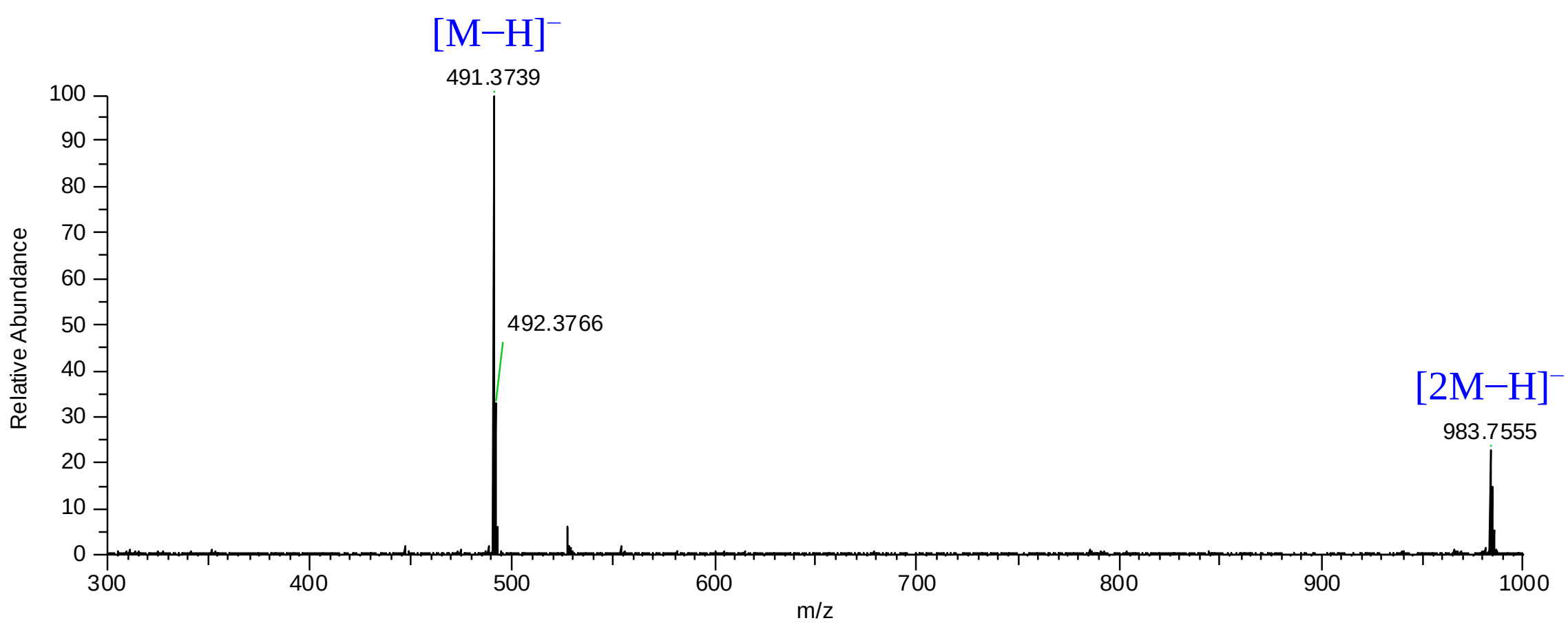

Figure S32. HR-ESI$^-$-MS spectrum of foveolin C (**2**), *m/z* 491.3739 [M−H]$^-$ (calculated for $C_{30}H_{51}O_5^-$, *m/z* 491.3742 [M−H]$^-$, error: −0.6 ppm).

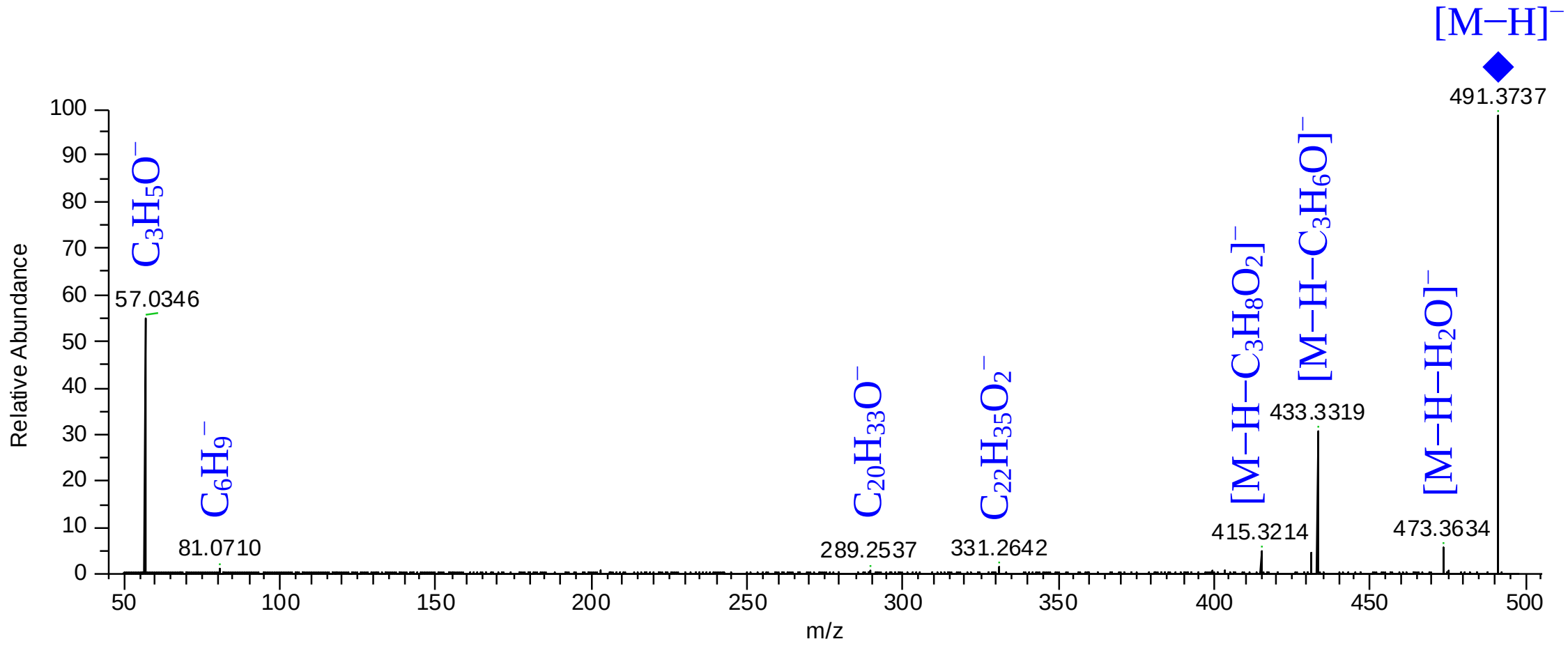

Figure S33. HR-ESI⁻-MS/MS spectrum of foveolin C (**2**) with a normalized HCD collision energy of 50%. Precursor ion: *m*/*z* 491.3737 [M−H]⁻, $C_{30}H_{51}O_5^-$.

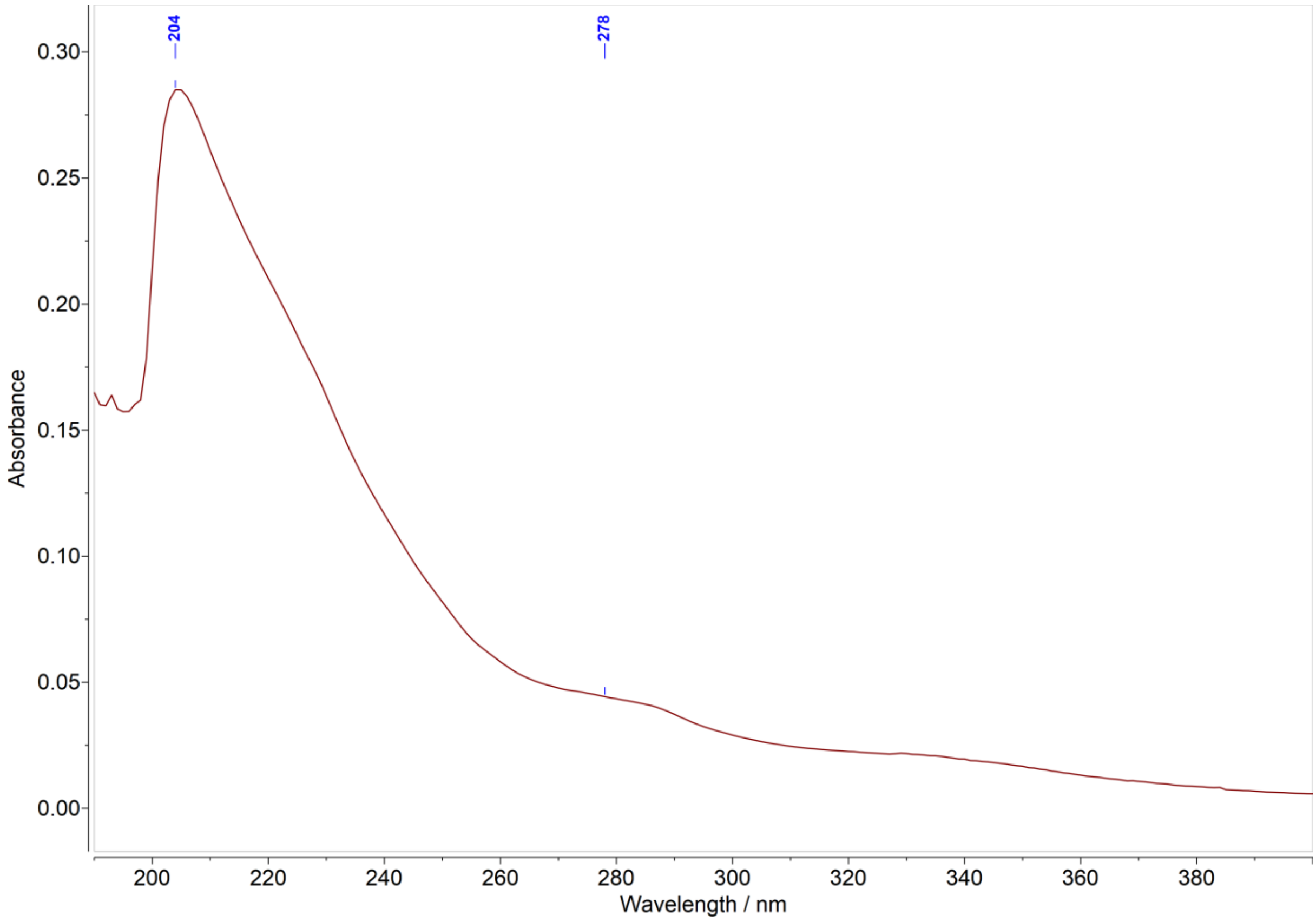

Figure S34. UV spectrum of foveolin C (**2**) in ethanol.

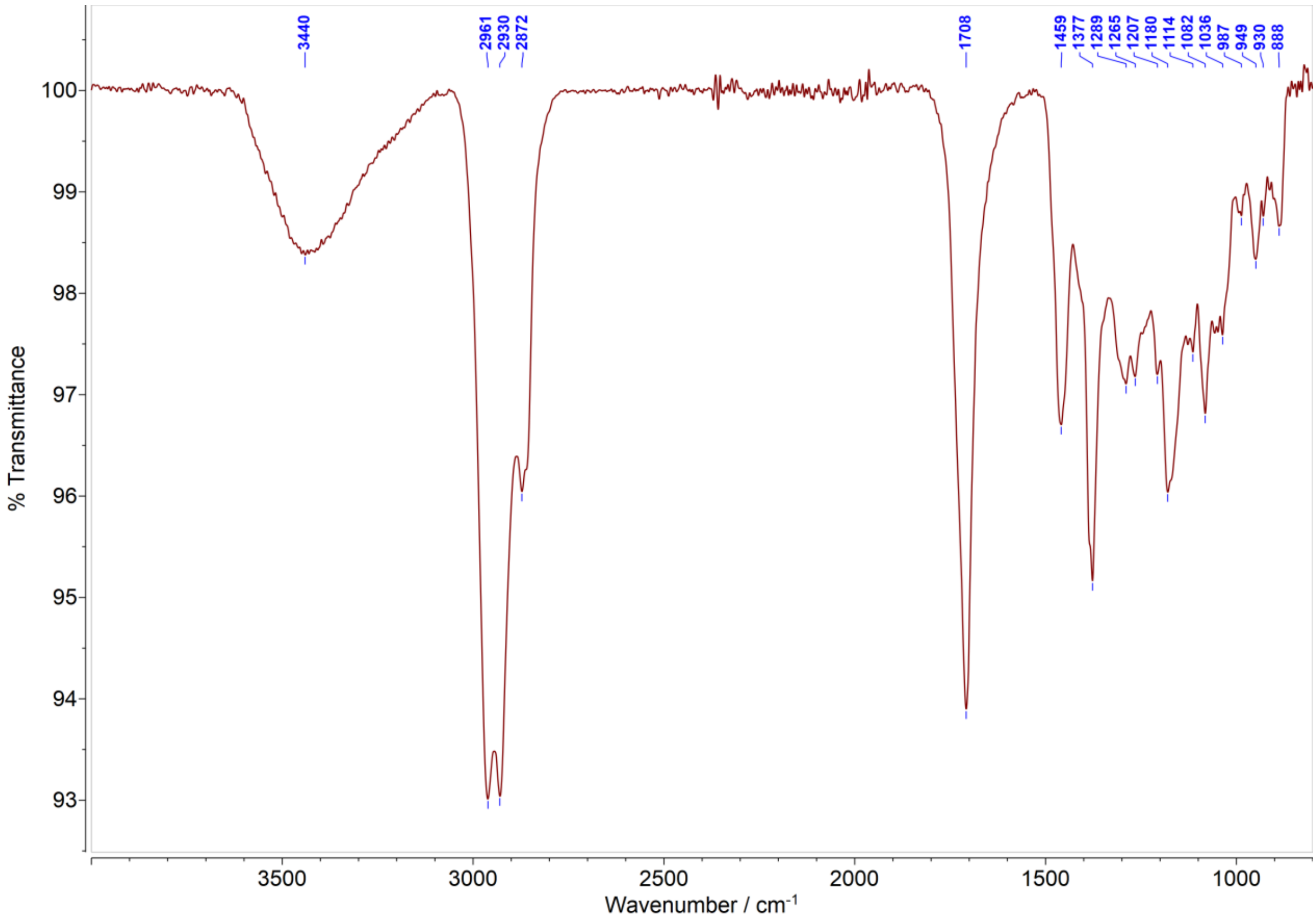

Figure S35. ATR-FTIR spectrum of foveolin C (**2**).

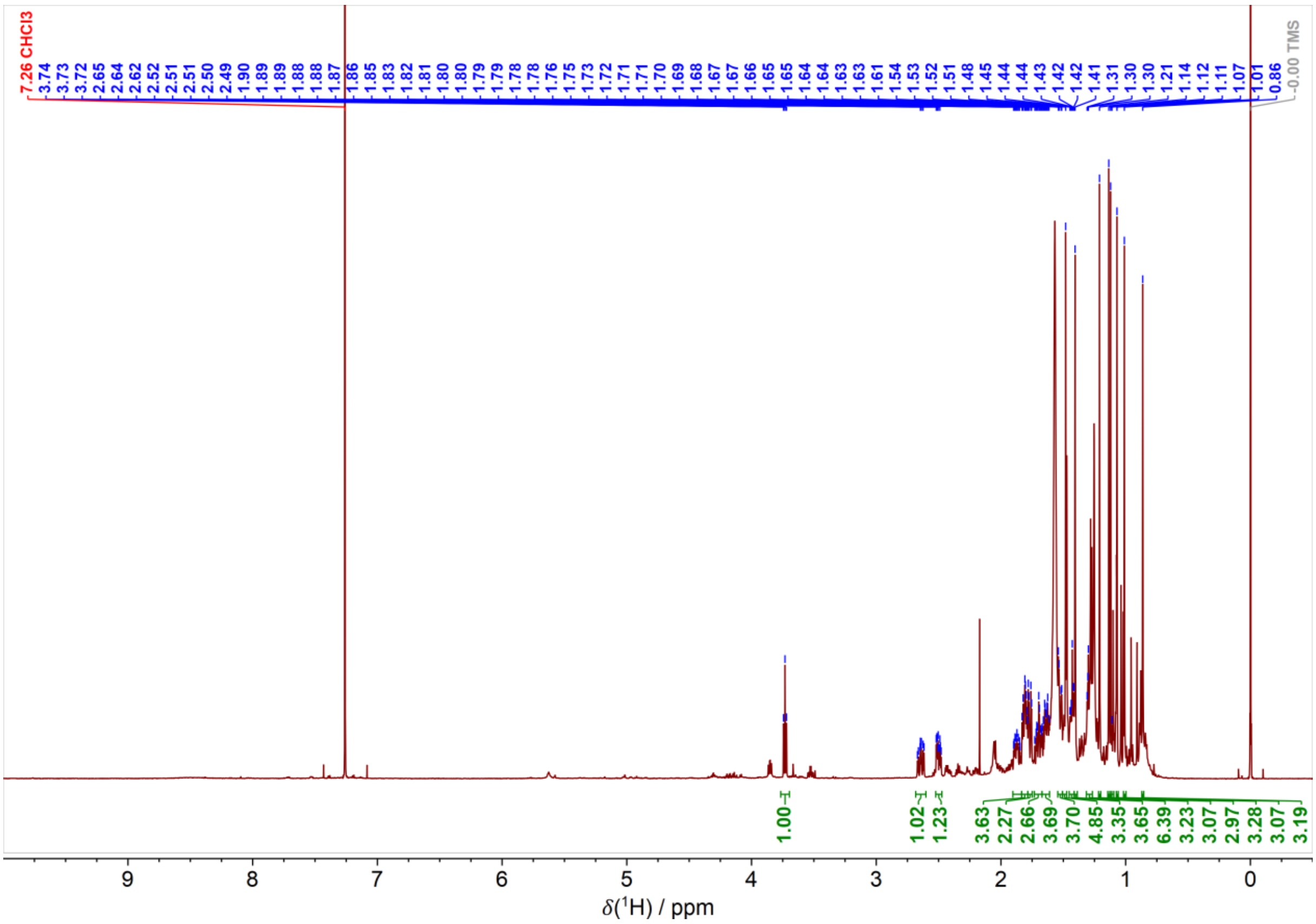

Figure S36. ^{1}H NMR spectrum of (20*S*,24*R*)-epoxy-25-hydroxy-A-homo-4-oxadammaran-3-one (**3**) (600 MHz, $CDCl_3$).

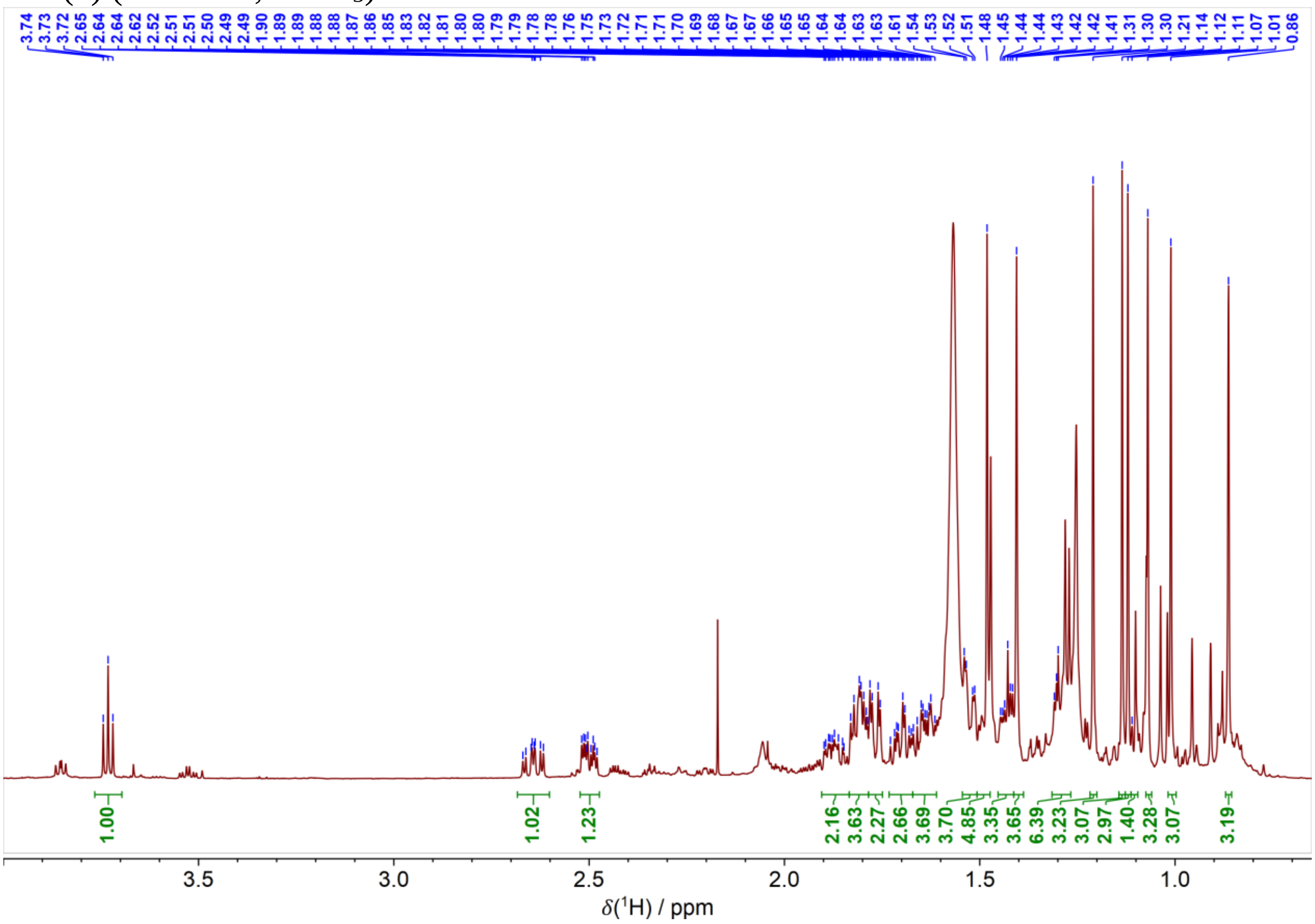

Figure S37. ^{1}H NMR spectrum of (20*S*,24*R*)-epoxy-25-hydroxy-A-homo-4-oxadammaran-3-one (**3**) (600 MHz, $CDCl_3$) – expansion (δ_H 4.0–0.65).

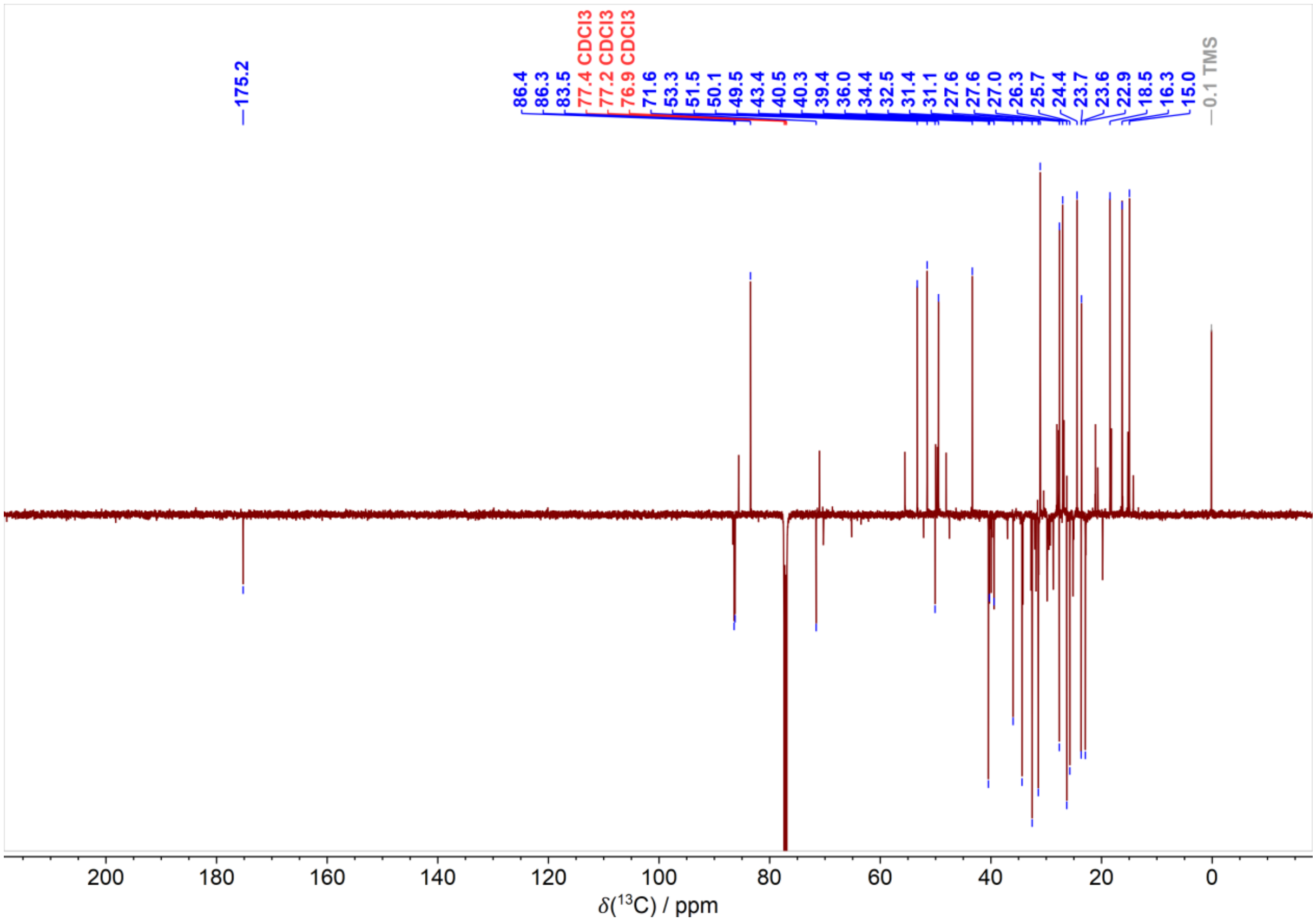

Figure S38. ^{13}C DEPTQ NMR spectrum of (20*S*,24*R*)-epoxy-25-hydroxy-A-homo-4-oxadammaran-3-one (**3**) (151 MHz, $CDCl_3$).

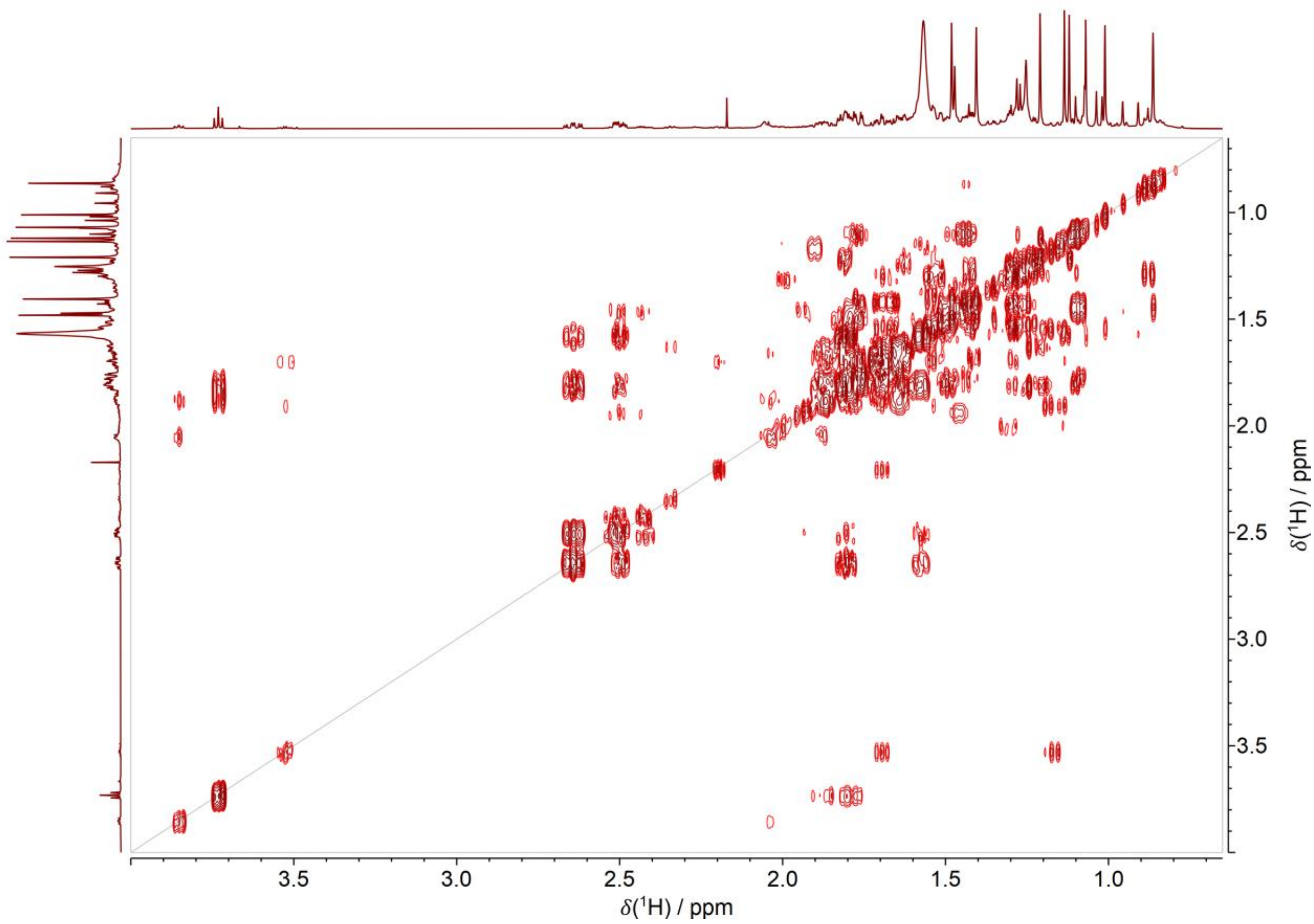

Figure S39. ^{1}H–^{1}H COSY NMR spectrum of (20*S*,24*R*)-epoxy-25-hydroxy-A-homo-4-oxadammaran-3-one (**3**) (600 MHz, $CDCl_3$).

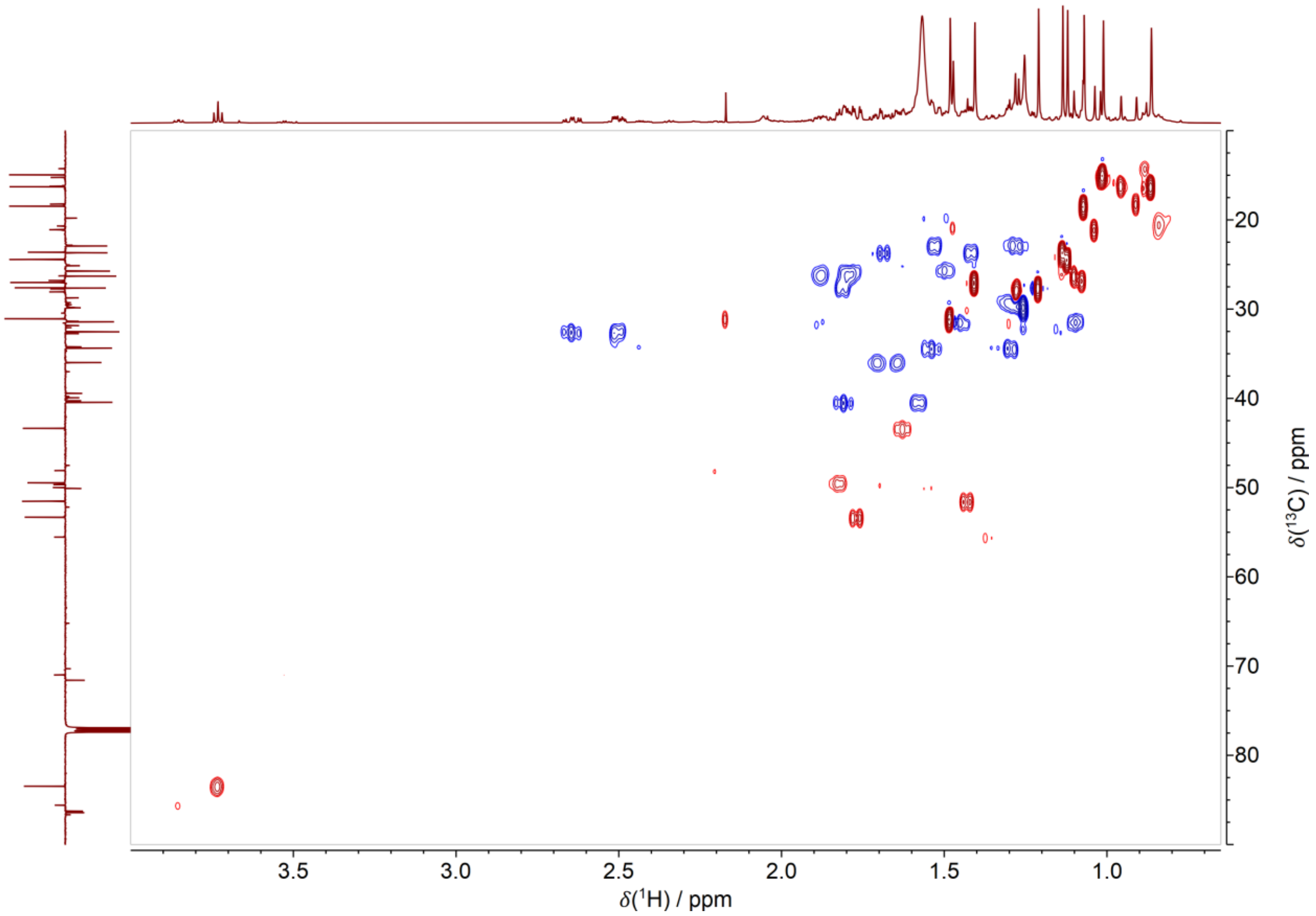

Figure S40. $^{1}H–^{13}C$ multiplicity-edited HSQC NMR spectrum of (20*S*,24*R*)-epoxy-25-hydroxy-A-homo-4-oxadammaran-3-one (**3**) (600/151 MHz, $CDCl_3$).

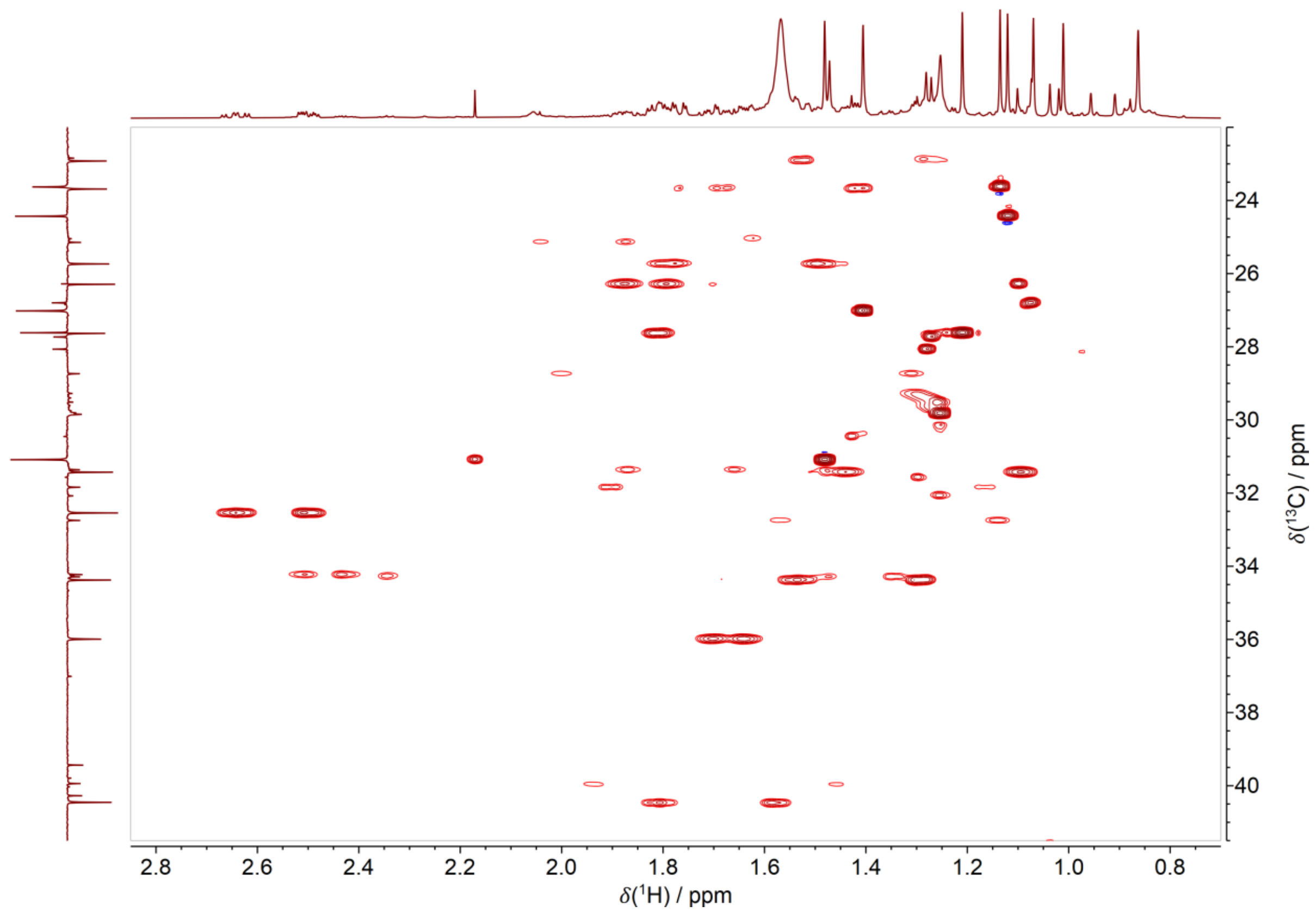

Figure S41. $^{1}H–^{13}C$ band-selective HSQC NMR spectrum of (20*S*,24*R*)-epoxy-25-hydroxy-A-homo-4-oxadammaran-3-one (**3**) (600/151 MHz, $CDCl_3$) – aliphatic region (δ_C 41.5–22.0).

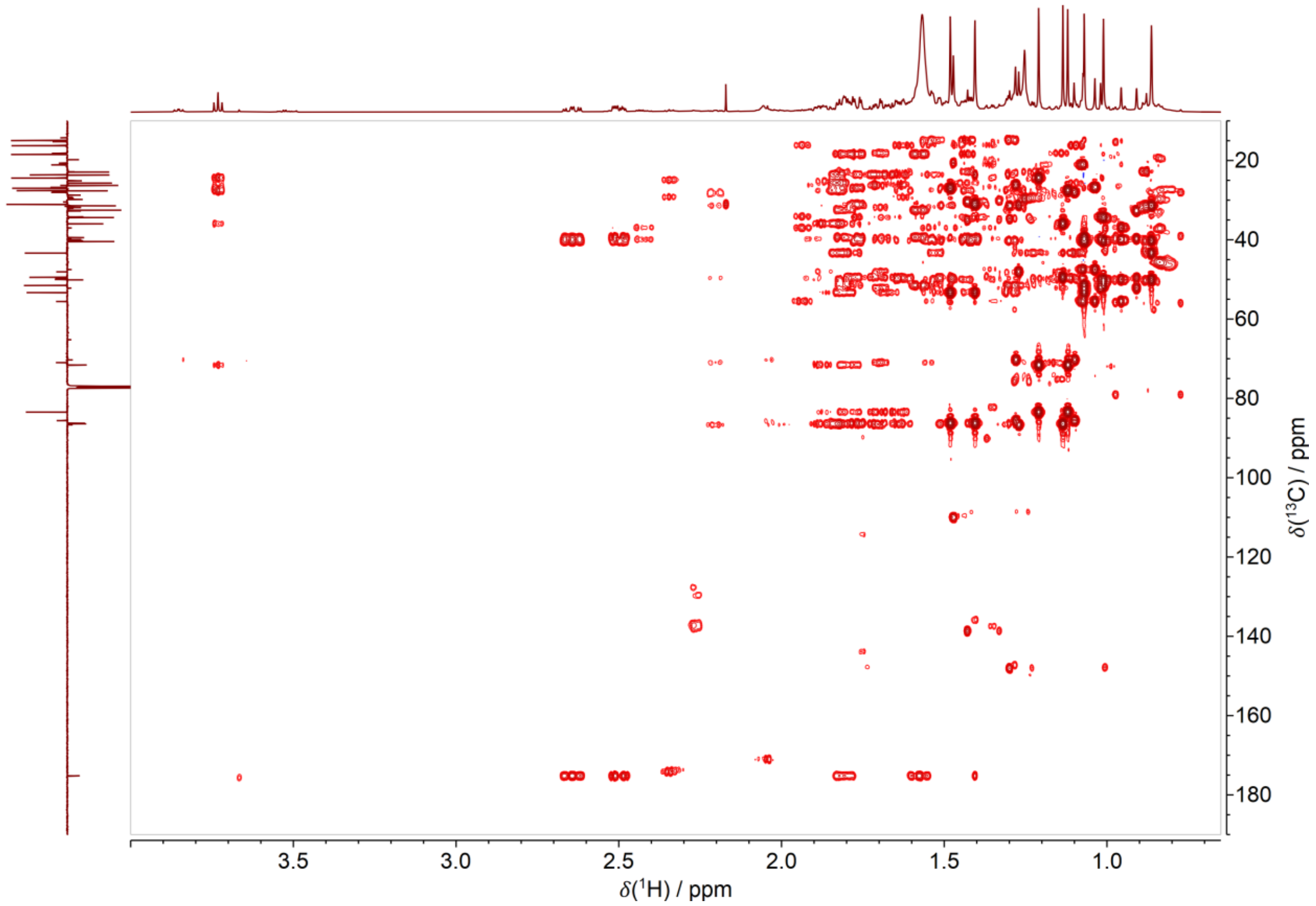

Figure S42. ^{1}H–^{13}C HMBC NMR spectrum of (20*S*,24*R*)-epoxy-25-hydroxy-A-homo-4-oxadammaran-3-one (**3**) (600/151 MHz, $CDCl_3$).

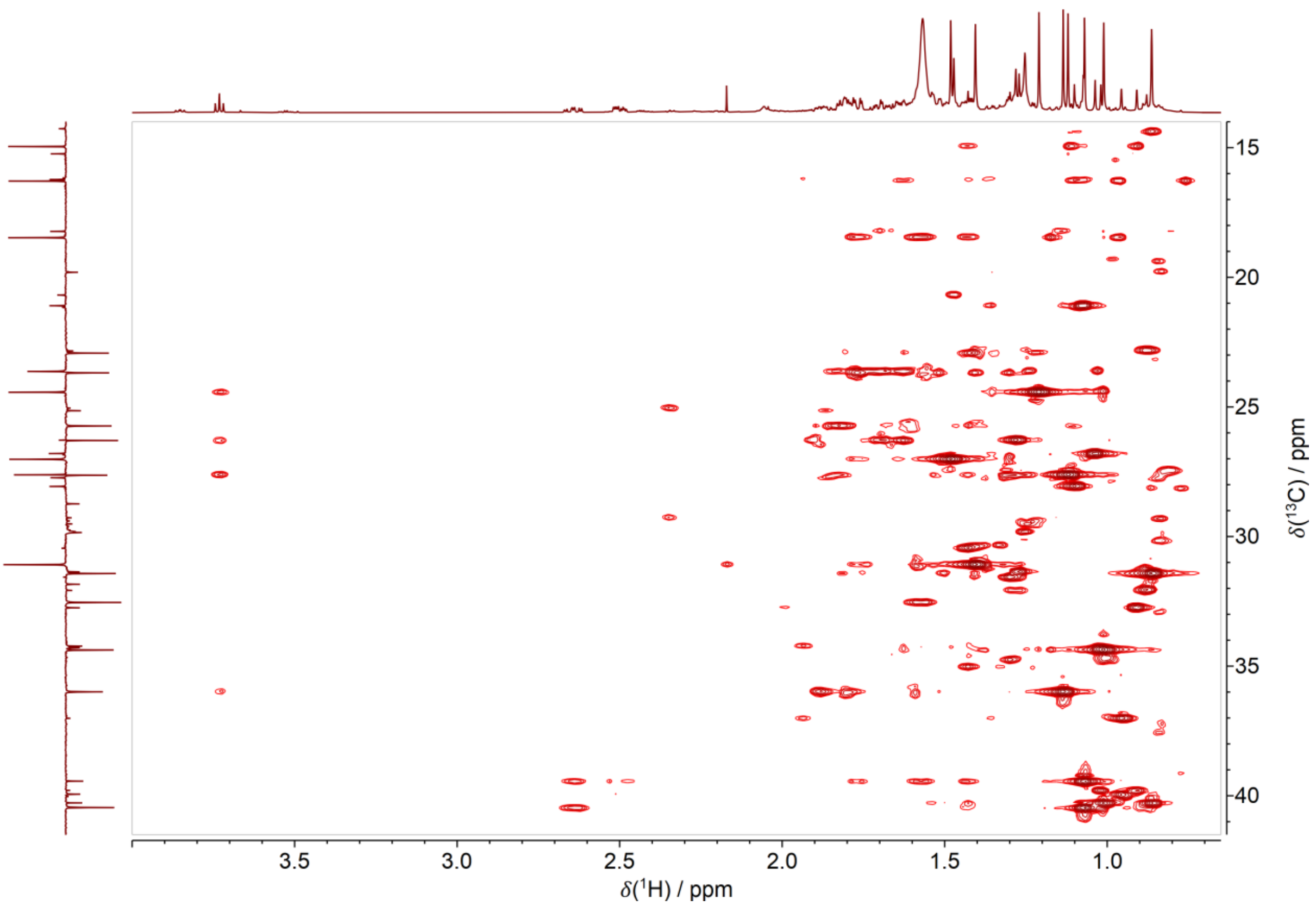

Figure S43. ^{1}H–^{13}C band-selective HMBC NMR spectrum of (20*S*,24*R*)-epoxy-25-hydroxy-A-homo-4-oxadammaran-3-one (**3**) (600/151 MHz, $CDCl_3$) – aliphatic region (δ_C 41.5–14.0).

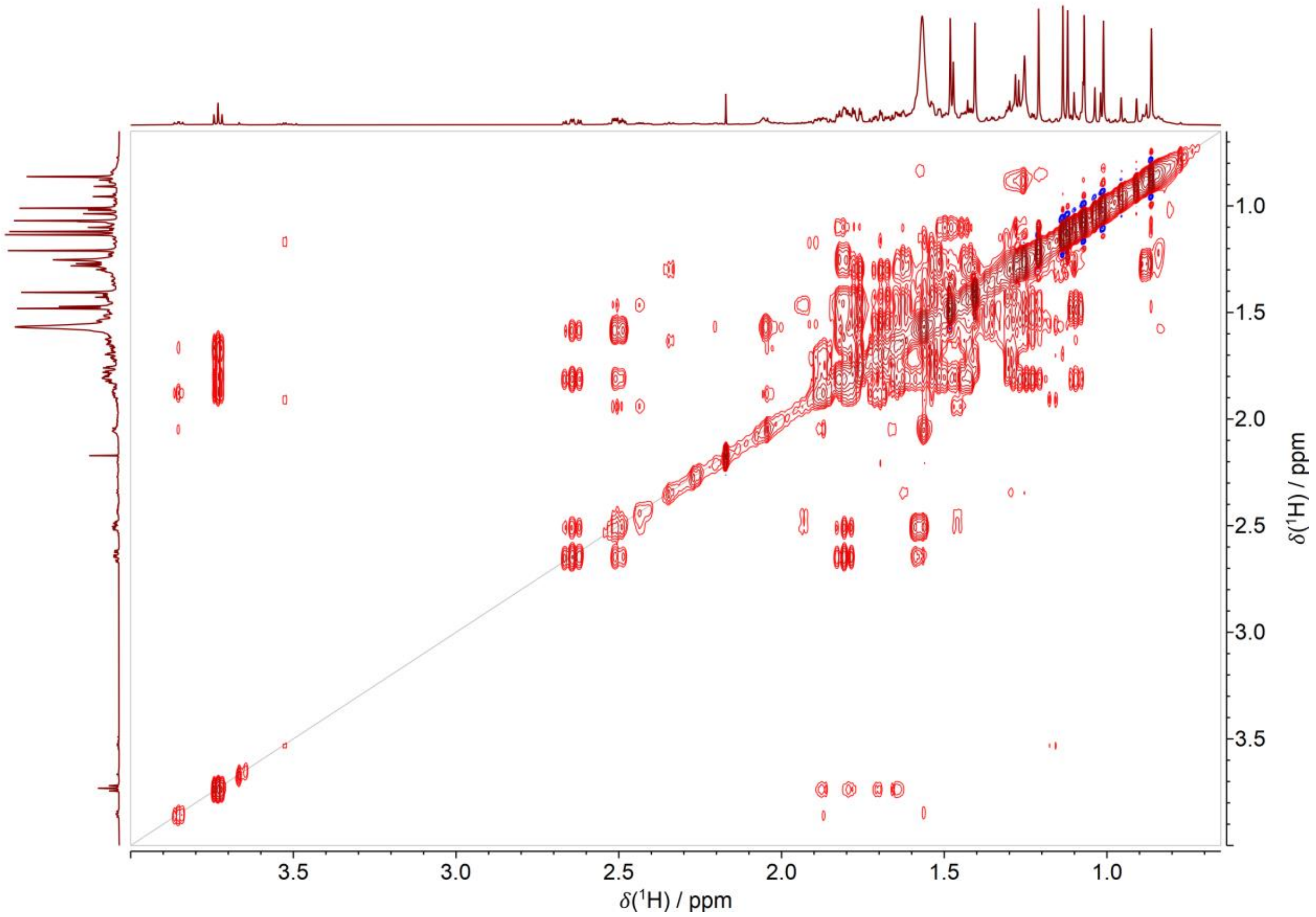

Figure S44. ^{1}H–^{1}H TOCSY NMR spectrum of (20*S*,24*R*)-epoxy-25-hydroxy-A-homo-4-oxadammaran-3-one (**3**) (600 MHz, $CDCl_3$).

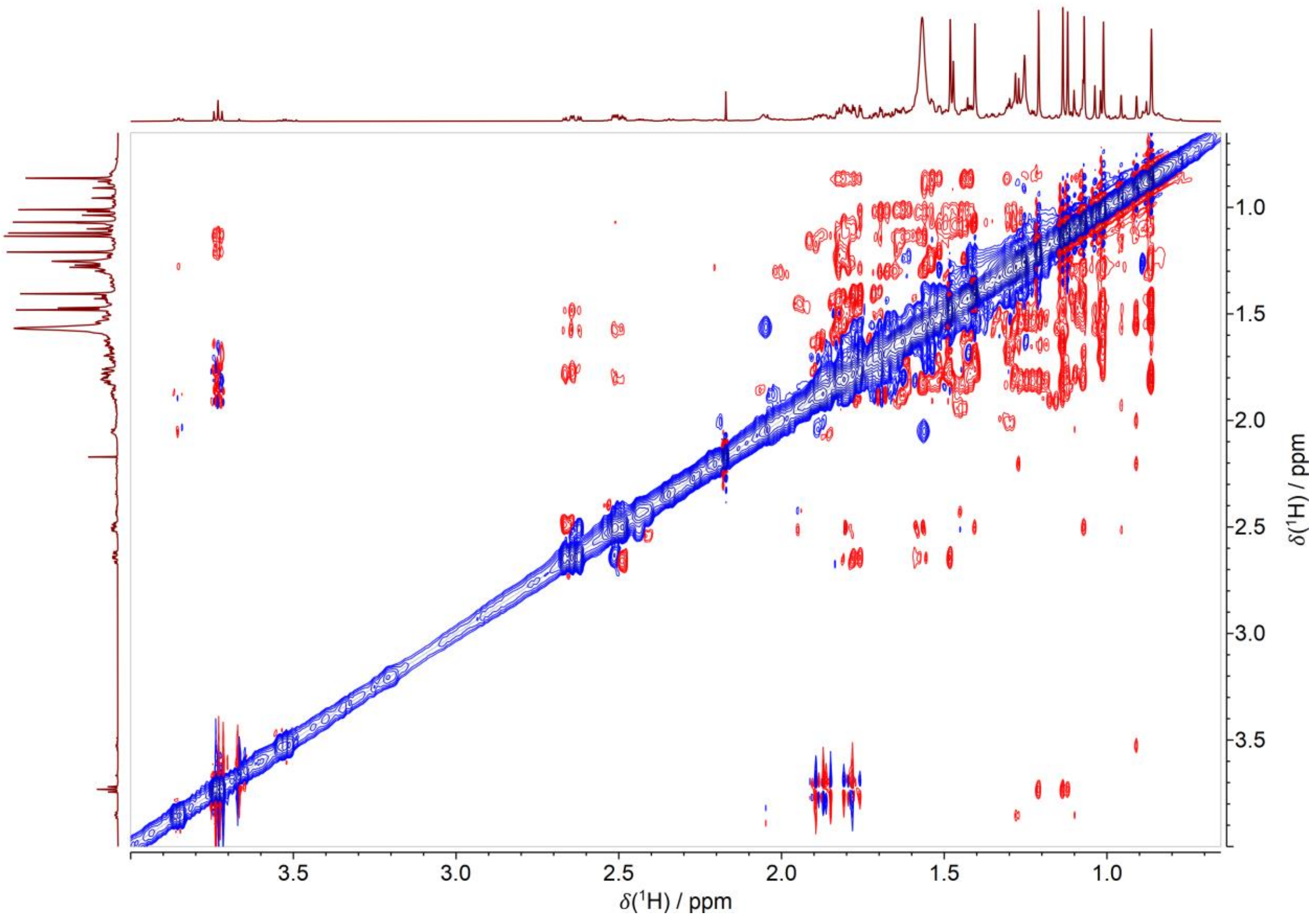

Figure S45. ^{1}H–^{1}H ROESY NMR spectrum of (20*S*,24*R*)-epoxy-25-hydroxy-A-homo-4-oxadammaran-3-one (**3**) (600 MHz, $CDCl_3$).

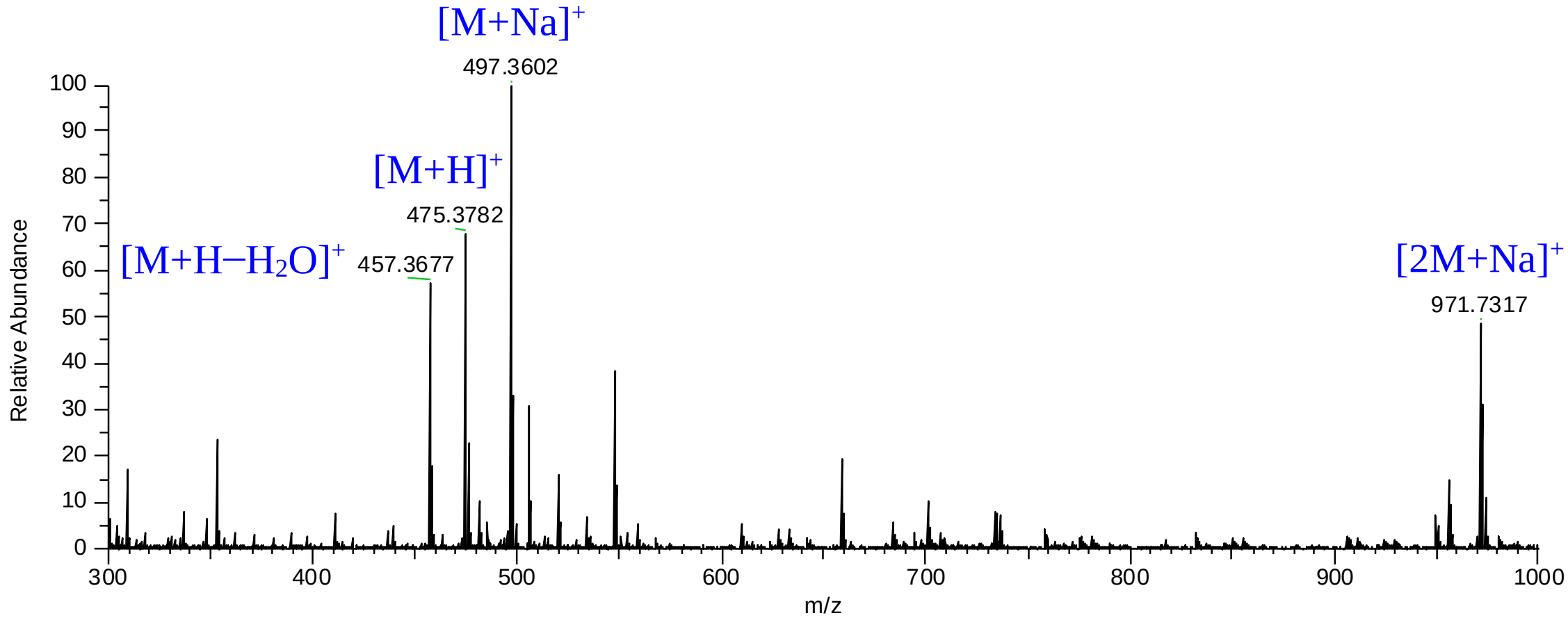

Figure S46. HR-ESI+-MS spectrum of (20*S*,24*R*)-epoxy-25-hydroxy-A-homo-4-oxadammaran-3-one (**3**), *m/z* 497.3602 $[M+Na]^+$ (calculated for $C_{30}H_{50}O_4Na^+$, *m/z* 497.3601 $[M+Na]^+$, error: +0.1 ppm).

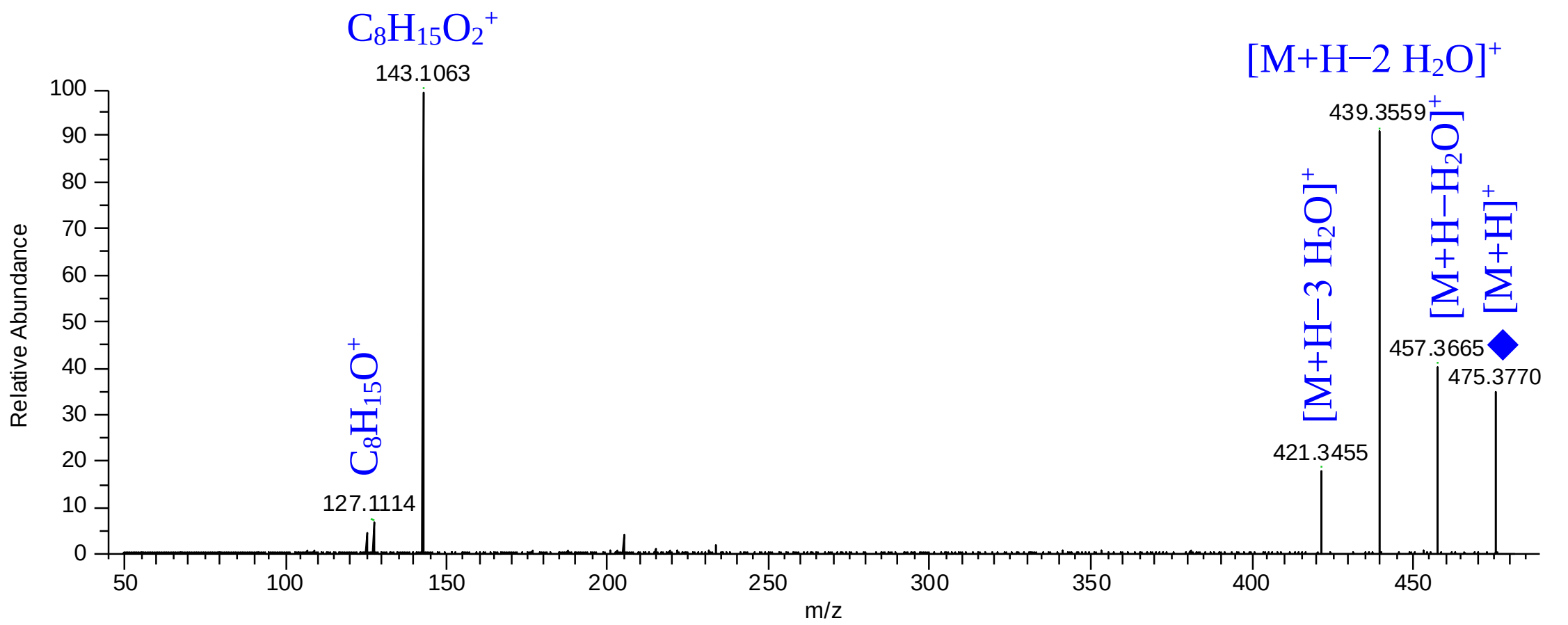

Figure S47. HR-ESI+-MS/MS spectrum of (20*S*,24*R*)-epoxy-25-hydroxy-A-homo-4-oxadammaran-3-one (**3**) with a normalized HCD collision energy of 15%. Precursor ion: *m/z* 475.3770 $[M+H]^+$, $C_{30}H_{51}O_4Na^+$.

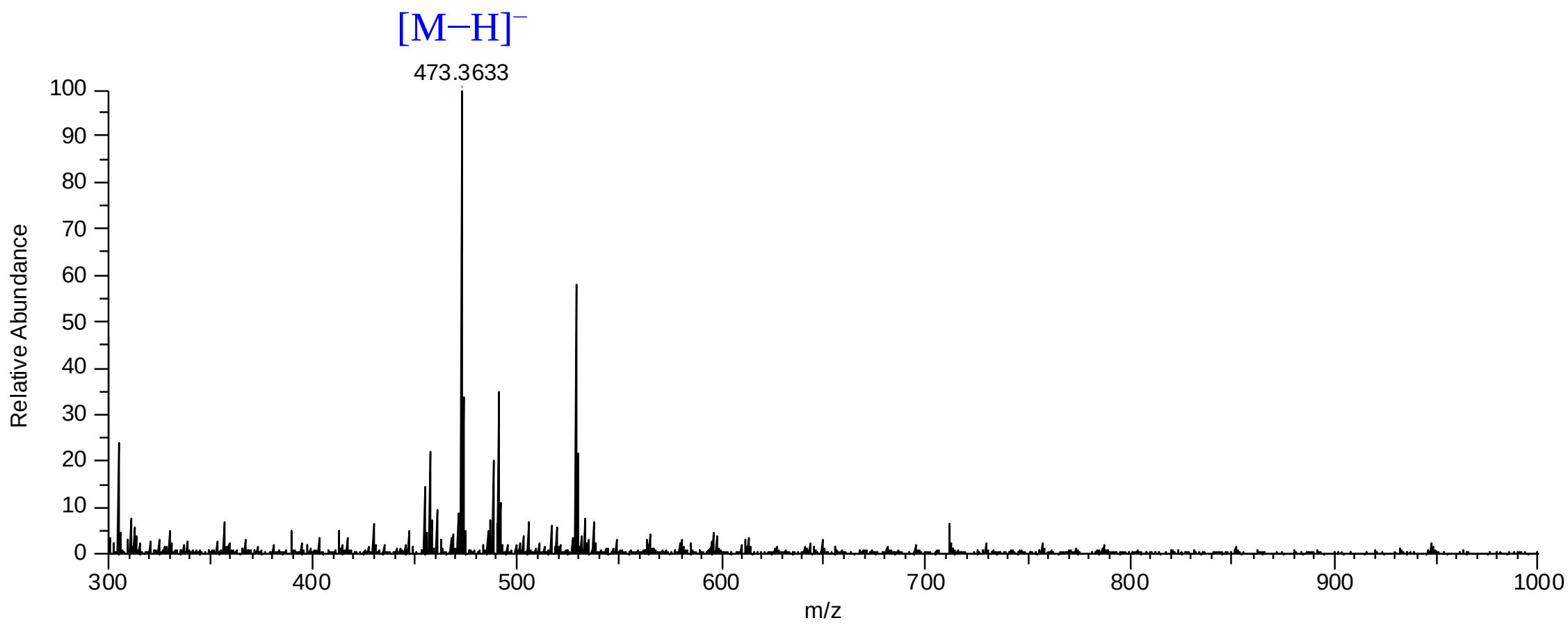

Figure S48. HR-ESI⁻-MS spectrum of (20*S*,24*R*)-epoxy-25-hydroxy-A-homo-4-oxadammaran-3-one (**3**), *m*/*z* 473.3633 $[M-H]^-$ (calculated for $C_{30}H_{49}O_4^-$, *m*/*z* 473.3636 $[M-H]^-$, error: −0.8 ppm).

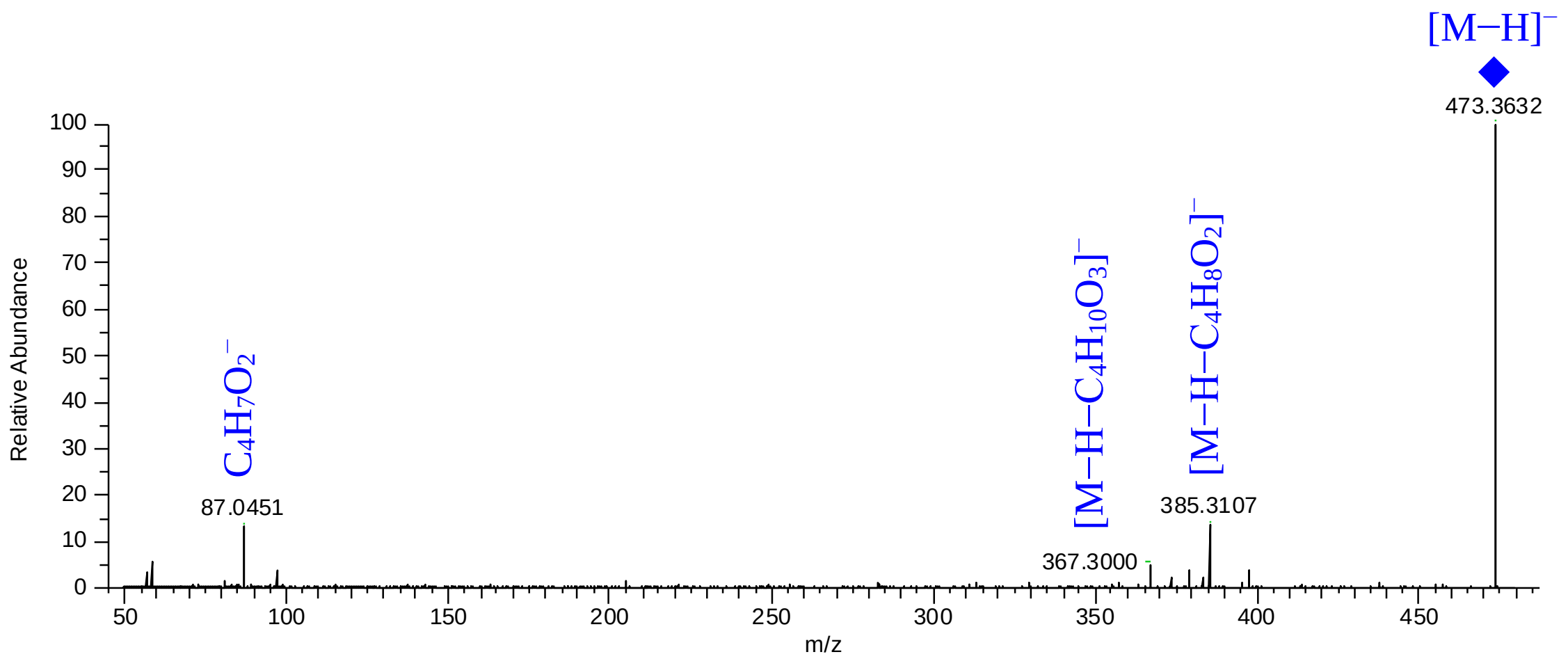

Figure S49. HR-ESI⁻-MS/MS spectrum of (20*S*,24*R*)-epoxy-25-hydroxy-A-homo-4-oxadammaran-3-one (**3**) with a normalized HCD collision energy of 50%. Precursor ion: *m*/*z* 473.3632 $[M-H]^-$, $C_{30}H_{49}O_4^-$.

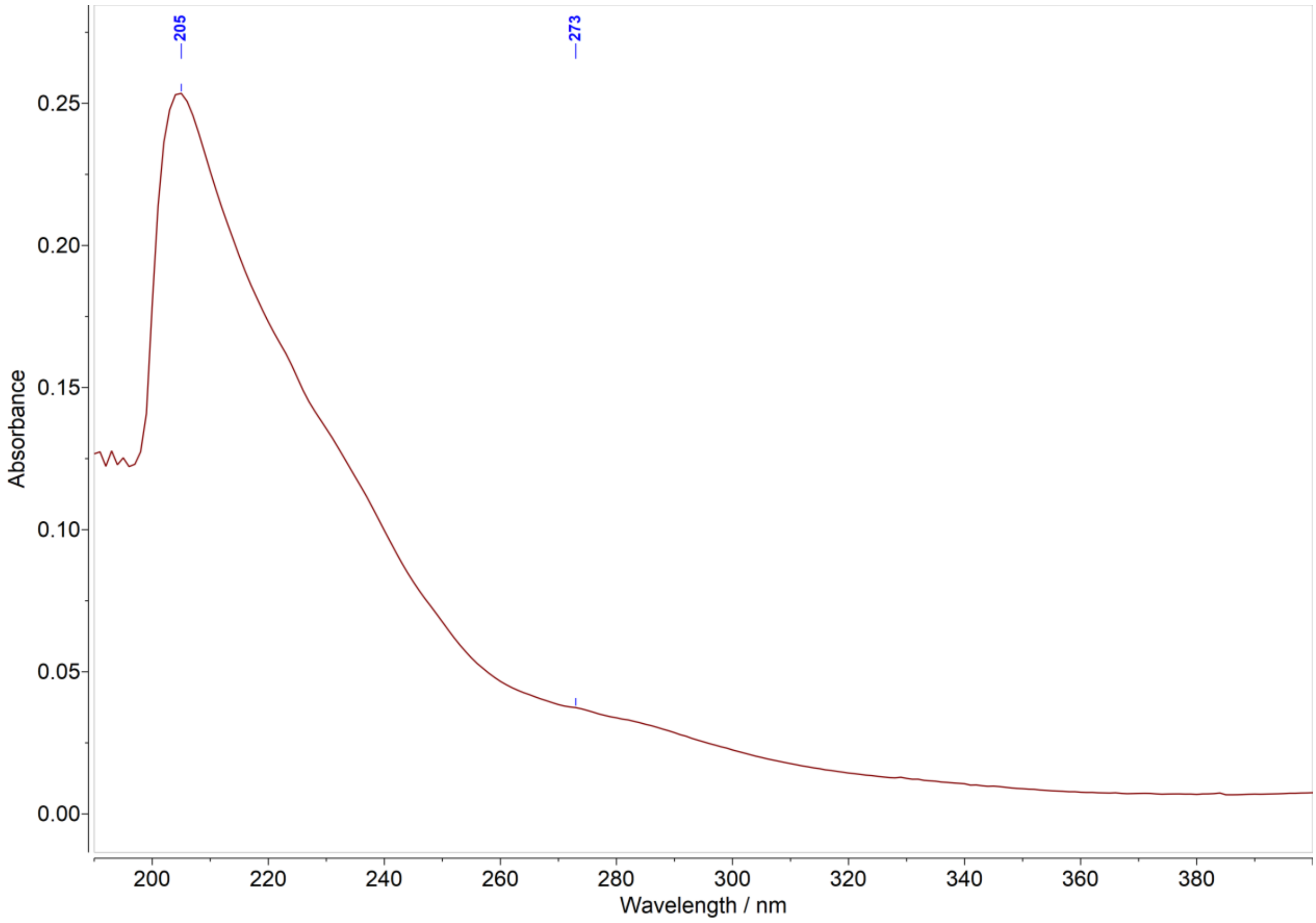

Figure S50. UV spectrum of (20*S*,24*R*)-epoxy-25-hydroxy-A-homo-4-oxadammaran-3-one (**3**) in ethanol.

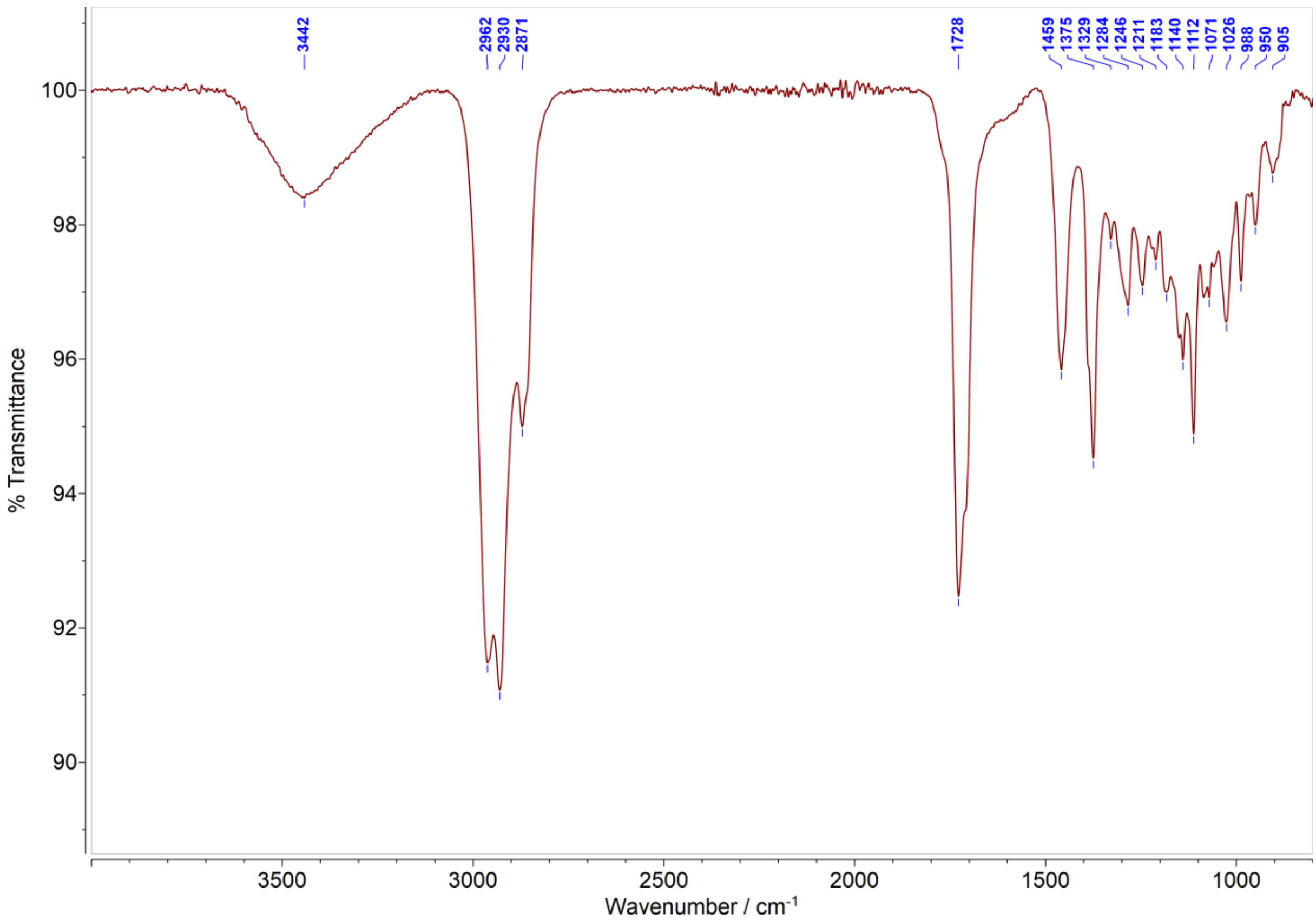

Figure S51. ATR-FTIR spectrum of (20*S*,24*R*)-epoxy-25-hydroxy-A-homo-4-oxadammaran-3-one (**3**).

References

Pan, L., Kardono, L.B.S., Riswan, S., Chai, H., Carcache De Blanco, E.J., Pannell, C.M., Soejarto, D.D., McCloud, T.G., Newman, D.J., Kinghorn, A.D. (2010). Isolation and characterization of minor analogues of silvestrol and other constituents from a large-scale re-collection of *Aglaia foveolata*. *J. Nat. Prod.* 73, 1873–1878. doi:10.1021/np100503q

Roux, D., Martin, M.-T., Adeline, M.-T., Sevenet, T., Hadi, A.H.A., Païs, M. (1998). Foveolins A and B, dammarane triterpenes from *Aglaia foveolata*. *Phytochemistry* 49, 1745–1748. doi:10.1016/S0031-9422(98)00305-7
